# Diversity and phage sensitivity to phages of porcine enterotoxigenic *Escherichia coli*

**DOI:** 10.1128/aem.00807-24

**Published:** 2024-06-28

**Authors:** Michela Gambino, Simran Krishnakant Kushwaha, Yi Wu, Pauline van Haastrecht, Victor Klein-Sousa, Veronika T. Lutz, Semeh Bejaoui, Christoffer Moeskjær C. Jensen, Martin S. Bojer, Wenchen Song, Minfeng Xiao, Nicholas M. I. Taylor, Franklin L. Nobrega, Lone Brøndsted

**Affiliations:** 1 Department of Veterinary and Animal Sciences, University of Copenhagen, Frederiksberg, Denmark; 2 Institute of Conservation, The Royal Danish Academy, Copenhagen, Denmark; 3 School of Biological Sciences, Faculty of Environmental & Life Sciences, University of Southampton, Southampton, United Kingdom; 4 Department of Biological Sciences, Birla Institute of Technology and Science, Pilani, Rajasthan, India; 5 Novo Nordisk Foundation Center for Protein Research, University of Copenhagen, Copenhagen, Denmark; 6 BGI-Shenzhen, Shenzhen, China; INRS Armand-Frappier Sante Biotechnologie Research Centre, Laval, Canada

**Keywords:** phages, enterotoxigenic *E. coli*, phage defense mechanisms, phage receptors

## Abstract

Enterotoxigenic *Escherichia coli* (ETEC) is a diverse and poorly characterized *E. coli* pathotype that causes diarrhea in humans and animals. Phages have been proposed for the veterinary biocontrol of ETEC, but effective solutions require understanding of porcine ETEC diversity that affects phage infection. Here, we sequenced and analyzed the genomes of the PHAGEBio ETEC collection, gathering 79 diverse ETEC strains isolated from European pigs with post-weaning diarrhea (PWD). We identified the virulence factors characterizing the pathotype and several antibiotic resistance genes on plasmids, while phage resistance genes and other virulence factors were mostly chromosome encoded. We experienced that ETEC strains were highly resistant to *Enterobacteriaceae* phage infection. It was only by enrichment of numerous diverse samples with different media and conditions, using the 41 ETEC strains of our collection as hosts, that we could isolate two lytic phages that could infect a large part of our diverse ETEC collection: vB_EcoP_ETEP21B and vB_EcoS_ETEP102. Based on genome and host range analyses, we discussed the infection strategies of the two phages and identified components of lipopolysaccharides ( LPS) as receptors for the two phages. Our detailed computational structural analysis highlights several loops and pockets in the tail fibers that may allow recognition and binding of ETEC strains, also in the presence of O-antigens. Despite the importance of receptor recognition, the diversity of the ETEC strains remains a significant challenge for isolating ETEC phages and developing sustainable phage-based products to address ETEC-induced PWD.

**IMPORTANCE:**

Enterotoxigenic *Escherichia coli* (ETEC)-induced post-weaning diarrhea is a severe disease in piglets that leads to weight loss and potentially death, with high economic and animal welfare costs worldwide. Phage-based approaches have been proposed, but available data are insufficient to ensure efficacy. Genome analysis of an extensive collection of ETEC strains revealed that phage defense mechanisms were mostly chromosome encoded, suggesting a lower chance of spread and selection by phage exposure. The difficulty in isolating lytic phages and the molecular and structural analyses of two ETEC phages point toward a multifactorial resistance of ETEC to phage infection and the importance of extensive phage screenings specifically against clinically relevant strains. The PHAGEBio ETEC collection and these two phages are valuable tools for the scientific community to expand our knowledge on the most studied, but still enigmatic, bacterial species—*E. coli*.

## INTRODUCTION

Enterotoxigenic *Escherichia coli* (ETEC) is an intestinal opportunistic pathogen causing diarrhea in humans, cattle, and pigs with a substantial impact on human and animal health, society, and the economy. In pigs, ETEC is the main pathogen responsible for post-weaning diarrhea (PWD) (1, 2), a disease occurring within 10 days post-weaning, leading to dehydration, severe weight loss, and potentially death. The disease affects 20%–50% of weaned piglets, representing a major productive and economic loss in pig production worldwide (3, 4). Despite the societal impact, ETEC is one of the least studied pathotypes of *E. coli*. ETEC is defined by two host-specific virulence factors, fimbriae and enterotoxins, and porcine ETEC are thus different from human ETEC strains (5–7). Notably for the ecology of ETEC, both fimbriae and enterotoxins are often encoded by plasmids and other mobile genetic elements. Because of the crucial role of mobile genetic elements in their pathogenicity, ETEC strains represent a highly diverse pathotype and even strains belonging to the same phylogroup express diverse virulence factors, including toxins and colonization factors. ETEC infecting humans, for example, exhibit a remarkable sequence type and serotype diversity, and 25 different colonization factors have been reported so far (8, 9).

In porcine ETEC, fimbriae (commonly F4 and F18, but also F5, F6, and F17) recognize receptors exposed by the enterocytes in the small intestine of pigs after weaning. This binding subsequently triggers the release of enterotoxins (heat-labile toxin LT and heat-stable toxins ST1 and ST2) in the enterocytes, leading to fluid secretion and decreased water absorption in the gut lumen (2, 10). Since fimbriae and enterotoxins are often encoded by plasmids, porcine ETEC strains belong to several phylogenetic groups, including phylogroups A, B1, C, D, and E (11). Still, an association between fimbriae, serotype, and plasmid type has been observed. For example, in a collection of Danish ETEC strains, F4 strains were mainly O149 and O6 serotypes and harbored IncFII, IncFIB, and IncFIC plasmids, whereas F18 strains were O8 and O147 serotypes and harbored IncI1 and IncX1 plasmids (12). Recent findings also showed that the acquisition of virulence factors like fimbriae F4 and F18 is associated with antimicrobial resistance genes (specifically neomycin), suggesting co-transfer or co-location on the same plasmid (13). This observation is particularly worrisome since ETEC-induced PWD is one of the main reasons for antimicrobial use in livestock (14).

While genotyping, feeding, vaccines, and management strategies can reduce the impact of ETEC-induced PWD on pig farms (15–19), options for treatment are limited, especially after the restrictions on colistin and zinc oxide use for veterinary applications (14, 20). Alternatives are urgently needed, but efficacy in the long term and on a broad scale requires a deep understanding of the target organism. For phage therapy targeting porcine ETEC, this can be translated into characterizing a broad collection of porcine ETEC strains and defining the molecular mechanisms used by phages (short for bacteriophages, viruses that infect bacteria) to recognize and infect their host. Phages have been proposed and, in some cases, already used as alternatives to antimicrobials to treat or prevent several human and animal infections (21, 22).

Phages infecting *E. coli* are among the most isolated and best-characterized phages, including some still used as models for molecular biology studies (23). Nevertheless, most coliphages have been isolated on laboratory strains and, being phages extremely specific for their hosts, do not necessarily infect wild-type or pathogenic strains (24). To the best of our knowledge, only 30 phages have been reported to infect porcine ETEC strains (25–35). Unfortunately, the data and information regarding these phages and their hosts are fragmentary. For example, phage genome sequences are publicly available only for 10 lytic phages, and only two studies report the virulence factors that classify the infected strains as ETEC (27, 31). This incomplete data set fails to link the molecular aspects of phage infection to the ETEC strains, which is urgently needed to propose an efficient phage product on an international scale.

Here, we present a well-characterized collection of porcine ETEC strains intended as a reference for designing novel alternative antibacterial solutions and studying *E. coli* diversity, phages, and mobile genetic elements. In addition, we isolated two novel virulent phages infecting strains of the ETEC collection and characterized the receptor binding apparatus of the phages, allowing us to explain their ability to infect porcine ETEC.

## RESULTS

### The PHAGEBio collection of porcine ETEC strains

**Fig 1:**
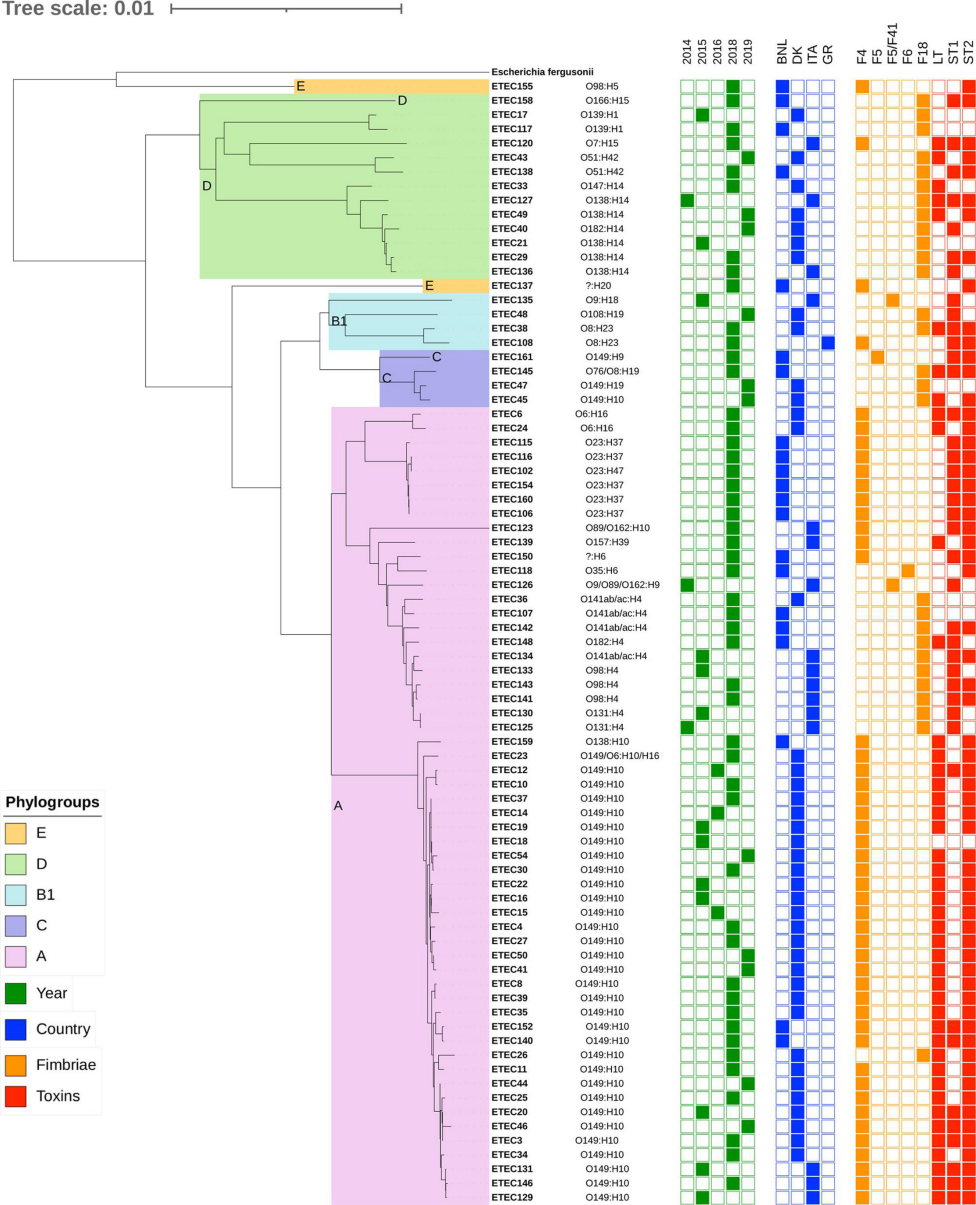
Phylogenetic tree built with the core genomes of the ETEC collection. Colors on the clades indicate the phylogroups, determined by submitting the ECOR genome sequences to EzClermont (https://ezclermont.hutton.ac.uk/). From left to right, we report the serotype (O- and H-type), year (green), country (blue), fimbriae (orange), and toxins (red).

To propose a relevant collection of porcine ETEC strains, we gathered 79 hemolytic strains, isolated from diverse pig farms in Europe (Denmark, Italy, Greece, Belgium, and Netherlands) between 2014 and 2020, from fecal or gut samples of pigs clinically affected by PWD. Using multiplex PCR (7), the strains were confirmed to encode for fimbriae but not shigatoxins or verotoxins that would assign the strains to other *E. coli* pathotypes. F4 fimbriae were identified in the majority of the ETEC strains (*n* = 47), followed by F18 (*n* = 28), whereas only four strains encoded other fimbria types (i.e., F5, F5/F41, or F6). Except for seven strains for which no enterotoxins have been identified, enterotoxins are present alone (*n* = 12) or in different combinations (*n* = 60), with the most common combination being ST2, LT (*n* = 29) (Fig. 1; supplemental file S1A).

All ETEC genomes are available on NCBI (BioProject ID: PRJNA770188) as the PHAGEBio ETEC collection. The considerable variation in genome sizes (from 4.9 to 6.2 Mb) and the small size of the core genome (13% of the total genes) confirmed the polyphyletic origin of the ETEC strains. We determined the phylogroups of the ETEC strains (Fig. 1) and compared with the *E.coli* strains in the ECOR collection (supplemental file S1B) that represents the diversity of *E. coli*, and it is still used nowadays as a reference (36). The ETEC strains fell into phylogroups A, B1, C, D, and E, thus confirming the diverse genetic background of ETEC strains, as previously observed (9). The majority of the ETEC strains were assigned to phylogroup A (56 strains, 70.8%), while 13 belonged to phylogroup D (16.4%), 4 to phylogroup B1 (5.1%), the other 4 to C (5.1%), and 2 to E (2.5%).

The genetic diversity was also reflected in surface variation of these strains, as a total of 29 different serotypes were identified, resulting from a combination of 19 O-antigens and 17 H-antigens (Fig. 1; supplemental file S1A and B), with O149:H10 being by far the most common serotype (32 strains, 40.5%), followed by serotypes O23:H37 (6 strains) and O138:H14 (5 strains). All the strains with serotype O149:H10 fell into phylogroup A, where we also found the most common virotypes F4, ST2, and LT (25 strains, 31.6%). This collection well represents the extreme diversity of porcine ETEC strains in terms of genome, fimbriae, toxins, and O- and H- antigens (supplemental file S2).

Further bioinformatic analysis revealed that the contribution of MGEs to the diversity of the ETEC collection was considerable (supplemental files S1 and S2). For each ETEC strain, 15 to 82 contigs were predicted to belong to plasmids and 2 to 16 prophages. Virulence, metal, and antibiotic resistance genes were widespread, with virulence factors characterizing the pathotype and several antibiotic resistance genes on plasmids.

### ETECs are equipped with a large variety of phage defense mechanisms

**Fig 2:**
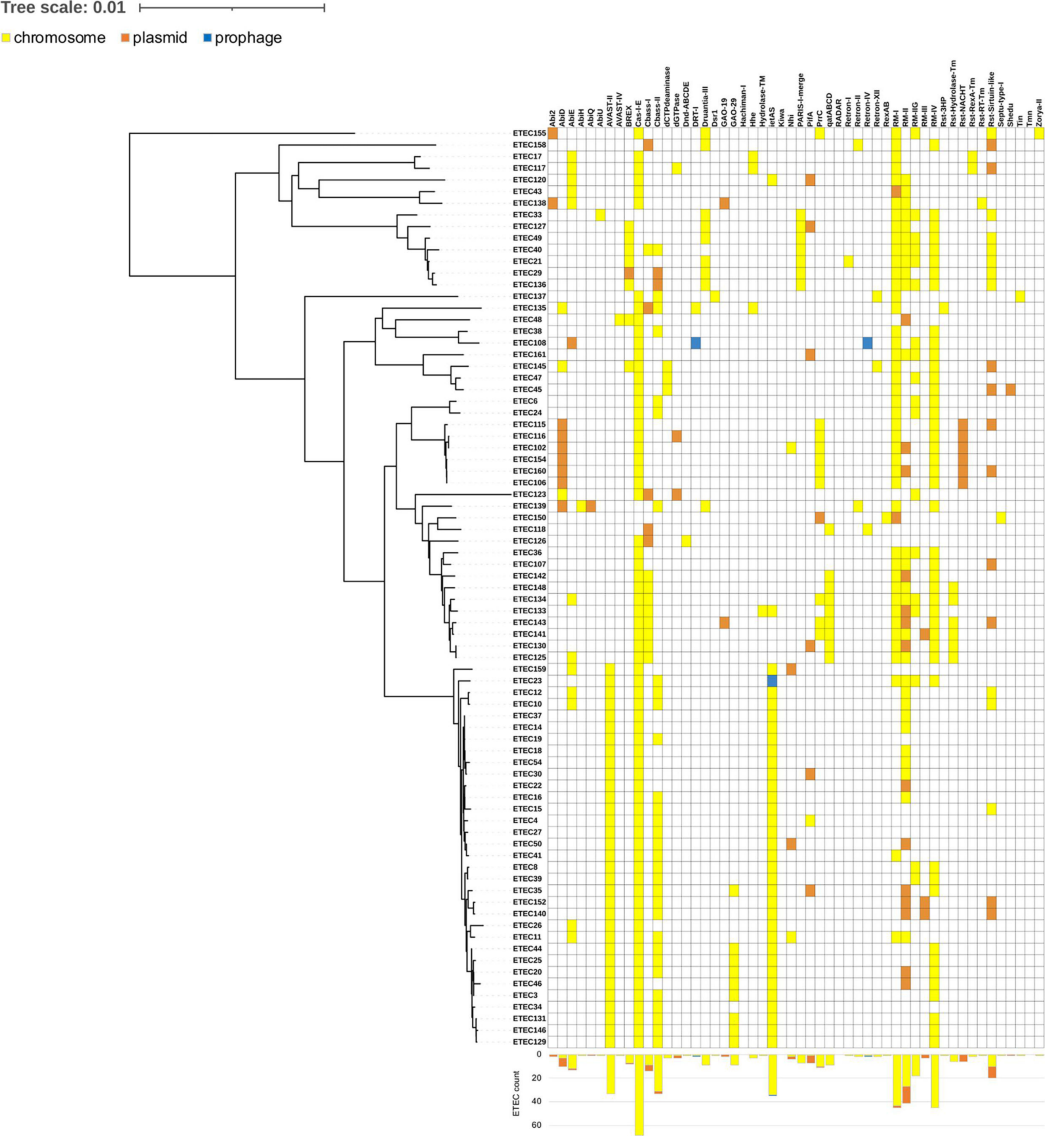
Phage defense mechanisms predicted in the ETEC collection. Colors indicate the predicted position of the gene: yellow on chromosomes, orange on plasmids, blue on prophages. The histograms at the bottom represent the count of ETEC strains where each defense system was detected.

Phage defense systems influence phage susceptibility, and PADLOC and DefenseFinder were used to predict their presence in the PHAGEBio ETEC collection. Most of the defense systems were on the chromosomes, and each strain encoded its own combination and types, thus reflecting ETEC genomic diversity (Fig. 2; supplemental file S2). The number of defense systems per genome varied widely from a minimum of four in several ETEC genomes to a maximum of 10 in ET134. There was a slight overrepresentation of defense systems in phylogroups D and E, compared with the other phylogroups, and the difference was maintained even when considering only the chromosome-encoded ones (supplemental file S1C through G).

The most widespread systems were known to defend the host by degradation of nucleic acids, such as CRISPR-Cas (with CRISPR-Cas I-E in 68 strains) and restriction modification systems (RM type I in 45 strains, RM type II in 41 strains, RM type IIG in 18 strains, RM type III in 3 strains, and RM type IV in 45 strains), or by abortive infection, such as CBASS (CBASS type I in 14 strains, CBASS type II in 33 strains) and probably AVAST (AVAST type II in 33 strains and AVAST type I in 1 strain) (Fig. 2). The mechanism of action of other similarly widespread systems is still unknown, such as in the case of *ietAS* (35 strains). Interestingly, while the CRISPR-Cas I-E system alone was detected in most strains, the RM types I and II presence complemented each other, occasionally with types IIG and III, and they overlapped in most strains with type IV. Moreover, other systems were mainly present in specific ETEC clades. For example, the three systems AVAST type II, *ietAS*, and CBASS type II seemed specific for a closely related clade in phylogroup A (supplemental file S2).

The only few defense systems predicted to be also on plasmids were many of the abortive infection systems (Abi2, AbiD, AbiE, AbiQ, CBASS-I, CBASS-II, PifA, and PrrC) and some of the ones degrading nucleic acids (Nhi, RM-I, RM-II, RM-III, and Shedu, 40), dGTPase and BREX preventing phage DNA replication (37), and the uncharacterized GAO-19, Rst NACHT, and Rst Sirtuin-like. In only three cases, the defense systems were predicted to be prophage encoded, i.e., *ietAS* in ET23 and DRT I and Retron IV in ET108 (Fig. 2). We can conclude that the diversity of phage resistance mechanisms and distribution in ETEC were linked more to chromosomal diversity of the strains than to MGEs.

### Two lytic phages infected 44% of the ETEC collection

**Fig 3:**
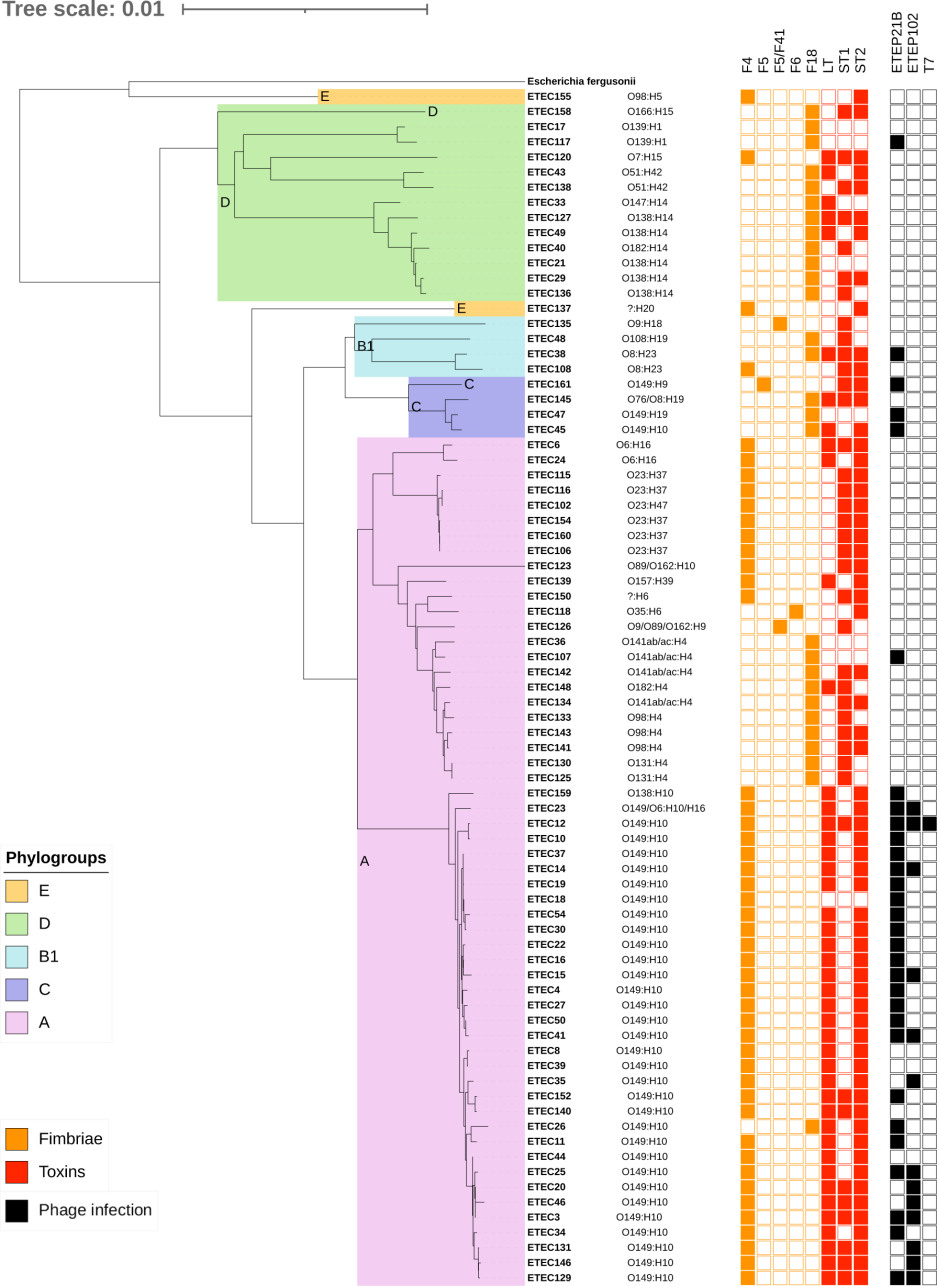
Host range analysis of the ETEC strains. For each strain, the phylogroup, the O-type, the virulence profile, and the infection by phages ETEP21B, ETEP102, and T7 are indicated.

To identify phages infecting ETEC, phage sensitivity of the ETEC strains has been tested by spotting previously isolated phages, such as 42 *Salmonella* phages (38, 39) and up to 200 *E. coli* phages, including the well-studied coliphages T3, T7, and CBA120, and others isolated on *E. coli* MG1655 and ECOR04. While spotting undiluted phage stocks, lysis spots were observed for many phage-host combinations. Still, none of the phages produced single plaques on any ETEC strains, except for phage T7 that infected ET12 (Fig. 3; supplemental file S3).

To isolate phages specifically infecting ETEC, 78 different samples (wastewater, pig manure, gut samples from pigs dying from ETEC diarrhea, and feces from healthy and ETEC-affected pigs) were screened for plaque formation by spotting directly and after enrichment using various media (LB, BHI, with or without mucin) on 41 Danish ETEC strains. More than 100 phage stocks were prepared, but most of the isolated phages most likely were temperate, as hypothesized by the turbidity of plaques. Indeed, sequencing a subset of them confirmed their lysogenic potential, since a simple RAST annotation revealed several genes predicted to encode for integrases, transposases, and repressor and derepressor proteins (an example in supplemental file S4). In addition, they resulted highly similar to a prophage in the ETEC strain UMNK88 [CP002729.1; 99% identity and query coverage (11)]. Given the envisioned future veterinary application targeting porcine PWD, we concentrated on the only two lytic phages isolated: ETEP21B and ETEP102. ETEP21B was isolated from a gut sample that also was the source of an ETEC strain (ET23) using ET14 as the isolation host and LB with mucin as media. ETEP102 was isolated from a gut sample that was the source of ETEC strain ET25 using ET03 as the isolation host and LB as media.

To unravel the mechanism used by phages ETEP21B and ETEP102 to infect ETEC, a host range analysis was performed, determining the ability to form plaques on lawns of the established ETEC collection, and found that 35 strains (44%) were sensitive to at least one of the phages (Fig. 3). Phage ETEP21B infected 30 strains (24 from Denmark, 6 from other countries), mostly of virotypes F4, ST2, LT, and O-type O149, but also seven F18 strains with other virotypes and O-types. Phage ETEP102 infected 13 ETEC strains (10 from Denmark, 3 from other countries), all F4 strains, including 5 additional strains that are resistant to ETEP21B infection (i.e., ET20, ET35, ET46, ET131, and ET146) (supplemental file S3). By comparing the host range of the two ETEC phages with the data collected on the ETEC strains (country, O-type, fimbriae, toxins, virulence factors, antibiotic and metal resistance genes, and phage defense mechanisms), no single variable responsible for phage infectivity or resistance could be identified. Thus, a combination of several factors most likely determines the host range of the phages.

### The two virulent ETEC phages belonged to different families

**Fig 4:**
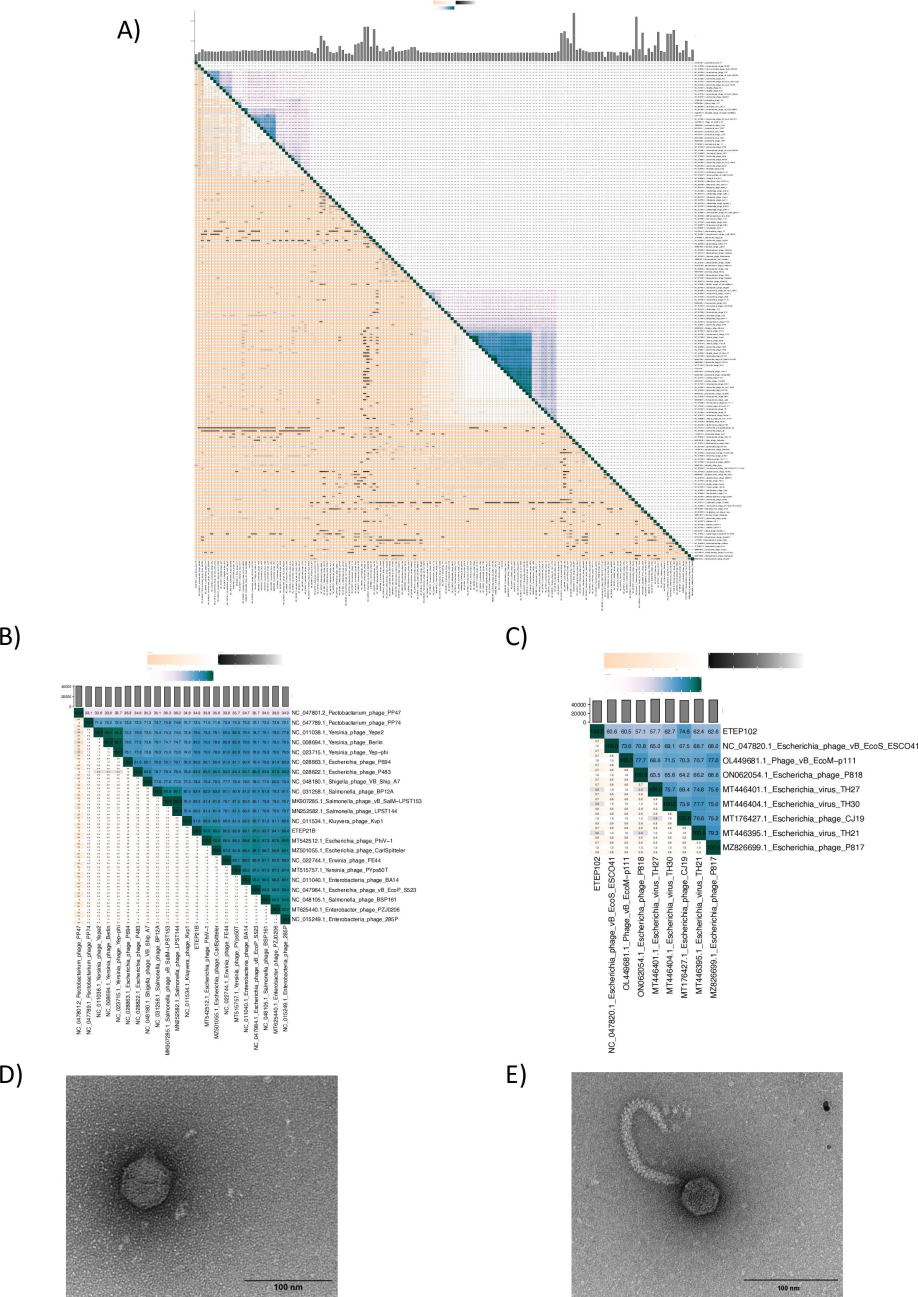
Two diverse phages infect the ETEC strains. (**A**) Intergenomic similarity between ETEP21B, ETEP102, and other 158 genomes (their closest relatives, one genome for each of the genera within the same family, and one genome for each of the other families as listed in ICTV). Close up of (**B**) ETEP21B and (**C**) ETEP102 clusters. For each graph: on the right side, darker colors indicate higher intergenomic similarity and the numbers represent the similarity values for each genome pair; on the left side, for each genome pair are the aligned fraction genome versus other genomes in the same row, in the same column and the genome length ratio. Transmission electron micrographs of (**D**) phage ETEP21B and (**E**) ETEP102. The bar represents 100 nm, as indicated.

Sequencing the genomes of ETEP21B and ETEP102 showed that both were dsDNA phages. While ETEP21B carried a genome of 39,424 bp with 49.1% GC content and encoded no tRNA, the genome of ETEP102 was 48,422 bp with 46.1% GC content and 1 tRNA binding L-Arginine. Comparison to the closest relatives to the two phage genomes using vCONTACT (supplemental file S5C) confirmed that the two phages were diverse and belonged to two different families (Fig. 4A). Phage ETEP21B was taxonomically assigned to the *Autographiviridae* family, *Studiervirinae* subfamily, *Berlinvirus* genus (Fig. 4B). ETEP21B closest relatives were other phages infecting *Enterobacteriaceae* (*Escherichia*, *Yersinia*, *Salmonella*, *Enterobacter*, and *Kluyvera* spp.) assigned to the *Berlinvirus* genus (supplemental file S5A), confirmed by ETEP21B sharing 87.36% average nucleotide identity (ANI) with *Yersinia* phage Berlin, reference for the *Berlinvirus* genus. In contrast, phage ETEP102 was assigned to the *Drexlerviridae* family (Fig. 4C), *Nouzillyvirus* genus, as it fell within the same vCONTACT subcluster as coliphage ESCO41. ETEP102 closest relatives were other phages infecting *E. coli*, likewise assigned to the *Nouzillyvirus* genus (supplemental file S5B). Despite the separate genus assigned by VIRIDIC clustering, ETEP102 shared 82.41% ANI with ESCO41 and was thus classified as a *Nouzillyvirus* phage. Transmission electron micrographs confirmed that the phages were diverse, showing two different morphotypes, with ETEP21B being a podovirus with an icosahedral head (59.7 ± 1.5 nm) and very short tail (12.0 ± 2.6 nm; Fig. 4D), while ETEP102 was a siphovirus with an icosahedral head (55.8 ± 9.0 nm and 63.4 ± 1.9 nm) and a long, flexible tail (158.5 ± 8.4 nm; Fig. 4E). Thus, we isolated two diverse phages, belonging to different families, yet both infecting porcine ETEC.

### The identification of the primary receptor of phage ETEP21B partially explained its host range

**Fig 5:**
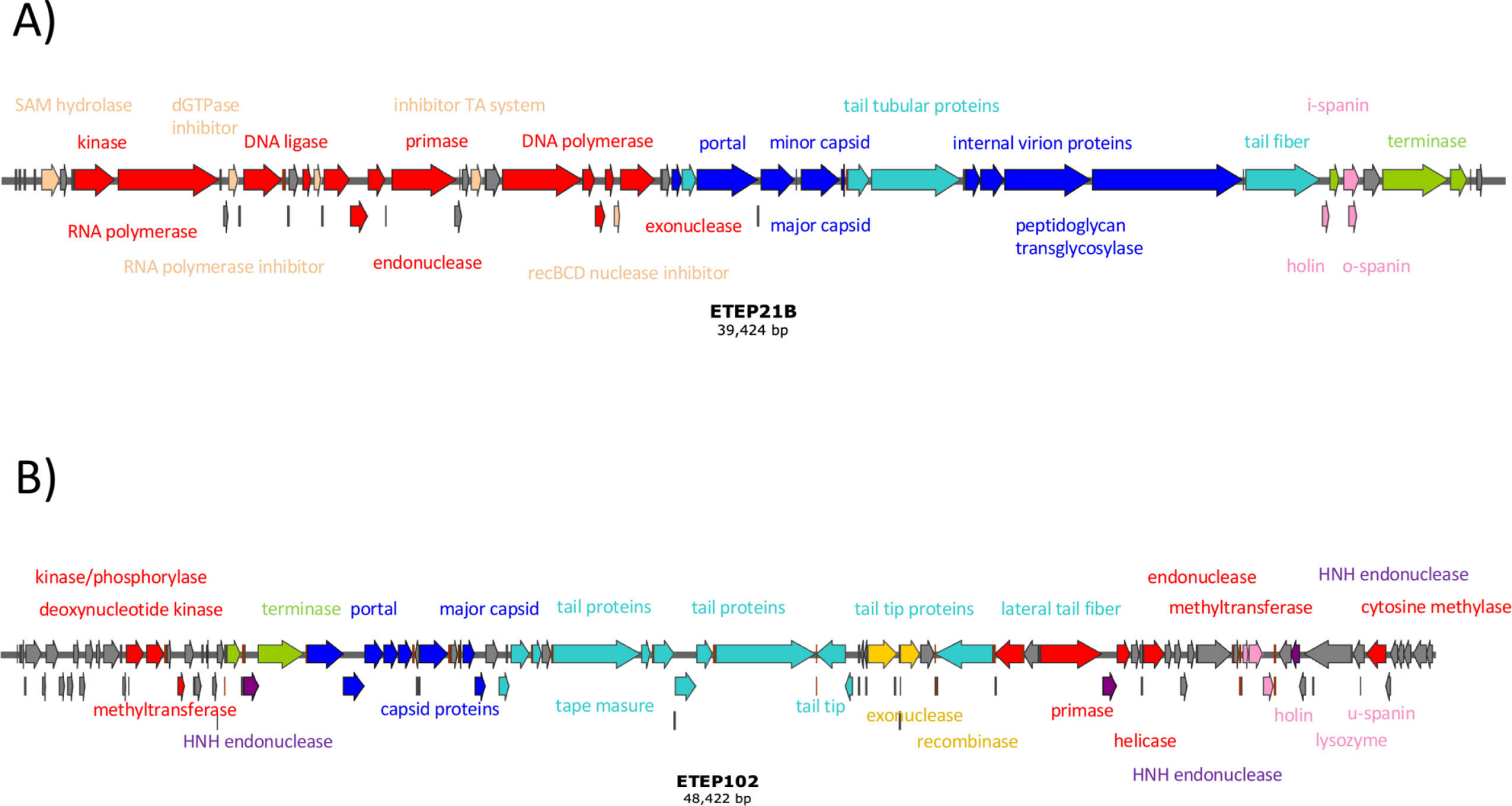
Genomic organization of phages ETEP21B (**A**) and ETEP102 (**B**). Genes are indicated as arrows: capsid morphogenesis in blue, tail morphogenesis in light-blue, DNA packaging in green, lysis-associated genes in pink, genes involved in DNA manipulation in red, morons and homing endonucleases in purple, recombination in yellow, and hypothetical in gray. Putative promoters are indicated as orange lines and terminators as brown lines.

The ETEP21B genome was predicted to encode 46 open-reading frames (ORFs), all but 3 (initiating with GUG) starting with the AUG codon. Few ORFs (10 ORFs, corresponding to 22%) were annotated as hypothetical proteins, with no similarity to characterized proteins (Fig. 5A; supplemental file S6A). ETEP21B had the classic genome structure of the well-described *E. coli* phages T7 and T3 (40), with early (ORFs 01 to 07) and middle genes (ORFs 08 to 24) for DNA metabolism and host takeover and late genes for phage morphogenesis (ORFs 25 to 38) and lysis and packaging (ORFs 39 to 46). Based on the similarities with T7, ETEP21B was expected to initiate transcription using the *E. coli* RNA polymerase via the three strong promoters predicted near the left genome end. Transcription was expected to continue using the ETEP21B-encoded RNA polymerase initiated at phage-specific promoters, predicted at positions homologous to T7 promoters. In addition to two terminators downstream of the DNA ligase (ORF07) and the capsid genes (ORF31) also present in T7, an additional terminator was predicted downstream of the small terminase subunit gene (ORF40), suggesting a more complex regulation of the packaging process (supplemental file S6A). As in T7 and T3, the ETEP21B genome encoded most of the proteins needed for transcription and replication during phage propagation, with the early transcribed genes involved in evading the host defense systems, shutting off the host transcription, and switching to the viral transcription. In addition, sites used for methyltransferase modification were underrepresented in ETEP21B (two Dam sites and no Dcm sites), as well as in its closest similar phages (supplemental file S6B) and other *Autographiviridae* phages (40).

**Fig 6:**
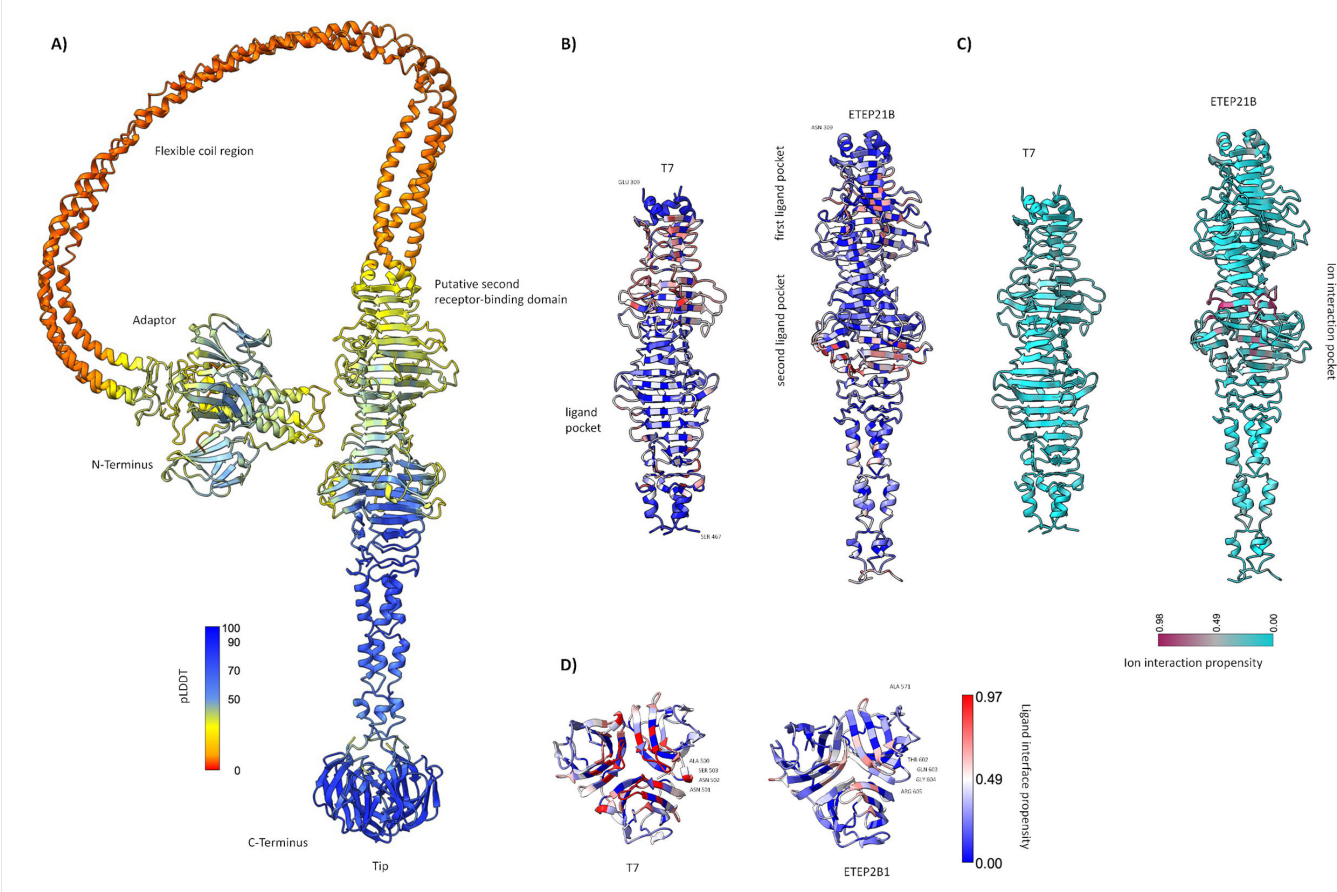
(**A**) AlphaFold2-multimer model of ETEP21B-TF, colored by pLDDT confidence scores. (**B**) Putative second receptor-binding domain in T7-TF and ETEP21B-TF colored by ligand interface propensity and (**C**) ion interaction propensity. (**D**) Tips of T7-TF and ETEP21B-TF colored by ligand interface propensity.

Considering that the O-antigen is known to inhibit the related phage T7 binding to the receptor (40), it was striking that ETEP21B infects many ETEC strains carrying an O-antigen (Fig. 3). Since receptor recognition in T7 relies on the tail fiber, we further compared the tail fiber structures of the two phages. In ETEP21B, ORF38 was predicted to encode for the tail fiber due to its similarity to the T7 tail fiber. We predicted the structure of the ETEP21B tail fiber (ETEP21B-TF) and compared it with an AlphaFold2 prediction of T7 gp17 (T7-TF; supplemental file S7A). Importantly, the T7-TF AlphaFold2 prediction was highly similar to the experimentally determined structures of the C-terminal region (41) and electron micrographs of the complete fiber (42). ETEP21B-TF contains four major structural domains (Fig. 6A): an adaptor region at the N-terminal, a flexible coiled-coil region, a region with a pyramid domain, and a tail tip containing a receptor-binding domain at the C-terminal. Full alignments of T7-TF and ETEP21B-TF resulted in 42.3% overall identity but showed a lower identity in the pyramid domain (only 26.6%) and higher in the tip domain (52%). Compared with T7-TF, the ETEP21B-TF pyramid region exhibited additional interface regions (a complete beta-strand with some affinity for ligand binding and a pocket) with ligands and ions, suggesting it may function as a putative second receptor-binding domain (Fig. 6B and C; supplemental file S7B) and that distinct ligands could be accommodated. Moreover, two additional short alpha-helices connected the pyramid domain to the tip in the C-terminal. The four external loops in the T7-TF tip were known to be responsible for receptor recognition in phage T7 (41). The ETEP21B-TF tip had a similar architecture to the T7-TF tip, except for loops 2, 3, and 4 (Fig. 6D). The reduced ligand interface propensity score of ETEP21B-TF loop 2 (THR602 and ARG605) suggested a reduced interaction with the receptor compared with T7-TF. Instead, loop 3 exhibited a propensity to bind ligands such as sugars, and the residue ARG643 (one residue before loop 4) was predicted to have a high propensity for protein binding. Overall, the ability of ETEP21B to infect strains carrying O-antigens could originate from the differences highlighted in the loops, especially loop 3, and/or in the putative second receptor-binding domain that potentially could host two different receptors.

We also noticed that ETEP21B could infect rough strains (ECOR04, MG1655; supplemental S3), showing that the O-antigen was not essential for phage infection. Thus, ETEP21B infection was tested on a collection of ET54 strains deleted for common coliphage receptors, including some lacking the last sugars of the outer core and the O-antigen (Δ*waaC*) or only the O-antigen (Δ*waaR*). Phage ETEP21B infected all mutants with the same efficiency as the wild type, except for Δ*waaC*, where a 3-log reduction indicated that sugars in the inner core or the first sugars of the outer core were needed for ETEP21B infection but not the O-antigen (supplemental file S6C). Double receptor mutants prepared in the *ΔwaaC* background did not reduce infection further, leaving a potential secondary receptor for ETEP21B unknown. In addition, ETEP21B infected a few strains with different serotypes, namely, ET107 (O141), ET117 (O139), ET119 (O139), and ET159 (O138). The different structures of these four O-antigens [O149 has a linear three-residue backbone, O138 a linear four-residue backbone, O141 a branched three-residue backbone, and O139 a branched six-residue backbone; reviewed in reference (43)] and the ability of ETEP21B to still infect *waaC* mutants suggested that ETEP21B recognized multiple sugars in the core. Alternatively, an unidentified second receptor interacting with the putative second binding pocket highlighted by the ETEP21B-TF structural analysis may play a role in ETEP21B infection.

### The phage ETEP102 tail apparatus suggested a two-step infection process, starting with a depolymerase activity

**Fig 7:**
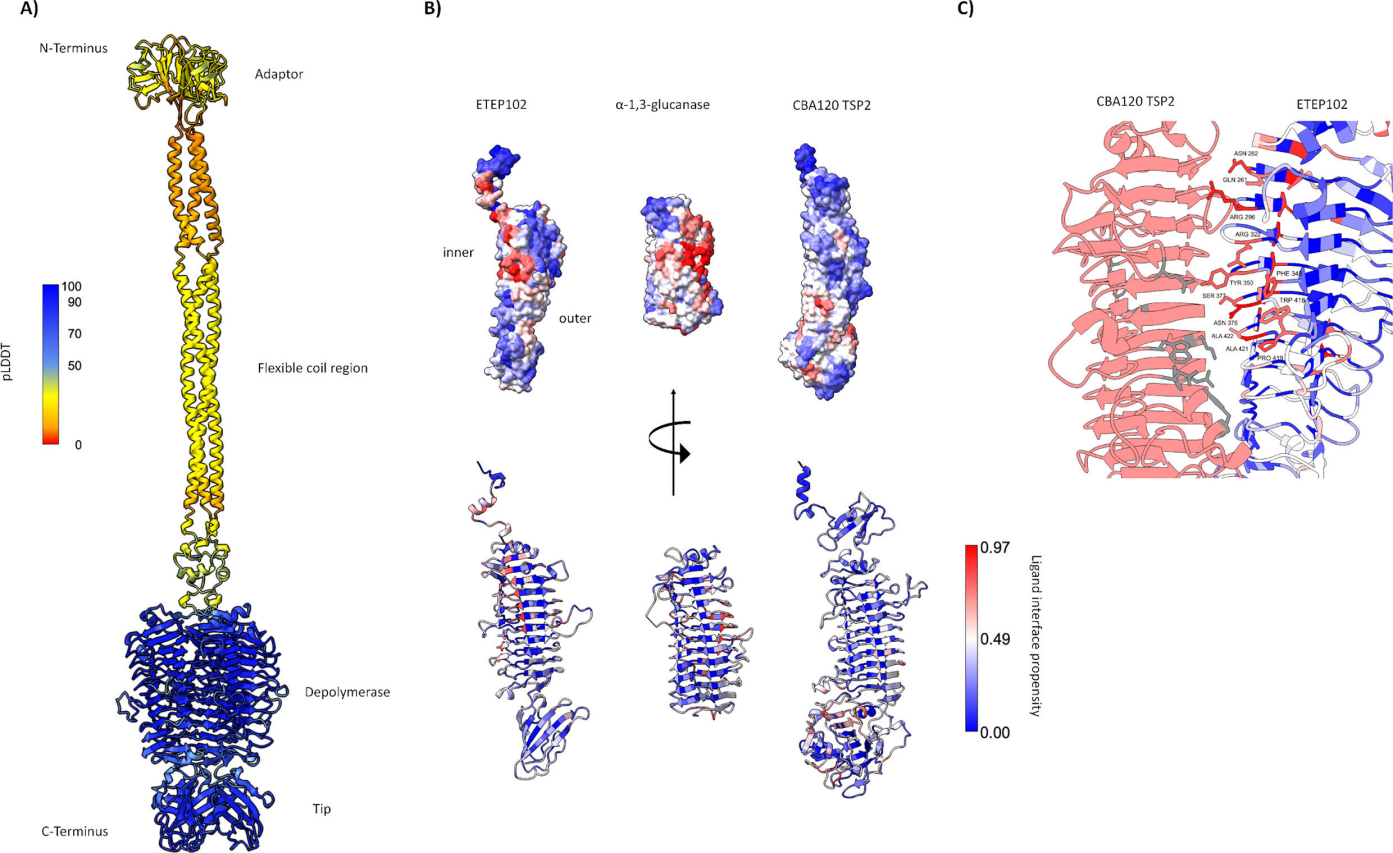
(**A**) AlphaFold2 model for the ETEP102 tail fiber colored by pLDDT confidence scores. (**B**) Comparison of ETEP102 fiber depolymerase with the α-1,3-glucanase of *Paenibacillus* sp. (PDB-ID 6k0n) and CBA120 TSP2 (PDB-ID 5W6P), colored by ligand interface propensity. (**C**) Alignment of ETEP102-TF (right) with CBA120 TSP2 (left), showing that residues with high ligand interface propensity in ETEP102 are located closer to the N-terminus compared with the residues known to bind O-157 in TSP2 (in gray). The root mean square deviation between the two structures is 1.12 angstroms, considering 90 pruned atom pairs.

The ETEP102 genome was predicted to encode 81 ORFs, all but five (initiating with GCG and four with GUG) starting with the AUG codon. Most ORFs (44, corresponding to 55%) were predicted as hypothetical proteins, with no similarity to characterized proteins (Fig. 5B; supplemental file S8), despite the genome synteny with the type coliphage T1 (44) and other well-characterized phages such as coliphage TLS (45) and Rtp (46). Despite the similarity with phages T1 and TLS indicating a similar infection process, more than half of the predicted ORFs had no suggested function and these might largely affect the ETEP102 host range. Our host range analysis (Fig. 3) showed that ETEP102 infects strains with diverse O-antigens, and we thus studied the ETEP102 tail apparatus to understand host recognition. The tail apparatus of ETEP102 was very similar to the *E. coli* phage Rtp, which has leaf-like lateral tail fibers (46), as suggested by the ETEP102 TEM picture (Fig. 4E). In *Drexlerviridae* phages, the lateral tail fibers are thought to reversibly bind O-antigen glycans as primary receptors. The AlphaFold2 model of the ETEP102 tail fiber (ETEP102-TF) exhibits a four-domain structure that includes an adaptor, a flexible coil region, a depolymerase domain, and a tip domain (Fig. 7A). The depolymerase domain was predicted with high confidence, and it shared high structural similarity with glycoside hydrolases produced by *Paenibacillus* sp. [13.9% sequence identity and 164 FoldSeek score with PDB-ID 6KFN (47) and 10.9% sequence identity and 126 FoldSeek score with PDB-ID 6k0n (48)], as well as with the bacteriophage CBA120 tail spike [TSP2 (49); 11.4% sequence identity and 120 FoldSeek score]. Despite their similar architecture, these proteins exhibited distinct ligand propensity regions (Fig. 7B), which suggests a different hydrolase activity of phages ETEP102 and CBA120 and might explain why phage CBA120 did not infect any of the tested ETEC strains (supplemental file S3).

After binding to O-antigen glycans, a second protein receptor was recognized by *Drexlerviridae* for irreversible binding and DNA injection by the tail tip. The ETEP102 tail tip genes (gpJ-like corresponding to ORF45 and following genes) were predicted to be structurally similar to the ones of *E. coli* phage AugustePiccard shown to bind the essential lipopolysaccharide export channel LptD (23). Despite the synteny of the tail tip genes, the relatively low sequence identity between AugustePiccard and ETEP102 genes (55% amino acid identity) left space for uncertainty. In the attempt to experimentally verify the predicted receptor, ET03 spontaneous mutants resistant to ETEP102 were isolated and sequenced. A few mutations with high frequency were identified (equal to 65% or higher; supplemental file S9), but none of them was in *lptD*: a silent mutation in the sugar ABC transporter ATP-binding protein YphE, a missense mutation in a gene expected to encode a D-alanyl-D-alanine carboxypeptidase, involved in the peptidoglycan biosynthesis pathway, and other missense mutations in a gene with high similarity to *traI*, a secreted DNA helicase and relaxase, essential for conjugation (50–52). Deletion mutants of each of these three targets were constructed, but all were infected by ETEP102 at a similar level as the wild type (supplemental file S9), thus leaving LptD as the only predicted ETEP102 secondary receptor.

## DISCUSSION

Porcine ETEC-induced PWD is a severe threat with substantial economic costs and impact on animal welfare. The treatment used so far has relied on the prophylactic use of antibiotics and zinc oxide shown to contribute to the spread of antibiotic resistance (53–55), thus becoming ineffective and unsustainable and, for this reason, banned in the European Union (20). As for many other bacterial infections in humans and animals, no efficient and sustainable solutions are available on the market, and phages have been proposed as a valuable resource. Conscious of the careless use of antibiotics in the past, the European Union expects new interventions to comply with the One Health policy and thus to demonstrate efficacy and safety for humans, animals, and the environment. This requires a detailed knowledge of the pathogen to target and its interaction and response to the intervention.

To obtain detailed knowledge on ETEC causing PWD in piglets, we established and characterized the PHAGEBio ETEC collection. ETEC strains in the collection are available upon request, and the genomes can be found on NCBI for researchers to work on clinically relevant ETEC strains, expanding our understanding of the diversity and evolution of *E. coli*, the most classic yet still poorly understood bacterial species. It has previously been observed that ETEC strains are genetically highly diverse (9, 11). Indeed, the strains in the PHAGEBio ETEC collection fall into the most common phylogroups (A, B1, and D), with mostly generalist and commensal strains. Still, some also belong to the rarest phylogroups (C and E), usually consisting of intestinal and extraintestinal pathogenic strains (56). This genetic diversity mirrors the need and ability for *E. coli* to adapt to multiple and very different niches—from water to mammal gut. As in other *E. coli* strains, this flexibility is supported by high genetic plasticity: niche-specific genes, such as virulence genes that allow animal and human infection that are easily transferred by MGEs, making the *E. coli* species a perfect example of an open pangenome with a high rate of horizontal gene transfer (6, 56, 57). ETEC is no exception, especially since the virulence genes that define the pathotype are often MGE encoded (2), as observed in our collection, particularly for the few strains in phylogroups C and E (supplemental file S2). The genetic diversity is also revealed by the diverse surface, with the 19 different O-antigens identified in our collection that were commonly reported for porcine ETEC strains before (1, 12, 58, 59) and the predominance of the O149 antigen for the F4 strains. At the same time, the O-antigen of F18 varied between O138, O139, O141, O149, and a few others. The extreme diversity of ETEC genomes and O-antigens previously suggested that any *E. coli* may acquire virulence gene-encoding plasmids and become ETEC. Nevertheless, a strong association was found in human ETEC strains between phylogenetic lineages, virulence genes, plasmids, and O-antigens (60), suggesting that specific chromosome traits or lineages might be needed for maintaining plasmids encoding ETEC virulence genes (61, 62). A similar scenario can be imagined for porcine ETEC strains, as we and others [i.e., references (11, 12)] observed a co-occurrence of serotypes, virulence factors, and lineages (e.g., O149:H10, with F4, ST2, and LT in phylogroup A). The *in silico* analysis of our ETEC collection demonstrated that plasmid maintenance is driven by PWD-specific virulence factors (fimbriae and toxins) and resistance to antibiotics and metals. Metals have been reported to co-select for antibiotic resistance genes in several environments and concentrations (63), and, specifically for porcine ETEC (12), antibiotic resistance genes were predicted to co-locate on plasmids with metal resistance genes or virulence factors, thus implying that the extended and indiscriminate use of metals and antibiotics will be detrimental in the long term.

In contrast, the internal antiphage resistance mechanisms were predicted to be chromosome encoded. From the point of view of phage therapy and biocontrol, the observation suggests a reduced risk of co-selection of phage resistance genes with virulence genes and antibiotic or metal resistance genes. From an evolutionary point of view, phage resistance genes in our ETEC collection were not horizontally transferred, as elsewhere reported (64). Still, they coevolved with the bacterial chromosome, with a pace and contribution from gene loss and gain similar to any other chromosomal genes (65). In each ETEC genome, we detected several defense systems. As previously reported for *E. coli* and most bacterial species (66), the RM systems are the most prevalent phage defense mechanisms, followed by the CRISPR-Cas and several abortive infection systems. In our collection, while the CRISPR-Cas type I-E system alone is present in most strains, several RM types complement each other and overlap, especially with the RM type IV, which targets modified DNA contrary to the other RM systems (67). In addition, the presence of AVAST type II, CBASS type II, and *ietAS* in a clade of closely related strains of the phylogroup A is a peculiar observation. The different defense systems might act synergistically to defend the cell from the infection of diverse phages (68), to increase the defense efficiency against one phage (64), or to build several consecutive lines of defense to avoid phage immune evasion (69).

Literature data on phage-based products against ETEC-induced PWD are very fragmented, and only two phages (GJ1 from 24 and 25 and CJ19 from 29) have been demonstrated to be lytic and infecting porcine *E. coli* strains with fimbriae and toxins that define ETEC. Here, we isolated several phages and characterized in detail the two lytic phages, ETEP21B and ETEP102, isolated from pig feces samples. These samples were also used to isolate ETEC strains, supporting the notion that phages infect hosts isolated in the same space and time more efficiently (e.g., 38 , 70, 71). These two phages infected 44% of the PHAGEBio ETEC collection, mainly O149: F4, ST2, and LT strains, but not exclusively. Despite the overlap of host range, the two phages are diverse. ETEP21B was classified as a *Berlinvirus*, a podovirus belonging to the same *Autographiviridae* family as the well-studied coliphages T3 and T7 (40). Thanks to this similarity, a function for most genes could be proposed, including the early genes involved in evading the host defense systems, such as the S-adenosylmethionine hydrolase that overcomes the action of RM type I (72) or the dGTPase inhibitor that might allow ETEP21B to infect ET117, a strain carrying a dGTPase as a phage defense mechanism (supplemental file S2). ETEP102 is a siphovirus that belongs to the *Nouzillyvirus* genus, part of the *Drexlerviridae* family, and despite some similarity with the coliphages T1 and TLS, the genome annotation was more challenging, leaving half of the genes with no predicted function. Further analysis suggested that the phages employ diverse strategies for infection and could thus work synergistically in a phage cocktail. For example, ETEP21B is expected to be independent of the host for DNA replication and transcription, whereas ETEP102 most probably relies on the host machinery since it does not encode an RNA polymerase, similar to phage T1 (73). Notably, as demonstrated by the host range, both phages are partially resistant to RM systems, widespread in the PHAGEBio ETEC collection. Common ways to escape DNA-targeting RM systems are the methylation of the viral DNA and the underrepresentation of restriction sites. Restriction sites are underrepresented in ETEP21B (only two Dam and no Dcm sites), as typical for *Autographiviridae* phages (40). In addition, ETEP102 encodes for its own Dam and Dcm, thus suggesting its DNA is methylated, as in other *Drexlerviridae* phages, such as T1 and TLS (73, 74).

Analyzing the host range, the most evident observation was the infection of many O149 strains by both phages, which suggests a crucial role in receptor recognition and binding. Since it is well established that the presence of the O-antigen inhibits T7 infection (40) and that DNA injection follows the binding of tail fibers to sugars in the inner core of the LPS on rough *E. coli* strains (75), we compared T7’s and ETEP21B’s tail fibers to understand the ETEP21B infection process of O149 strains. The four external loops at the tip of the T7 tail fiber are essential for recognizing the receptor (41). Our data suggest that loop 3 might contribute more significantly to a difference in the host range because of its predicted propensity to bind sugars. Nevertheless, ETEP21B’s TF structure is more complex than T7’s, with a possible second ligand domain that could host an additional or different receptor than T7, contributing to the ability to infect O149 strains. Based on host ranges of mutants lacking known phage receptors, it was established that ETEP21B recognizes sugars in the LPS core as a receptor and that it can still infect the *waaC* mutant at low EOP, as previously observed for most *Autographiviridae* phages (23). To explain this observation, it was previously speculated that these phages might recognize multiple sugar combinations (76) or a second receptor, such as OmpA and OmpF porins (77, 78). For ETEP21B, it was not possible to identify a second receptor, and the infection of additional strains with other O-antigens supports the idea that binding does not depend on a specific O-antigen glycan.

Phages in the *Drexlerviridae* family, such as ETEP102, infect their host via a two-step process with the reversible binding of LPS first by the tail fibers and the recognition of a second receptor by the distal end of the tail tip protein (46, 79). Given the recognition of mainly O149 strains, we suggest that ETEP102 tail fibers can bind and depolymerize glycans of this specific O-antigen. The second and irreversible receptor is usually an outer membrane protein, and it can be predicted by comparison with the different gene variants encoding for the receptor binding proteins (RBP) in the locus downstream of the *gpJ* homolog (23). The RBP locus in ETEP102 encodes proteins that are structurally similar to phage Rtp, which uses the essential LPS export channel LptD as receptor (23). Nevertheless, the low sequence similarity raised some doubts about assigning LptD as receptor of phage ETEP102, thus leaving ETEP102’s second receptor to be confirmed with additional experiments.

### Conclusion

ETEC is a heterogenous pathotype both in the virulence genes encoded and in their phylogeny. Antibiotic resistance genes and pathotype virulence factors are mostly plasmid encoded, while phage resistance genes and other virulence factors are mostly on chromosomes, thus reducing the risk of transfer to other strains and co-selection with virulence genes and antibiotic or metal resistance genes. Because of the high number of encoded resistance mechanisms, ETEC strains seem to be resistant to *Enterobacteriaceae* phages in a complex and multifactorial way. Nevertheless, we could isolate two lytic phages infecting ETEC strains and covering a large part of our diverse ETEC collection. By studying the genome of the two ETEC phages, it was observed that they have different strategies to propagate in the bacterial host and overcome the defense systems. In addition, the receptors for the two phages were identified as part of the LPS and speculated that specific loops and pockets in the tail fiber structures of the two phages allow recognition and binding of ETEC strains despite the presence of O-antigens. With this work, we contribute to the sustainable use of phage products specifically targeting the ETEC strains that are currently causing PWD in Europe, and we hope that the scientific community will use the collection to expand our knowledge from laboratory to clinically relevant *E. coli* strains.

## MATERIALS AND METHODS

### ETEC collection and other bacterial strains

Seventy-nine ETEC isolates were collected by isolating hemolytic *E. coli* strains from pigs with PWD in the period from 2014 to 2019 from different farms in Denmark (via the surveillance institute Danish Pig Research Center, SEGES), Belgium-Netherlands, Greece, and Italy (via the surveillance institute Istituto Zooprofilattico Sperimentale della Lombardia e dell’Emilia Romagna, IZSLER) (supplemental file S1). Fimbriae and toxins were identified at isolation by multiplex quantitative PCR ( qPCR) by SEGES (80) and by multiplex PCR by IZLER (81) and then further confirmed once received at the University of Copenhagen (7).

ETEC strains were streaked on blood agar plates (BA, 5% calf blood in blood agar base, Thermo Fischer, CM0055) to ensure the absence of contaminants, grown in LB (Lysogeny Broth, Merck) at 37°C, with agitation, overnight (ON), and stored at −80°C as frozen stocks in 15% glycerol. Where stated, the strains have been grown in LB with mucin (LB M; 0.1 g/mL porcine mucin, M1778-10G, Sigma-Aldrich) or BHI broth (Brain Heart Infusion broth, CM1135, Oxoid).

The laboratory strains ECOR04, MG1655, and NCTC12900, used for the host range and for propagation of phages not isolated in this work, were grown in LB at 37°C, with agitation, ON, and stored at −80°C as frozen stocks in 15% glycerol.

### DNA extraction and sequencing

Bacterial DNA was extracted from ON cultures of the 79 ETEC strains with DNeasy Blood & Tissue Kit (Qiagen). Except for ET54 that was sequenced with MiSeq (Illumina, San Diego, CA, USA), the remaining strains were sequenced by paired-end libraries with an insert size of 200–400 bp and further sequenced on the MGISEQ-2000 (MGI, BGI-Shenzhen) platform to obtain about >100× clean reads data. Quality control for clean reads was performed by filtering with SOAPnuke and fastp (82), and clean reads were assembled with SPAdes v3.13.0 (83).

### Assembly and genome analysis

All ETEC genomes are available on GenBank (BioProject ID: PRJNA770188) as the PHAGEBio ETEC collection. Sequence quality has been checked by BUSCO. The genomes varied widely in size from 4.9 to 6.2 Mb (average 5.5 Mb), with a density of protein-coding sequences of the range 82%–88% (average 86%) and GC content of the range 50%–50.9%. The pan-genome contains 20,909 gene families, and the core genome contains 2779 genes (13%).

Antibiotic resistance, metal resistance, and virulence factor genes in the bacterial genomes were predicted using tools ABRicate v1.0.0 (https://github.com/tseemann/abricate) and NCBI AMRFinder Plus v3.10.30 (84) using default parameters. The defense systems in these bacterial genomes were identified using the tools PADLOC v1.1.0 (85) and DefenseFinder v2.1.1 (66, 86) using standard parameters. The plasmid-borne contigs (not closed plasmids) were identified using the tool Platon v1.6 (87). The prophage regions were detected using the tool Phigaro v2.3.0 (88). The anti-microbial resistance, virulence, and defense system genes were mapped on plasmid and prophage regions using customized Python scripts (supplemental file S2).

The ETEC phylogroup was established by submitting the ETEC genomes to EzClermont (https://ezclermont.hutton.ac.uk/; 89) and further confirmed by building a phylogenetic tree with the ETEC strains, the 72 strains of the *Escherichia coli* Reference Collection (ECOR; 90) and *Escherichia fergusonii* (NC_011740.1) as an outgroup using Population Partitioning Using Nucleotide K-mers (PopPUNK) v 2.5.0 (91). The core and the accessory genome distance between the strains was estimated for K-mer sizes 15 to 31 with a step size of 2. The phylogeny created by PopPUNK was visualized using Microreact (92) and iTOL v6.7 (93) (supplemental file S2).

### Receptor mutant generation

A series of knockout mutants in genes coding for known or potential phage receptors was generated in ETEC strains ET54 and ET3 using Lambda-Red-mediated mutagenesis. A list of used primers is available as supplemental file S10. Allelic replacement was facilitated by the recombination protein expression plasmid pRedET from the Quick & Easy *E. coli* Gene Deletion Kit (Gene Bridges) and based on the FRT-PGK-gb2-neo-FRT cassette also supplied in the kit. All deletion fragments were synthesized by PCR; the majority were amplified with gene-specific primers on mutants recently generated in *E. coli* ECOR04 (24), while the remaining were made with resistance cassette-specific primers equipped with 50-bp extensions matching the targeted gene. For ET54 double mutants, a *waaC* mutant was first generated with a deletion fragment encoding chloramphenicol resistance, amplified from pKD3, and the secondary mutations were then introduced by the kanamycin resistance-encoding mutations already established in single mutants. Target strains were first transformed with pRedET by heat shock using a standard CaCl_2_ chemically competent cell protocol and selected on 100 mg/L ampicillin at 30°C. Transformants were subsequently grown in liquid culture (LB, 100 mg/L ampicillin, 30°C, 180 rpm) until an optical density at 600 nm (OD600) of 0.3, followed by induction of plasmid expression with 0.35% L-arabinose (final concentration) for 1 hour at 37°C. Cells were washed twice with one volume, once with 1/2 volume, and once with 1/4 volume and finally resuspended in 1/100 vol of ice-cold water. Fifty microliters of competent cells was mixed with 2 µL concentrated, PCR-generated deletion fragment in a pre-chilled electroporation cuvette and pulsed in a MicroPulser Electroporator (Bio-Rad) employing the Ec2 setting. Transformed cells were recovered in 1 mL LB and incubated at 37°C for 2–3 hours before selection on LA with 15 mg/L kanamycin or 25 mg/L chloramphenicol at 37°C ON. Target gene knockout was confirmed with gene-specific primers.

### Sample processing and phage isolation

Wastewater (*n* = 3), pig manure (*n* = 4), gut samples from pigs dying from ETEC diarrhea (*n* = 23), feces from healthy pigs (*n* = 16), and feces from ETEC-affected pigs (*n* = 31) have been processed and screened for phages infecting ETEC. Solid samples were diluted (1:10 wt/vol) in sterile SM buffer (0.1 M NaCl, 8 mM MgSO_4_*7H_2_O, 0.01% gelatine, 50 mM Tris-HCl, pH 7.5), stomached, and centrifuged (18,000 × *g* for 10 min). Supernatants were filtered twice through 0.45µm filters and stored at 4°C.

The 77 supernatants were screened for phages infecting ETEC by direct spotting and enrichment on the 41 Danish ETEC strains (from ET03 to ET54). For the enrichments, 400 µL of each supernatant was added to 1 mL of liquid medium (LB, LB + M, BHI) and 400 µL of a 4-hour culture of each of the selected 41 ETEC strains and incubated ON at 37°C in static or agitation. The day after the enrichments were centrifuged for 5 min at 10,000 × *g*, and three drops of 10 µL each of the enriched and not enriched supernatants were spotted on bacterial lawns of the respective ETEC strains, along with the not-enriched supernatants. For preparing bacterial lawns, 330 µL of a 4-hour cultures (LB, agitation, 37°C) was mixed with 11 mL of molten overlay agar (LBov: LB with 0.6% Agar bacteriological no.1, Oxoid; BHIov: BHI with 0.5% Agar bacteriological no.1, Oxoid) and spread on square 12-cm plates (LA: LB with 1.2% agar, or BHIA: BHI with 1.2% agar) plates. After settling for 5 min, lawns were dried in a laminar hood for 35 min and used immediately thereafter. For any spot with an inhibition halo, the spotted supernatant (enriched or not) was 10-fold diluted in SM buffer and spotted on a lawn of the same ETEC strain in order to observe plaques. Visible plaques were transferred to 500 µL of SM buffer (0.1 M NaCl, 8 mM MgSO_4_*7H_2_O, 50 mM Tris-HCl, pH 7.5), vortexed, and 10-fold diluted. A 100-µL aliquot of selected dilutions was mixed with 100 µL of the isolation strain in 3.2 mL LBov or BHIov and spread on LA or BHIA plates. Each single plaque was purified by repeating this procedure for at least three rounds. Single plaques from the final purification steps were used for phage propagation on the isolation strain, and phage stocks were prepared by the plate lysis methods as adopted from Carlson (94).

### Phage host range

The host range of the isolated ETEC infecting phages (ETEP21B, ETEP102), of 42 *Salmonella* phages (38, 39) and up to 200 *E. coli* phages, including the T3, T7, and CBA120, and others isolated on *E. coli* MG1655 and ECOR04 have been verified by spotting assay. Tenfold serial dilutions (up to 10^−7^−10^−9^) of the stocks in SM buffer were prepared, and 10-µL aliquots were spotted on bacterial lawns of the ETEC strains, let adsorb, and incubated ON. The day after, plaques were counted for each phage and strain and plaque-forming units per mL (PFU/mL) and the efficiency of plating compared with the propagation host (EOP) were calculated. Log of the calculated efficiency of plating is reported in supplemental file S3. At least two independent replicates confirmed the host range.

### Phage DNA extraction, sequencing, assembly, and annotation

DNA was extracted from 10^9^ PFU/mL stocks of phages ETEP21B and ETEP102 with a modified phenol-chloroform protocol (95). Briefly, phage stocks were exposed to RNAse (10 µg/mL, Thermo Fisher Scientific, Waltham, MA, USA) and DNAse (20 µg/mL, Thermo Fisher Scientific) activity at 37°C for 3 hours in a thermo-shaker (500 rpm, Eppendorf, Germany). Following the addition of EDTA (20 mM) and proteinase K (50 µg/mL, Thermo Fisher Scientific) and incubation at 56°C for 4 hours, phenol (Fluka), phenol-chloroform-isoamyl alcohol (25:24:1, Ambion), and three rounds of chloroform-isoamyl alcohol (24:1) treatment were performed. To precipitate the DNA, 0.1 vol of 3 M sodium acetate (pH 5.5), glycogen (final concentration of 0.05 µg/µL, Thermo Fisher Scientific), and 2.5 volumes of ice-cold ethanol (99.9%) were added. After incubation at −20°C for 72 hours, precipitated DNA was centrifuged at 31,000 × *g* for 20 min, washed three times with 70% ice-cold ethanol, and dissolved in 10 mM Tris-HCl (pH 8.0). DNA concentration was measured using Qubit (Thermo Fisher Scientific). DNA libraries were prepared using the Nextera XT v.3 (Illumina, San Diego, CA, USA) Kit. Next-generation sequencing was performed using the MiSeq (Illumina) platform with the paired-end (2 × 250 bp) operating mode.

Sequencing reads were *de novo* assembled using CLC Genomics Workbench 9.5.3 (Qiagen, Aarhus, Denmark). A consensus sequence was obtained with a minimum of 30-fold coverage. Analysis and annotation of the phage genome were performed using tools in Galaxy (cpt.tamu.edu/galaxy -pub) and Web Apollo, hosted by the Center for Phage Technology at Texas A&M University (CPT Galaxy) (https://cpt.tamu.edu/galaxy-pub/). Gene calling was performed using GLIMMER 3.0 and MetaGeneAnnotator 1.0 within the structural workflow, while for the genome annotation, the functional workflow was used, by interrogating the databases of UniProtKB Swiss-Prot/TrEMBL, Canonical Phages, and HHMER. The promoters were identified with PhagePromoter (96), selecting *E. coli* as host, and manually curated. Terminators were predicted with TrasnTermHP, available on CPT Phage Galaxy, and manually curated (97). Putative spanins have been identified with the spanin tools in CPT Phage Galaxy and curated as suggested by Kongari (98). Phage genomes were visualized with the SnapGene software (www.snapgene.com). The presence of encoded tRNA genes in the phage was checked using Aragorn (99), and the phage codon usage was established with the Sequence Manipulation Suite (100). In addition, homolog detection and structural prediction by HHPRED (101), SWISS-MODEL (102), and, to some extent, PHYRE2 (103) were used to investigate further the function of the predicted structural proteins in the two phages.

### Phage taxonomy and genome comparison

To taxonomically classify ETEP21B and ETEP102, VIRIDIC (104) and vConTACT v2.0 0.9.19 (105) were run to identify the genus cluster the two ETEC phages belong to. The two ETEC phage genomes and other 158 genomes (their closest relatives, one genome for each of the genera within the same family, and one genome for each of the other families as listed in the ICTV_Master_Species_List_2021_v2.xlsx) were used to build a phylogenetic tree with VIRIDIC for final taxonomic classification. ANI has been calculated with an ANI calculator on EZBioCloud (www.ezbiocloud.net). Phage genome alignment with others within their genera was run and visualized with Clinker (106).

### Transmission electron micrographs

For visualization of phage ETEP21B and ETEP102, samples were prepared as previously described (107). Briefly, high-titer phage stocks were pelleted at 12,000 × *g* for 60 min, at 4°C, and then washed three times with ammonium acetate (0.1 M, pH 7). For imaging, 200 mesh copper-coated grids (Ted Palla Inc.) were glow discharged using a Leica Coater ACE 200 for 30 sec at 10 mA. Grids were incubated with phage samples for 30 sec at room temperature. Liquid in excess was blotted off, and grids were stained with 2% uranyl acetate for 30 sec. Imaging was performed using a CM100 electron microscope with a Bio TWIN objective lens and a LaB6 emitter. Images were taken using an Olympus Veleta camera. Phage morphology features were measured using ImageJ.

### AlphaFold2 prediction and structure analysis

The tail fiber models for ETEP21B, ETEP102, and T7 were predicted using AlphaFold2-multimer v3.2.1 (108, 109) on an Nvidia Quadro RTX 8000 GPU. The full database search was employed for the multiple sequence alignment. The multimer mode utilized two seeds per model, while the side chain relaxation step was omitted, with a maximum of five recycles. The best model for each fiber was selected based on the ipTM+ pLDDT scores (Predicted Aligned Error Matrices in supplemental file S7C). Structural homologs were identified using the online version of FoldSeek (110).

For structure analysis, we utilized ChimeraX-v1.5 software (111) for tasks such as protein superposition, model handling, and coloring. Protein interaction interfaces were predicted using the online version of PeSTo (112). The protein topology diagram was generated using the online tool PDBSum (113).

## Data Availability

ETEC genomes have been deposited in the GenBank database with the BioProject ID PRJNA770188 and the phage genome sequences under accession numbers OR979722 (ETEP21B) and OR979723 (ETEP102). ETEC strains are available upon request to Lone Brøndsted, Department of Veterinary and Animal Sciences, University of Copenhagen, Stigbøjlen 4, 1870, Frederiksberg C, Denmark.

## SUPPLEMENTAL MATERIAL

Supplemental material part I aem.00807-24-s0001.pdf Files S1 to S5.

Supplemental material part II aem.00807-24-s0002.pdf Files S6 to S10.

ASM does not own the copyrights to Supplemental Material that may be linked to, or accessed through, an article. The authors have granted ASM a non-exclusive, world-wide license to publish the Supplemental Material files. Please contact the corresponding author directly for reuse.
