# Supplementary material for: Diversity and phage sensitivity to phages of porcine enterotoxigenic Escherichia coli
Source: Appl Environ Microbiol. 2024 Jun 28;90(7):e00807-24. doi: 10.1128/aem.00807-24 (PMC11267873; doi:10.1128/aem.00807-24)
Supplement: Supplemental material part I — Files S1 to S5. [file aem.00807-24-s0001.pdf]

SUPPLEMENTAL FILES 1-5

SUPPL FILE S1. The ETEC collection. A) For each strain we report: the identification number (ETEC ID), the type of sample (sample type), the year and the country (country; DK= Denmark, ITA= Italy, BNL=Belgium-Netherlands, GR=Greece) it has been isolated from; the fimbriae and the toxins identified by multiplex PCR; the serotype and the phylogroup, respectively predicted with SerotypeFinder 2.0 (cge.food.dtu.dk/services/SerotypeFinder/) and the In Silico Clermont Phylotyper (ezclermont.hutton.ac.uk), the number of predicted plasmid contigs and of prophages. B) phylogenetic tree built with the ETEC and the ECOR collection, representing the diversity of *E. coli*, to confirm the predicted phylogroups the ETEC strains fall into. C) Average number of contigs from plasmids (pl) and prophages (pro) and of hits in each phylogroup (A, B1, B2, C, D, E): metal, antibiotic resistance (AMR), virulence factors (vir), defense mechanisms (def), on chromosome (ch), plasmids (pl) or prophages (pro). On average, the phylogroups C and E had a higher number of MGE-related contigs (supplemental file S1 C to G) than the other phylogroups.

A)

ETEC ID	sample type	year	country	fimbriae	toxins	serotype	phylogroup	contigs plasmids	contigs prophages
**3**	gut	2018	DK	F4	ST1, ST2, LT	O149:H10	A	33	7
**4**	gut	2018	DK	F4	ST2, LT	O149:H10	A	48	7
**6**	gut	2018	DK	F4	ST1, ST2, LT	O6:H16	A	45	6
**8**	gut	2018	DK	F4	ST2, LT	O149:H10	A	31	6
**10**	gut	2018	DK	F4	ST2, LT	O149:H10	A	41	12
**11**	gut	2018	DK	F4	ST2, LT	O149:H10	A	63	6
**12**	gut	2016	DK	F4	ST1, ST2, LT	O149:H10	A	44	12
**14**	gut	2016	DK	F4	ST2, LT	O149:H10	A	28	7
**15**	gut	2016	DK	F4	ST2, LT	O149:H10	A	46	10
**16**	gut	2015	DK	F4	ST2, LT	O149:H10	A	53	9
**17**	gut	2015	DK	F18	ND	O139:H1	D	26	4
**18**	gut	2015	DK	F4	ND	O149:H10	A	48	6
**19**	gut	2015	DK	F4	ST2, LT	O149:H10	A	38	5
**20**	gut	2015	DK	F4	ST1, ST2, LT	O149:H10	A	40	7
**21**	rectal swab	2015	DK	F18	ND	O138:H14	D	82	6
**22**	faeces	2015	DK	F4	ST2, LT	O149:H10	A	59	5
**23**	gut	2018	DK	F4	ST2, LT	O149/O6:H10/H16	A	47	6
**24**	gut	2018	DK	F4	ST2, LT	O6:H16	A	67	10
**25**	gut	2018	DK	F4	ST2, LT	O149:H10	A	39	10
**26**	faeces	2018	DK	F18	ST2, LT	O149:H10	A	27	5
**27**	gut	2018	DK	F4	ST2, LT	O149:H10	A	57	12
**29**	faeces	2018	DK	F18	ST1, ST2	O138:H14	D	43	6
**30**	gut	2018	DK	F4	ST2, LT	O149:H10	A	33	13
**33**	gut	2018	DK	F18	LT	O147:H14	D	27	4
**34**	rectal swab	2018	DK	F4	ST2, LT	O149:H10	A	29	3
**35**	gut	2018	DK	F4	ST2, LT	O149:H10	A	40	7
**36**	rectal swab	2018	DK	F18	ND	O141ab/ac:H4	A	46	8
**37**	gut	2018	DK	F4	ST2, LT	O149:H10	A	39	5
**38**	gut	2018	DK	F18	ST1, ST2, LT	O8:H23	B1	45	11
**39**	gut	2018	DK	F4	ST2, LT	O149:H10	A	50	2
**40**	gut	2019	DK	F18	ST1	O182:H14	D	64	5
**41**	gut	2019	DK	F4	ST2, LT	O149:H10	A	30	4
**43**	rectal swab	2019	DK	F18	ST2, LT	O51:H42	D	23	6
**44**	rectal swab	2019	DK	F4	ST2, LT	O149:H10	A	35	3
**45**	gut	2019	DK	F18	ST2, LT	O149:H10	C	37	2
**46**	rectal swab	2019	DK	F4	ST1, ST2, LT	O149:H10	A	19	8
**47**	faeces	2019	DK	F18	ND	O149:H19	C	18	3
**48**	faeces	2019	DK	F18	ST1	O108:H19	B1	27	10
**49**	gut	2019	DK	F18	ST2, LT	O138:H14	D	15	4
**50**	gut	2019	DK	F4	ST2, LT	O149:H10	A	40	6
**54**	gut	2019	DK	F4	ST2, LT	O149:H10	A	44	12
**102**	rectal swab	2018	BNL	F4	ST1, ST2	O23:H47	A	40	9
**106**	rectal swab	2018	BNL	F4	ST1, ST2	O23:H37	A	63	4
**107**	rectal swab	2018	BNL	F18	ND	O141ab/ac:H4	A	40	10
**108**	rectal swab	2018	GR	F4	ST1, ST2	O8:H23	B1	26	6
**115**	rectal swab	2018	BNL	F4	ST1, ST2	O23:H37	A	47	15
**116**	rectal swab	2018	BNL	F4	ST1, ST2	O23:H37	A	31	9
**117**	rectal swab	2018	BNL	F18	ND	O139:H1	D	43	7
**118**	rectal swab	2018	BNL	F6	ST2	O35:H6	A	61	9
**120**	gut	2018	ITA	F4	LT, ST1, ST2	O7:H15	D	39	6
**123**	gut	2018	ITA	F4	ST1, ST2	O89/O162:H10	A	35	12
**125**	gut	2014	ITA	F18	ST1	O131:H4	A	42	5
**126**	gut	2014	ITA	F5/F41	ST1	O9/O89/O162:H9	A	45	13
**127**	gut	2014	ITA	F18	ST1, ST2, LT	O138:H14	D	33	5
**129**	gut	2015	ITA	F4	ST1, ST2, LT	O149:H10	A	44	10
**130**	gut	2015	ITA	F18	ST1	O131:H4	A	38	5
**131**	gut	2015	ITA	F4	ST1, ST2, LT	O149:H10	A	56	6
**133**	gut	2015	ITA	F18	ST1	O98:H4	A	46	5
**134**	gut	2015	ITA	F18	ST1, ST2	O141ab/ac:H4	A	30	6
**135**	gut	2015	ITA	F5/F41	ST1	O9:H18	B1	21	4
**136**	gut	2018	ITA	F18	ST1	O138:H14	D	69	10
**137**	rectal swab	2018	BNL	F4	ST2	?:H20	E	26	5
**138**	rectal swab	2018	BNL	F18	ST1, ST2	O51:H42	D	64	16
**139**	gut	2018	ITA	F4	ST2, LT	O157:H39	A	34	4
**140**	rectal swab	2018	BNL	F4	ST1, ST2, LT	O149:H10	A	36	5
**141**	faeces	2018	ITA	F18	ST1, ST2	O98:H4	A	25	5
**142**	rectal swab	2018	BNL	F18	ST1, ST2	O141ab/ac:H4	A	38	6
**143**	rectal swab	2018	ITA	F18	ST1, ST2	O98:H4	A	45	10
**145**	rectal swab	2018	BNL	F18	ST1, ST2, LT	O76/O8:H19	C	22	6
**146**	gut	2018	ITA	F4	ST1, ST2, LT	O149:H10	A	51	12
**148**	rectal swab	2018	BNL	F18	LT, ST2	O182:H4	A	40	6
**150**	rectal swab	2018	BNL	F4	ST1, ST2	?:H6	A	25	7
**152**	rectal swab	2018	BNL	F4	ST1, ST2, LT	O149:H10	A	19	9
**154**	rectal swab	2018	BNL	F4	ST1, ST2	O23:H37	A	49	8
**155**	rectal swab	2018	BNL	F4	ST2	O98:H5	F	50	4
**158**	rectal swab	2018	BNL	F18	ST1, ST2	O166:H15	D	28	7
**159**	rectal swab	2018	BNL	F4	ST2, LT	O138:H10	A	64	9
**160**	rectal swab	2018	BNL	F4	ST1, ST2	O23:H37	A	60	11
**161**	rectal swab	2018	BNL	F5	ST1, ST2	O149:H9	C	40	6

B)

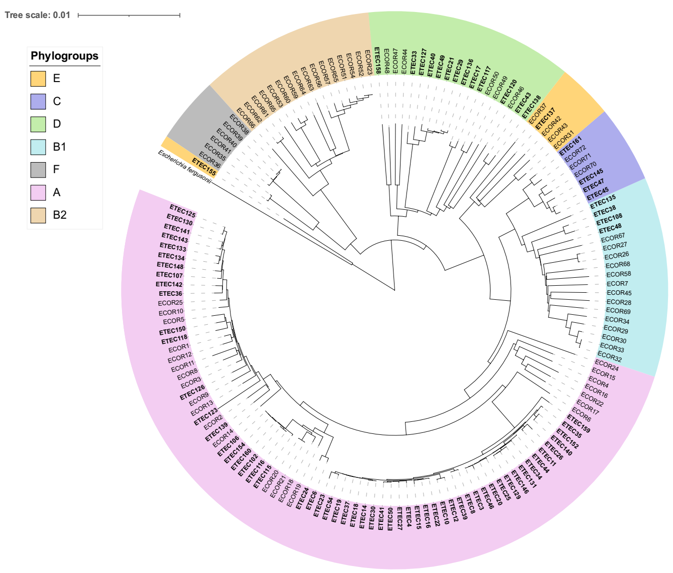

C)

phylogroup	contigs pl	contigs pro	metal ch	metal pl	metal pro	AMR ch	AMR pl	AMR pro	vir ch	vir pl	vir pro	def ch	def pl	def pro
A	40.95	7.23	1.91	1.38	0.14	4.02	3.77	0.00	7.82	4.89	0.13	5.13	0.88	0.02
B1	38.25	5.25	1.50	1.75	0.00	3.25	6.75	0.00	8.00	4.75	0.00	4.50	0.75	0.50
C	48.25	8.75	1.00	1.50	0.00	4.75	4.50	0.00	10.75	4.50	0.75	5.25	1.00	0.00
D	37.85	7.23	1.77	2.23	0.08	5.00	5.77	0.00	12.15	4.54	0.23	6.31	0.85	0.00
E	50.00	8.50	0.50	2.00	0.00	2.50	6.50	0.00	9.50	3.00	0.00	6.50	0.50	0.00

SUPPLEMENTAL FILE S2. *In silico* characterization of the ETEC collection. From left to right, phylogenetic tree with phylogroups, O-type, country, fimbriae, toxins, Sensitivity to phages (ETEP21B, ETEP102 and T7), predicted virulence factors, metal and antibiotic resistance, phage defense mechanisms.

Metal resistance was widespread in the collection, with most strains having efflux pumps chromosome or prophage-encoded, and resistance to tellurium mostly chromosome-encoded. Resistance to silver, quaternary ammonium, mercury, and copper was instead mostly plasmid-encoded and more represented in D and E than in other phylogroups. We could detect resistance to arsenic only in ET158, chromosome-encoded.

Antibiotic resistance was also widespread, with 1 to 16 genes for each isolate, predicted to confer resistance to 17 different classes of antibiotics. The most widespread were genes encoding for resistance to aminoglycoside (including the subclasses of apramycin, gentamicin, tobramycin, kanamycin, hygromycin, streptomycin), but also encoding for efflux pumps and beta-lactam (including the subclasses beta-lactam and cephalosporin). Except for some resistance genes that were chromosome-encoded (efflux, fosfomycin, and fosmidomycin), most resistance genes were predicted to be plasmid-encoded. While chromosome-encoded antibiotic resistance per strain was numerically similar among the different phylogroups, plasmid-encoded resistance was on average lower in phylogroups A and C than in the others.

Most genes coding for virulence factors characterizing ETEC (*fedA*/*fedF* and *fim41a* respectively for fimbriae F18 and F41; *sta1*, *stb* and *ltcA* for toxins) were plasmid-encoded, except the ones for the F4 (*K88ab*) and F5 fimbriae (*fanA*), chromosome-encoded. Several other virulence factors were *in silico* predicted in the collection. Most of them could be both chromosome- and plasmid-encoded, and they included adhesins (*air*, *eilA*, *afaA*, *afaB*, *hra*, *iha*, *lpfA*, *papA_F19*, *papC*), iron uptake (*fyuA*, *irp2*, *iroN*, *chuA*, *sitA*), protectins and serum resistance (*traT*, *kpsE*, *neuC*, *kpsMII*, *kpsMII_K5*, *kpsMII_K96*, *ompT*, *terC*), carbohydrate metabolism (*capU*, *gad*), extracellular secretion of proteins (*espD*, *espP*, *sepA*), and other toxins (*astA*, *cba*, *cea*, *celB*, *cia*, *cib*, *cma*, *ehxA*, *hlyA*, *hlyE*, *mchB*, *mchC*, *mchF*, *mcmA*, *usp*) (35–37). The only gene predicted to be prophage-encoded (in 13 of 29 strains encoding for it) was *iss*, which increases survival to serum and protects against phagocytosis (38). Plasmid-encoded virulence genes, though less represented than chromosome-encoded ones, were equally represented among the phylogroups. Chromosome-encoded virulence genes instead were slightly more represented in the phylogroups C, D and E than in the phylogroups A and B1.

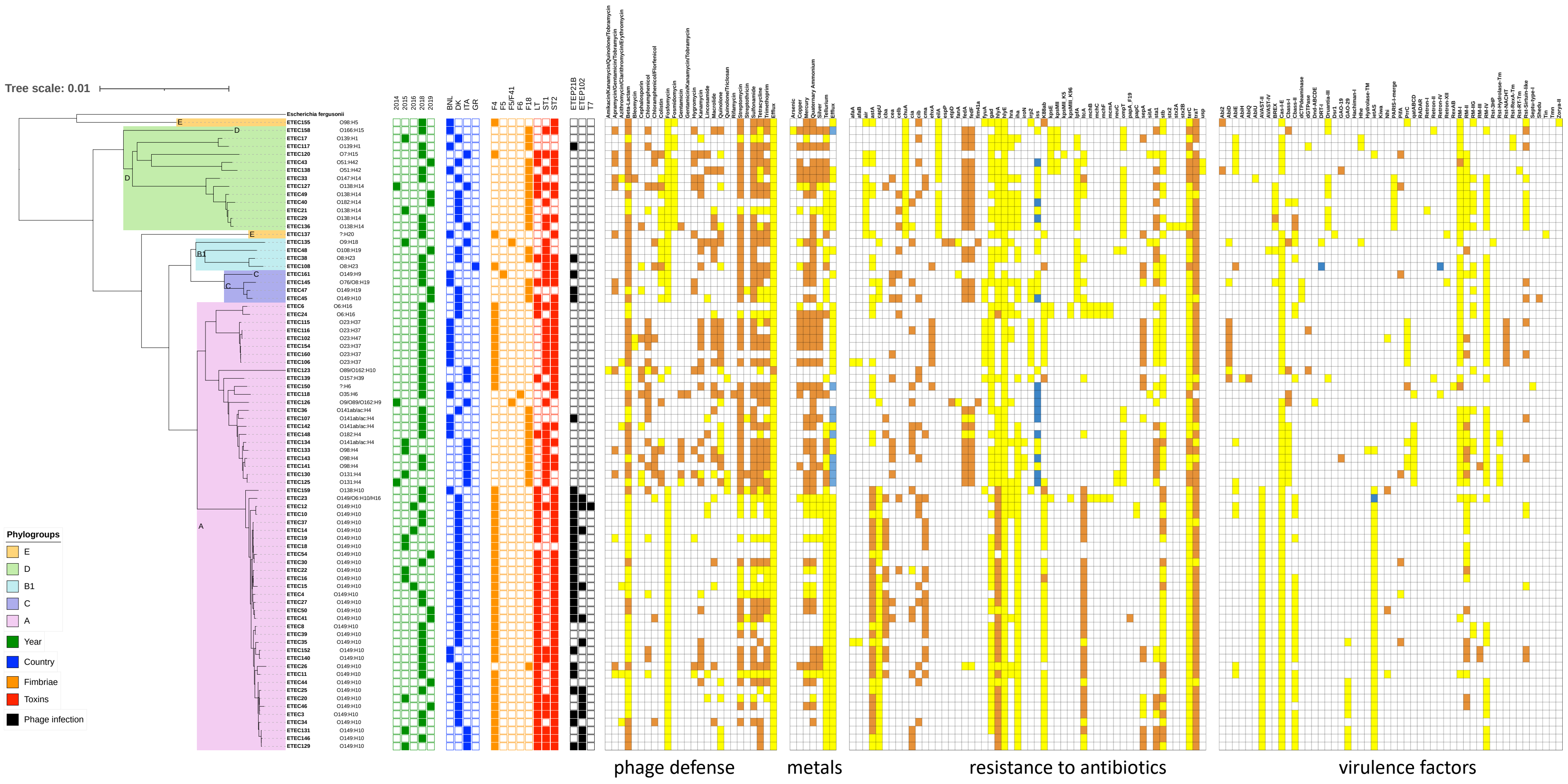
Tree scale: 0.01
Phylogroups
E
D
B1
C
A
Year
Country
Fimbriae
Toxins
Phage infection
phage defense
metals
resistance to antibiotics
virulence factors

SUPPLEMENTAL FILE S3. Host range analysis of tested phages (ETEP21B, ETP102, 42 *Salmonella* phages and up to two-hundred *E. coli* phages, including T3, T7 and CBA120, and others isolated on *E. coli* MG1655 and ECOR04) on the ETEC strains in our collection, along with the phage propagation strains as control. Fimbriae, toxins and serotypes are indicated for each ETEC strain and strains with O149 O-type are highlighted in yellow. The log of the efficiency of plating is reported and colour coded from white (EOP=0; no infection) to green (EOP<1, less infectious than on the propagation strain), dark green (EOP=1, as infectious as on the propagation strain; EOP>1, more infectious than on the propagation strain). *E. coli* and *Salmonella* phages infection of ECOR04 and MG1655 depends on the phage (*).

bacterial strains (rows: **phages**)

ID	propagation strain	ET3	ET4	ET6	ET8	ET10	ET11	ET12	ET14	ET15	ET16	ET17	ET18	ET19	ET20	ET21	ET22	ET23	ET24	ET25	ET26	ET27
Fimbriae		F4	F4	F4	F4	F4	F4	F4	F4	F4	F4	F18	F4	F4	F4	F18	F4	F4	F4	F4	F18	F4
Toxins		ST1, ST2, LT	ST2, LT	ST1, ST2, LT	ST2, LT	ST2, LT	ST2, LT	ST1, ST2, LT	ST2, LT	ST2, LT	ST2, LT	ND	ND	ST2, LT	ST1, ST2, LT	ND	ST2, LT	ST2, LT	ST2, LT	ST2, LT	ST2, LT	ST2, LT
Serotype		O149:H10	O149:H10	O6:H16	O149:H10	O149:H10	O149:H10	O149:H10	O149:H10	O149:H10	O149:H10	O139:H1	O149:H10	O149:H10	O149:H10	O138:H14	O149:H10	O149/O6: H10/H16	O6:H16	O149:H10	O149:H10	O149:H10
**ETEP21B**	1.00	0.80	1.00	0.00	0.00	1.11	1.04	0.97	1.00	1.08	1.07	0.00	1.16	1.15	0.00	0.00	1.29	1.09	0.00	0.85	0.80	1.18
**ETEP102**	1.00	1.00	0.00	0.00	0.00	0.00	0.00	0.68	0.68	0.67	0.00	0.00	0.00	0.00	0.65	0.00	0.00	0.58	0.00	0.98	0.00	0.00
**CBA120**	1.00	0.00	0.00	0.00	0.00	0.00	0.00	0.00	0.00	0.00	0.00	0.00	0.00	0.00	0.00	0.00	0.00	0.00	0.00	0.00	0.00	0.00
**T3**	1.00	0.00	0.00	0.00	0.00	0.00	0.00	0.00	0.00	0.00	0.00	0.00	0.00	0.00	0.00	0.00	0.00	0.00	0.00	0.00	0.00	0.00
**T7**	1.00	0.00	0.00	0.00	0.00	0.00	0.00	0.66	0.00	0.00	0.00	0.00	0.00	0.00	0.00	0.00	0.00	0.00	0.00	0.00	0.00	0.00
**Salmonella phages**	1.00	0.00	0.00	0.00	0.00	0.00	0.00	0.00	0.00	0.00	0.00	0.00	0.00	0.00	0.00	0.00	0.00	0.00	0.00	0.00	0.00	0.00
**E. coli phages**	1.00	0.00	0.00	0.00	0.00	0.00	0.00	0.00	0.00	0.00	0.00	0.00	0.00	0.00	0.00	0.00	0.00	0.00	0.00	0.00	0.00	0.00

		ET29	ET30	ET33	ET34	ET35	ET36	ET37	ET38	ET39	ET40	ET42	ET43	ET44	ET45	ET46	ET47	ET48	ET49	ET50	ET54	ET102
Fimbriae		F18	F4	F18	F4	F4	F18	F4	F18	F4	F18	F4	F18	F4	F18	F4	F18	F18	F18	F4	F4	F18
Toxins		ST1, ST2	ST2, LT	LT	ST2, LT	ST2, LT	ND	ST2, LT	ST1, ST2, LT	ST2, LT	ST1	ST2, LT	ST2, LT	ST2, LT	ST2, LT	ST1,ST2,LT	ND	ST1	ST2, LT	ST2, LT	ST2, LT	ST1, ST2
Serotype		O138:H14	O149:H10	O147:H14	O149:H10	O149:H10	O141ab/ac:H4	O149:H10	O8:H23	O149:H10	O182:H14	O149:H10	O51:H42	O149:H10	O149:H10	O149:H10	O149:H19	O108:H19	O138:H14	O149:H10	O149:H10	O23:H47
**ETEP21B**	1.00	0.00	1.00	0.00	0.89	0.00	0.00	1.02	1.11	0.00	0.00	0.89	0.00	0.00	0.85	0.00	0.95	0.00	0.00	1.06	1.07	0.00
**ETEP102**	1.00	0.00	0.00	0.00	0.00	0.53	0.00	0.00	0.00	0.00	0.00	0.00	0.00	0.00	0.00	0.74	0.00	0.00	0.00	0.00	0.00	0.00
**CBA120**	1.00	0.00	0.00	0.00	0.00	0.00	0.00	0.00	0.00	0.00	0.00	0.00	0.00	0.00	0.00	0.00	0.00	0.00	0.00	0.00	0.00	0.00
**T3**	1.00	0.00	0.00	0.00	0.00	0.00	0.00	0.00	0.00	0.00	0.00	0.00	0.00	0.00	0.00	0.00	0.00	0.00	0.00	0.00	0.00	0.00
**T7**	1.00	0.00	0.00	0.00	0.00	0.00	0.00	0.00	0.00	0.00	0.00	0.00	0.00	0.00	0.00	0.00	0.00	0.00	0.00	0.00	0.00	0.00
**Salmonella phages**	1.00	0.00	0.00	0.00	0.00	0.00	0.00	0.00	0.00	0.00	0.00	0.00	0.00	0.00	0.00	0.00	0.00	0.00	0.00	0.00	0.00	0.00
**E. coli phages**	1.00	0.00	0.00	0.00	0.00	0.00	0.00	0.00	0.00	0.00	0.00	0.00	0.00	0.00	0.00	0.00	0.00	0.00	0.00	0.00	0.00	0.00

ID		ET106	ET107	ET108	ET114	ET115	ET117	ET118	ET120	ET121	ET123	ET125	ET126	ET127	ET129	ET130	ET131	ET133	ET134	ET135	ET136	ET137
Fimbriae		F4	F18	F4	F18	F4	F18	F6	F4	F18	F4	F18	F5, F41	F18	F4	F18	F4	F18	F18	F5, F41	F18	F4
Toxins		ST1, ST2	ST2	ST1, ST2	ND	ST1, ST2	ND	ST2	ST1, ST2	ST1, ST2	ST1, ST2	ST1, ST2	ST1	ST1, ST2, LT	ST1, ST2, LT	ST1	ST1, ST2, LT	ST1	ST1, ST2	ST1	ST1, ST2	ST2
Serotype		O23:H37	O141ab/ac:H 4	H23:O8	O13/O135 :H48	O23:H37	O139:H1	O35:H6	O7:H15	O141ab/O141a c: H4/H15	O89/O162:H1 0	O131:H4	O9/O89/O16 2:H9	O138:H14	O149:H10	O131:H4	O149:H10	O98:H4	)141ab/ac:H	O9:H18	O138:H14	?:H20
**ETEP21B**	1.00	0.00	1.16	0.00	0.00	0.00	0.72	0.00	0.00	0.00	0.00	0.00	0.00	0.00	0.95	0.00	0.00	0.00	0.00	0.00	0.00	0.00
**ETEP102**	1.00	0.00	0.00	0.00	0.00	0.00	0.00	0.00	0.00	0.00	0.00	0.00	0.00	0.00	0.80	0.00	0.80	0.00	0.00	0.00	0.00	0.00
**CBA120**	1.00	0.00	0.00	0.00	0.00	0.00	0.00	0.00	0.00	0.00	0.00	0.00	0.00	0.00	0.00	0.00	0.00	0.00	0.00	0.00	0.00	0.00
**T3**	1.00	0.00	0.00	0.00	0.00	0.00	0.00	0.00	0.00	0.00	0.00	0.00	0.00	0.00	0.00	0.00	0.00	0.00	0.00	0.00	0.00	0.00
**T7**	1.00	0.00	0.00	0.00	0.00	0.00	0.00	0.00	0.00	0.00	0.00	0.00	0.00	0.00	0.00	0.00	0.00	0.00	0.00	0.00	0.00	0.00
**Salmonella phages**	1.00	0.00	0.00	0.00	0.00	0.00	0.00	0.00	0.00	0.00	0.00	0.00	0.00	0.00	0.00	0.00	0.00	0.00	0.00	0.00	0.00	0.00
**E. coli phages**	1.00	0.00	0.00	0.00	0.00	0.00	0.00	0.00	0.00	0.00	0.00	0.00	0.00	0.00	0.00	0.00	0.00	0.00	0.00	0.00	0.00	0.00

ID		ET138	ET139	ET140	ET141	ET142	ET143	ET145	ET146	ET148	ET150	ET152	ET154	ET155	ET158	ET159	ET160	ET161	ECOR04	MG1655
Fimbriae		F18	F4	F4	F18	F18	F18	F18	F4	F18	F4	F4	F4	F4	F18	F4	F4	F5	none	none
Toxins		ST1, ST2	ST2, LT	ST1, ST2, LT	ST1, ST2	ST1, ST2, LT	ST1, ST2	ST1, ST2, LT	ST1, ST2, LT	LT	ST1, ST2, LT	ST1, ST2, LT	ST1, ST2	ST2	ST1, ST2	ST2, LT	ST1, ST2	ST1, ST2	none	none
Serotype		O51:H42	O157:H39	O149:H10	O98:H4	O141ab/ac:H4	O98:H4	O76/O8:H19	O149:H10	O182:H4	?:H6	O149:H10	O23:H37	O98:H5	O166:H15	O138:H10	O23:H37	O149:H9	none	none
**ETEP21B**	1.00	0.00	0.00	0.00	0.00	0.00	0.00	0.00	0.00	0.00	0.00	1.00	0.00	0.00	0.00	1.00	0.00	0.98	1.00	1.00
**ETEP102**	1.00	0.00	0.00	0.00	0.00	0.00	0.00	0.00	0.80	0.00	0.00	0.00	0.00	0.00	0.00	0.00	0.00	0.00	1.00	1.00
**CBA120**	1.00	0.00	0.00	0.00	0.00	0.00	0.00	0.00	0.00	0.00	0.00	0.00	0.00	0.00	0.00	0.00	0.00	0.00	0.00	0.00
**T3**	1.00	0.00	0.00	0.00	0.00	0.00	0.00	0.00	0.00	0.00	0.00	0.00	0.00	0.00	0.00	0.00	0.00	0.00	1.00	1.00
**T7**	1.00	0.00	0.00	0.00	0.00	0.00	0.00	0.00	0.00	0.00	0.00	0.00	0.00	0.00	0.00	0.00	0.00	0.00	1.00	1.00
**Salmonella phages**	1.00	0.00	0.00	0.00	0.00	0.00	0.00	0.00	0.00	0.00	0.00	0.00	0.00	0.00	0.00	0.00	0.00	0.00	*	*
**E. coli phages**	1.00	0.00	0.00	0.00	0.00	0.00	0.00	0.00	0.00	0.00	0.00	0.00	0.00	0.00	0.00	0.00	0.00	0.00	*	*

SUPPLEMETAL FILE S4. Annotation table of one of the temperate phages isolated.

ORF	Product	Functional category	Region	Similarities and homologies in databases (InterPro, GeneScan, Pfam, HMMER, BLASTP, HHPRED)
ORF01	putative integrase		complement(104..1096)	HMMER: integrase in Klebsiella phage 3 LV-2017 (A0A2Z2FB15_9CAUD,e-value 5.1e-30) , Escherichia phage pro483 (A0A0F7LBR0_9CAUD, e-
ORF02	putative immunity repressor C		complement(1166..1507)	Pfam: helix-turn-helix (aa 12-58); HMMER: HTH cro/C1-type domain-containing protein in Salmonella phage FSLSP004 (S4TUC7_9CAUD, e-value 3.5e-59), gene C in Yersinia virus L413C (Q858U2_9CAUD, e-value 3.5e-59) and in Escherichia virus P2_4B2 (A0A653FS10_9CAUD, e-value 3.5e-59)
ORF03	putative regulatory protein cox		1611..2132	Pfam: Regulatory phage protein Cox (aa 52-130); HMMER: Ribosomal protein S15P/S13E in Escherichia phage pro147 (A0A0F7LDH4_9CAUD, e-value 3.5e-08) and Escherichia phage pro483 (A0A0F7LDH8_9CAUD, e-value 7.2e-08), and regulatory protein in Enterobacteria phage P88 (A0A0A7NPS5_9CAUD; e-value 1.1e-05)
ORF04	conserved hypothetical protein	Hypothetical	2143..2349	Phobius: transmembrane (aa 6-24 and 45-67); HMMER: uncharacterized protein in Citrobacter rodentium (D2TSL0_CITRI, e-value 2.5e-32) and D2TNR3_CITRI, e-value 1.3e-15)
ORF05	conserved hypothetical protein	Hypothetical	2361..2819	Pfam: DUF4761 domain (aa 48-152); HMMER: hypothetical prophage protein in Citrobacter rodentium (D2TNR2_CITRI, e-value 8.7e-68, and D2TSK9_CITRI, e-bvalue 1.0e-67), and uncharacterized protein in Enterobacteria phage P88 (A0A0A7NV42_9CAUD, 5.5e-67) and Escherichia coli O157:H7 (Q8XBH7_ECO57, e-value 2.1e-61)
ORF06	conserved hypothetical protein	Hypothetical	2830..3108	HMMER: uncharacterized protein in Escherichia coli O157:H7 (Q8XBH5_ECO57, e-value 8.9e-50) and in Enterobacteria phage P88 (A0A0A7NRX5_9CAUD, 7.7e-42), and prophage protein in Citrobacter rodentium (D2TNR1_CITRI, e-value 6.0e-46, and D2TSK8_CITRI, e-value 7.9e-45)
ORF07	conserved hypothetical protein	Hypothetical	3120..3362	HMMER: hypothetical prophage protein Citrobacter rodentium (D2TNR0_CITRI; e-value 1.2e-46); DNA modification methyltransferase in Enterobacteria phage P88 (A0A0A7NQ71_9CAUD, e-value 1.2e-46), and DUF4754 domain-containing protein in E.coli O157:H7 (Q8XBH3_ECO57; e-value 1.2e-45); Pfam: DUF4754
ORF08	conserved hypothetical protein	Hypothetical	3359..3472	HMMER: membrane protein in Enterobacteria phage P88 (A0A0A7NPW7_9CAUD, e-value 2.9e-17).
ORF09	conserved hypothetical protein	Hypothetical	3565..3981	HMMER: uncharacterized protein in E.coli O157:H7 (Q8XBH1_ECO57, e-value 2.2e-66)
ORF10	putative transcriptional regulator	DNA replication/trancription	4206..4451	Resistance Regulator (MarR) regulate the transcription of operon that encodes a drug efflux pump (Grove 2013, Current Biology)
ORF11	conserved hypothetical protein	Hypothetical	4448..4747	value 4.8e-51).
ORF12	putative derepression protein		5070..5300	value 2.6e-39)
ORF13	conserved hypothetical protein	Hypothetical	5373..5738	value 1.2e-71)
ORF14	endonuclease	DNA replication	5745..8510	Pfam: Bacteriophage replication gene A protein (GPA), likely to be endonucleases.
ORF15	putative old protein	moron	8691..9746	domain (AAA aa 133 - 259), often performing chaperone-like functions.
ORF16	putative tail fiber protein GpH	Tail morphogenesis	complement(9750..10805)	and in Escherichia phage pro483 (A0A0F7LBW5_9CAUD, e-value 3.9e-164), uncharacterized protein in Klebsiella phage 4LV2017
ORF17	putative tail formation protein GpI	Tail morphogenesis	complement(10802..11287)	Pfam: phage tail protein (Tail_P2_I, aa 1-101)
ORF18	putative baseplate assembly protein GpJ	Tail morphogenesis	complement(11403..12299)	3.0e-180) and in Enterobacteria phage P88 (A0A0A7NPY5_9CAUD, e-value 2.9e-179)
ORF19	putative baseplate assembly protein GpW	Tail morphogenesis	complement(12303..12653)	value 2.2e-70) and in Escherichia virus P88_4B11 (A0A653FT21_9CAUD, e-value 1.7e-69); GPW_gp25 domain-containing protein in Enterobacter
ORF20	putative baseplate assembly protein GpV	Tail morphogenesis	complement(12650..13231)	HMMER: baseplate assembly protein V in Enterobacteria phage P88 (A0A0A7NRZ3_9CAUD, e-value 1.1e-115) and in Escherichia virus P88_4B11
ORF21	putative tail completion protein GpS	Tail morphogenesis	complement(13228..13392)	Pfam: phage virion morphogenesis family (aa 1-50)
ORF22	putative transposase for insertion element		complement(14331..14606)	RAST: transposase InsO for insertion sequence element IS911; Pfam: helix-turn-helix like domain (aa 46-104), integrase core domain (aa 127-222 and 231-277); Phobius: two transmembrane domains (aa 82-100 and 145-163); HMMER: transposase or mobile element protein in Stx2-converting phage Stx2a_F422(A0A0P0ZAN1_9CAUD, e-value 7.8e-200), Stx converting phage vB_EcoS_ST2-8624 (A0A1I9LJW8_9CAUD, e-value 7.8e-200), Enterobacteria phage 2851 (B6ETC3_9CAUD, e-value 7.8e-200), Escherichia phage PA29(A0A0N7C209_9CAUD, e-value 7.8e-200), Escherichia phage Stx2 II (Q7Y2I6_9CAUD, e-value 7.8e-200), Escherichia phage PA28 (A0A0N7BTQ7_9CAUD, e-value 7.8e-200), Enterobacteria phage VT1-Sakai (Q7AK45_BPVT1, e-value 7.8e-200), Stx2-converting phage Stx2a_F765 (A0A0P0ZCU0_9CAUD, e-value 7.8e-200), Stx2-converting phage Stx2a_WGPS2 (A0A0P0ZEC8_9CAUD, e-value 7.8e-200), Stx2-converting phage Stx2a_F723 (A0A0N7KZD4_9CAUD, e-value 7.8e-200), Stx2-converting phage Stx2a_WGPS9 (A0A0P0ZCP4_9CAUD, e-value 4.3e-199)
ORF23	putative transposase for insertion elelment		complement(13444..14334)	RAST: Insertion element IS401 (Burkholderia multivorans) transposase; Pfam: transposase (aa 7-71); coil-coiled domain (aa 49-76); HMMER: Escherichia phage PA28 (A0A0N7BTN2_9CAUD, e-value 1.3e-56), Stx2-converting phage Stx2a_F765 (A0A0N7KZE6_9CAUD, e-value 2.4e-56), Stx2-converting phage Stx2a_WGPS9 (A0A0P0ZD52_9CAUD, e-value 2.4e-56), Enterobacteria phage BP-4795 (Q6H9S7_9CAUD, e-value 2.4e-56), Stx2-converting phage Stx2a_F723 (A0A0P0ZBX5_9CAUD, e-value 2.4e-56), Stx2-converting phage Stx2a_WGPS2 (A0A0N7KZG5_9CAUD, e-value 2.4e-56), Stx2-converting phage 1717 (B6DZX4_9CAUD, e-value 2.4e-56), Stx converting phage vB_EcoS_ST2-8624 (A0A1I9LJW9_9CAUD, e-value 2.4e-56), Stx2-converting phage Stx2a_F422 (A0A0P0ZC46_9CAUD, e-value 2.4e-56)
ORF24	putative tail protein	Tail morphogenesis	complement(14709..15176)	Pfam: phage virion morphogenesis family (aa 3-87); HMMER: tail protein in Enterobacteria phage P88 (A0A0A7NV60_9CAUD, e-value 8.3e-96) and phage neck protein in Escherichia virus P88_4B11 (A0A653FSF7_9CAUD, e-value 1.9e-95)
ORF25	putative tail completion protein GpR	Tail morphogenesis	complement(15169..15636)	Pfam: P2 phage tail completion protein GpR (aa 1-129); HMMER: tail completion protein in Enterobacteria phage P88 (A0A0A7NV60_9CAUD, e-value 1.2e-101), Escherichia virus P88_4B11 (A0A653FSF7_9CAUD, e-value 1.2e-101)
ORF26	conserved hypothetical protein	Hypothetical	complement(15774..16181)	Phobius: signal peptide (aa 1-22); coiled coil (aa 55-75); HMMER: uncharacterized protein in Escherichia virus P88_4B11 (A0A653FT10_9CAUD, e-value 2.3e-83) and in Enterobacteria phage P88 (A0A0A7NPY2_9CAUD, e-value 4.4e-83)
ORF27	putative endolysin	Lysis	complement(16178..16570)	RAST: peptidoglycan hydrolase; Pfam: D-alanyl-D-alanine carboxypeptidase (aa 56-123); HMMER: phage related protein in Escherichia virus P88_4B11 (A0A653FSH6_9CAUD, e-value 2.5e-84); endolysin in Enterobacteria phage P88 (A0A0A7NQ86_9CAUD, e-value 2.5e-84), Salmonella phage 9NA (A0A060DAL8_9CAUD, e-value 1.0e-62), Salmonella phage SP069 (S4TRL9_9CAUD, e-value 7.3e-62), A0A1P8DTR0_9CAUD (Salmonella phage vB_SenS_Sasha, e-value 1.7e-61), Salmonella phage Solent (A0A385IRS4_9CAUD, e-value 1.7e-61) and Enterobacteria phage phiP27 (Q9MBZ3_9CAUD, e-value 1.8e-61)
ORF28	putative holin	Lysis	complement(16567..16890)	RAST: COG1263: Phosphotransferase system II components, glucose/maltose/N-acetylglucosamine-specific; Pfam: phage holin family - lysis protein S (aa 4-101); HMMER: Escherichia virus P88_4B11 (A0A653FSG1_9CAUD, e-value 1.2e-63), Enterobacteria phage P88 (A0A0A7NRY9_9CAUD, e-value 4.7e-63)
ORF29	putative tail component protein GpX	Tail morphogenesis	complement(16893..17093)	Pfam: Phage Tail Protein X (aa 1-58), related to LysM domain; InterPro:included to the phage tail protein GpX protein from Bacteriophage P2, with suggested a general peptidoglycan binding function. HMMER: Tail component protein in Enterobacteria phage P88 (A0A0A7NV57_9CAUD, e-value 1.3e-39) and Escherichia virus P88_4B11 (A0A653FTL4_9CAUD, e-value 9.2e-39)
ORF30	putative head completion protein GpL	Capsid morphogenesis	complement(17093..17587)	Pfam: Phage head completion protein GPL, it allows the completion of filled heads by rendering newly packaged DNA in the heads resistant to DNase (aa 26-164); HMMER: head completion-stabilization protein in Enterobacteria phage P88 (A0A0A7NPU2_9CAUD, e-value 1.2e-104) and Ad1 Phage adapter protein in Escherichia virus P88_4B11 (A0A653FUI5_9CAUD, e-value 4.5e-102).
ORF31	putative terminase, small subunit GpM	DNA packaging	complement(17690..18490)	RAST: phage terminase, endonuclease subunit GpM; Pfam: small terminase subunit (aa 36-169); HMMER: terminase endonuclease subunit in Enterobacteria phage P88 (A0A0A7NPX9_9CAUD, e-value 2.5e-166) and Escherichia virus P88_4B11 (A0A653FSN9_9CAUD, e-value 5.8e-165)
ORF32	major capsid protein GpN	Capsid morphogenesis	complement(18536..19588)	7.7e-233)
ORF33	putative capsid scaffolding protein GpO	Capsid morphogenesis	complement(19612..20448)	Pfam: Phage capsid scaffolding protein (GPO) serine peptidase (aa 9-278); HMMER: capsid scaffolding protein in Escherichia virus P88_4B11 (A0A653FSE9_9CAUD, e-value 4.6e-177) and Enterobacteria phage P88(A0A0A7NRY7_9CAUD, e-value 1.7e-176)
ORF34	putative terminase, large subunit GpP	DNA packaging	20603..22354	RAST: terminase, ATPase subunit GpP; Pfam: Putative ATPase subunit of terminase (aa 6-63), N-terminal terminase large subunit (aa 150-376) and terminase RNaseH-like domain (aa 405-567); HMMER: Phage terminase, ATPase subunit in Escherichia virus P88_4B11 (A0A653FUM3_9CAUD, e-value 0.0e+00) and Enterobacteria phage P88 (A0A0A7NV54_9CAUD, e-value 0.0e+00)
ORF35	putative portal vertex protein GpQ	Capsid morphogenesis	22354..23400	Pfam: phage protal (aa 89-348); HMMER: PBSX family portal protein in Enterobacteria phage P88 (A0A0A7NPT9_9CAUD, e-value 1.3e-239) and Presumed portal vertex protein in Escherichia virus P88_4B11 (A0A653FSZ6_9CAUD, e-value 6.4e-239)
ORF36	conserved hypothetical protein	Hypothetical	complement(23469..23597)	HMMER: uncharacterized protein in Enterobacteria phage P88 (A0A0A7NPX6_9CAUD, e-value 3.8e-15) and Escherichia virus P88_4B11 (A0A653FSG7_9CAUD, e-value 8.4e-14)
ORF37	putative DNA invertase	DNA replication	23790..24389	Pfam: Resolvase, N terminal domain (aa 2-136) and Helix-turn-helix domain of resolvase (aa 138-181); HMMER: DNA invertase in Escherichia virus P88_4B11 (A0A653FUP2_9CAUD; e-value 2.6e-103) and Enterobacteria phage P88 (A0A0A7NPV4_9CAUD, e-value 6.2e-102)
ORF38	putative tail assembly protein GpU	Tail morphogenesis	complement(24418..24906)	98) and Enterobacteria phage P88 (A0A0A7NV65_9CAUD, e-value 2.2e-97)
ORF39	putative tape-measure protein GpT	Tail morphogenesis	complement(24918..27719)	domains (525-551; 558-580; 586-613); three coiled coil domains (aa 27-61; 86-113; 135-169); HMMER: tape measure protein in Escherichia virus
ORF40	putative P2 GpE family protein		complement(27706..27861)	Pfam: Phage P2 tail protein GpE (aa 10-46); HMMER: P2 tail protein GpE in Enterobacteria phage P88 (A0A0A7NQ96_9CAUD, e-value 1.9e-29) and Escherichia virus P88_4B11 (A0A653FT30_9CAUD, e-value 5.4e-28)
ORF41	putative tail protein	Tail morphogenesis	complement(27870..28238)	(A0A0A7NPZ0_9CAUD, e-value 5.7e-63) and Escherichia virus P88_4B11 (A0A653FSQ7_9CAUD, e-value 1.3e-45)
ORF42	putative major tail tube protein GpFII	Tail morphogenesis	complement(28293..28805)	Pfam: Phage tail tube protein FII (aa 4 - 168); HMMER: Phage major tail tube protein in Escherichia virus P88_4B11 (A0A653FUK6_9CAUD, e-value 6.6e-108) and Enterobacteria phage P88 (A0A0A7NPV8_9CAUD, e-value 3.5e-104)
ORF43	putative tail sheath monomer GpFI	Tail morphogenesis	complement(28805..29989)	Pfam: tail sheath protein subtilisin-like domain (aa 110 - 278) and tail sheath C-terminal domain (aa 279 - 380); HMMER: tail sheath monomer in Escherichia virus P88_4B11 (A0A653FTM9_9CAUD, e-value 1.7e-254) and Enterobacteria phage P88 (A0A0A7NV69_9CAUD, e-value 1.2e-253)
ORF44	putative tail baseplate hub protein GpD	Tail morphogenesis	30147..31247	Pfam: Phage tail baseplate hub (aa 45 - 347); HMMER: Gene D protein in Enterobacteria phage P88 (A0A0A7NQ97_9CAUD, e-value 1.8e-242) and Escherichia virus P88_4B11 (A0A653FSH8_9CAUD, e-value 5.7e-240)
ORF45	putative transcriptional activator		31290..31550	RAST: Ogr_Delta domain-containing protein; Pfam: Ogr/Delta-like zinc finger, usually transcriptional activators (PF04606, aa 3-51); HMMER: Ogr_Delta domain-containing protein in Enterobacter phage phiT5282H (A0A386K5D2_9CAUD, e-value 3.7e-14), Klebsiella phage ST16-OXA48phi5.4 (A0A482ILV6_9CAUD, e-value 2.5e-13)
ORF46	conserved hypothetical protein	Hypothetical	31672..32028	HMMER: uncharacterized protein in Cronobacter phage ESSI-2 (F1BUJ7_9CAUD, e-value 6.3e-15), Haemophilus phage HP1 (YO35_BPHC1, e-value 1.7e-07), Salinivibrio phage SMHB1e-value (A0A1D9C9S3_9CAUD, 4.3e-07)
ORF47	conserved hypothetical protein	Hypothetical	32087..32575	(Q7N2M0_PHOLL, e-value 1.1e-34), Pantoea coffeiphila (A0A2S9I4D8_9GAMM, e-value 6.7e-09)
ORF48	conserved hypothetical protein	Hypothetical	32575..32955	Pfam: YafO_toxin family, type II toxin-antitoxin system (aa 25-125); HMMER: uncharacterized protein in Enterobacteria phage CUS-3 (A5VW62_9CAUD, e-value 8.4e-12)
ORF49	conserved hypothetical protein	Hypothetical	32957..33136	HMMER: uncharacterized protein in Ficedula albicollis (U3JPK1_FICAL, e-valu 3.2e-07)

SUPPLEMENTAL FILE S5. Clinker visualization of genome alignment of phages in the clusters of A) ETEP21B, corresponding to the *Berlinvirus* phages, and B) ETEP102, including some *Drexlerviridae* phages. C) Phages deposited in databases similar to ETEP21B and ETEP102, according to vCONTACT.

A)

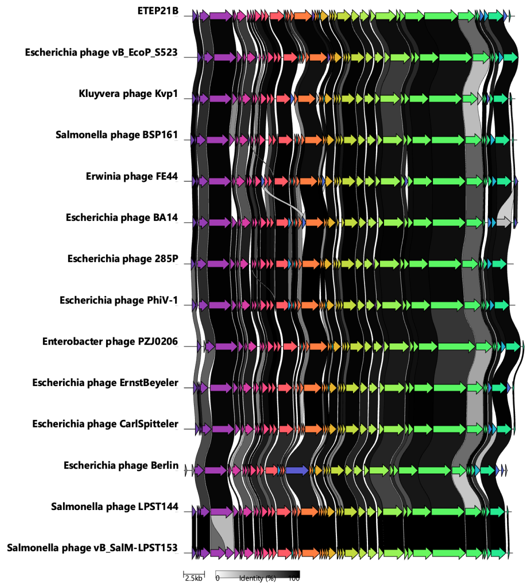

B)

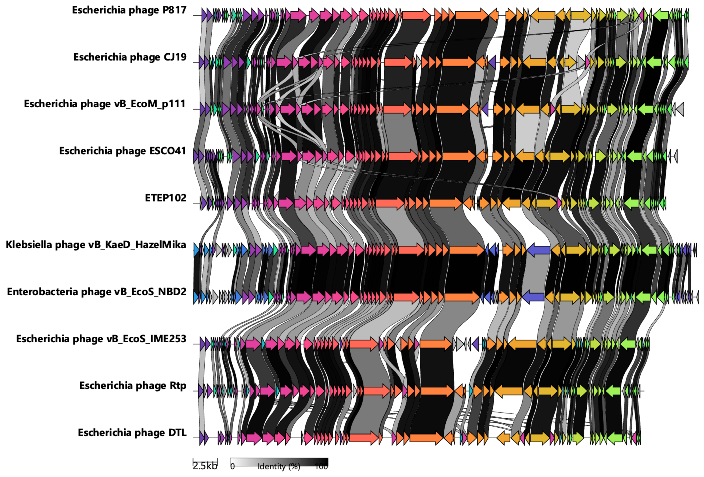

C)

**ETEP21B cluster on vCONTACT**																
357	Citrobacter~phage~SH1	Caudovirales	Autographiviridae	Teetrevirus	277_0	Clustered	59	VC_277_0	59	0.754	1	0.754	11	3	2	1
358	Citrobacter~phage~SH2	Caudovirales	Autographiviridae	Teetrevirus	277_0	Clustered	59	VC_277_0	59	0.754	1	0.754	11	3	2	1
362	Citrobacter~phage~phiCFP-1	Caudovirales	Autographiviridae	Teetrevirus	277_0	Clustered	59	VC_277_0	59	0.754	1	0.754	11	3	2	1
476	Enterobacter~phage~E-2	Caudovirales	Autographiviridae	Teetrevirus	277_0	Clustered	59	VC_277_0	59	0.754	1	0.754	11	3	2	1
477	Enterobacter~phage~E-3	Caudovirales	Autographiviridae	Teetrevirus	277_0	Clustered	59	VC_277_0	59	0.754	1	0.754	11	3	2	1
490	Enterobacteria~phage~13a	Caudovirales	Autographiviridae	Teseptimavirus	277_0	Clustered	59	VC_277_0	59	0.754	1	0.754	11	3	2	1
491	Enterobacteria~phage~285P	Caudovirales	Autographiviridae	Teseptimavirus	277_0	Clustered	59	VC_277_0	59	0.754	1	0.754	11	3	2	1
493	Enterobacteria~phage~BA14	Caudovirales	Autographiviridae	Berlinvirus	277_0	Clustered	59	VC_277_0	59	0.754	1	0.754	11	3	2	1
521	Enterobacteria~phage~T3	Caudovirales	Autographiviridae	Teetrevirus	277_0	Clustered	59	VC_277_0	59	0.754	1	0.754	11	3	2	1
522	Enterobacteria~phage~T7M	Caudovirales	Autographiviridae	Teetrevirus	277_0	Clustered	59	VC_277_0	59	0.754	1	0.754	11	3	2	1
535	Enterobacteria~phage~vB_EcoP_IME390	Caudovirales	Autographiviridae	Teseptimavirus	277_0	Clustered	59	VC_277_0	59	0.754	1	0.754	11	3	2	1
571	Erwinia~phage~FE44	Caudovirales	Autographiviridae	Berlinvirus	277_0	Clustered	59	VC_277_0	59	0.754	1	0.754	11	3	2	1
603	Erwinia~phage~vB_EamP-L1	Caudovirales	Autographiviridae	Elunavirus	277_0	Clustered	59	VC_277_0	59	0.754	1	0.754	11	3	2	1
613	Escherichia~phage~64795_ec1	Caudovirales	Autographiviridae	Teseptimavirus	277_0	Clustered	59	VC_277_0	59	0.754	1	0.754	11	3	2	1
621	Escherichia~phage~C5	Caudovirales	Autographiviridae	Teseptimavirus	277_0	Clustered	59	VC_277_0	59	0.754	1	0.754	11	3	2	1
623	Escherichia~phage~CICC~80001	Caudovirales	Autographiviridae	Teseptimavirus	277_0	Clustered	59	VC_277_0	59	0.754	1	0.754	11	3	2	1
628	Escherichia~phage~ECA2	Caudovirales	Autographiviridae	Teetrevirus	277_0	Clustered	59	VC_277_0	59	0.754	1	0.754	11	3	2	1
634	Escherichia~phage~EG1	Caudovirales	Autographiviridae	Teseptimavirus	277_0	Clustered	59	VC_277_0	59	0.754	1	0.754	11	3	2	1
639	Escherichia~phage~Ebrios	Caudovirales	Autographiviridae	Teseptimavirus	277_0	Clustered	59	VC_277_0	59	0.754	1	0.754	11	3	2	1
653	Escherichia~phage~HZ2R8	Caudovirales	Autographiviridae	Teseptimavirus	277_0	Clustered	59	VC_277_0	59	0.754	1	0.754	11	3	2	1
654	Escherichia~phage~HZP2	Caudovirales	Autographiviridae	Teseptimavirus	277_0	Clustered	59	VC_277_0	59	0.754	1	0.754	11	3	2	1
673	Escherichia~phage~LL2	Caudovirales	Autographiviridae	Teetrevirus	277_0	Clustered	59	VC_277_0	59	0.754	1	0.754	11	3	2	1
682	Escherichia~phage~N30	Caudovirales	Autographiviridae	Teseptimavirus	277_0	Clustered	59	VC_277_0	59	0.754	1	0.754	11	3	2	1
688	Escherichia~phage~P483	Caudovirales	Autographiviridae	Berlinvirus	277_0	Clustered	59	VC_277_0	59	0.754	1	0.754	11	3	2	1
689	Escherichia~phage~P694	Caudovirales	Autographiviridae	Berlinvirus	277_0	Clustered	59	VC_277_0	59	0.754	1	0.754	11	3	2	1
703	Escherichia~phage~SRT7	Caudovirales	Autographiviridae	Foetvirus	277_0	Clustered	59	VC_277_0	59	0.754	1	0.754	11	3	2	1
715	Escherichia~phage~T7	Caudovirales	Podoviridae	T7virus	277_0	Clustered	59	VC_277_0	59	0.754	1	0.754	11	3	2	1
776	Escherichia~phage~vB_EcoP_S523	Caudovirales	Autographiviridae	Berlinvirus	277_0	Clustered	59	VC_277_0	59	0.754	1	0.754	11	3	2	1
1086	Kluyvera~phage~Kvp1	Caudovirales	Podoviridae	T7virus	277_0	Clustered	59	VC_277_0	59	0.754	1	0.754	11	3	2	1
1257	Leclercia~phage~10164-302	Caudovirales	Autographiviridae	Teetrevirus	277_0	Clustered	59	VC_277_0	59	0.754	1	0.754	11	3	2	1
1347	Morganella~phage~MmP1	Caudovirales	Autographiviridae	Minipunavirus	277_0	Clustered	59	VC_277_0	59	0.754	1	0.754	11	3	2	1
1348	Morganella~phage~vB_MmoP_MP2	Caudovirales	Autographiviridae	Minipunavirus	277_0	Clustered	59	VC_277_0	59	0.754	1	0.754	11	3	2	1
1726	**P21B_contig1**	Unassigned	Unassigned	Unassigned	277_0	Clustered	59	VC_277_0	59	0.754	1	0.754	11	3	2	1
1765	Pectobacterium~phage~Jarilo	Caudovirales	Autographiviridae	Jarilovirus	277_0	Clustered	59	VC_277_0	59	0.754	1	0.754	11	3	2	1
1776	Pectobacterium~phage~PP47	Caudovirales	Autographiviridae	Pektosvirus	277_0	Clustered	59	VC_277_0	59	0.754	1	0.754	11	3	2	1
1777	Pectobacterium~phage~PP74	Caudovirales	Autographiviridae	Berlinvirus	277_0	Clustered	59	VC_277_0	59	0.754	1	0.754	11	3	2	1
1778	Pectobacterium~phage~PP81	Caudovirales	Autographiviridae	Pektosvirus	277_0	Clustered	59	VC_277_0	59	0.754	1	0.754	11	3	2	1
1781	Pectobacterium~phage~PPWS4	Caudovirales	Autographiviridae	Pektosvirus	277_0	Clustered	59	VC_277_0	59	0.754	1	0.754	11	3	2	1
1793	Phage~NC-A	Caudovirales	Autographiviridae	Teseptimavirus	277_0	Clustered	59	VC_277_0	59	0.754	1	0.754	11	3	2	1
2191	Salmonella~phage~3A_8767	Caudovirales	Autographiviridae	Teseptimavirus	277_0	Clustered	59	VC_277_0	59	0.754	1	0.754	11	3	2	1
2195	Salmonella~phage~BP12A	Caudovirales	Autographiviridae	Berlinvirus	277_0	Clustered	59	VC_277_0	59	0.754	1	0.754	11	3	2	1
2204	Salmonella~phage~BSP161	Caudovirales	Autographiviridae	Berlinvirus	277_0	Clustered	59	VC_277_0	59	0.754	1	0.754	11	3	2	1
2290	Salmonella~phage~Vi06	Caudovirales	Autographiviridae	Teseptimavirus	277_0	Clustered	59	VC_277_0	59	0.754	1	0.754	11	3	2	1
2314	Salmonella~phage~phiSG-JL2	Caudovirales	Autographiviridae	Teetrevirus	277_0	Clustered	59	VC_277_0	59	0.754	1	0.754	11	3	2	1
2347	Serratia~phage~2050H2	Caudovirales	Autographiviridae	Teetrevirus	277_0	Clustered	59	VC_277_0	59	0.754	1	0.754	11	3	2	1
2352	Serratia~phage~SM9-3Y	Caudovirales	Autographiviridae	Teetrevirus	277_0	Clustered	59	VC_277_0	59	0.754	1	0.754	11	3	2	1
2386	Shigella~phage~VB_Ship_A7	Caudovirales	Autographiviridae	Berlinvirus	277_0	Clustered	59	VC_277_0	59	0.754	1	0.754	11	3	2	1
2546	Stenotrophomonas~phage~IME15	Caudovirales	Autographiviridae	Teseptimavirus	277_0	Clustered	59	VC_277_0	59	0.754	1	0.754	11	3	2	1
2816	Yersinia~phage~Berlin	Caudovirales	Autographiviridae	Berlinvirus	277_0	Clustered	59	VC_277_0	59	0.754	1	0.754	11	3	2	1
2819	Yersinia~phage~PYPS50	Caudovirales	Autographiviridae	Berlinvirus	277_0	Clustered	59	VC_277_0	59	0.754	1	0.754	11	3	2	1
2820	Yersinia~phage~Yep-phi	Caudovirales	Autographiviridae	Berlinvirus	277_0	Clustered	59	VC_277_0	59	0.754	1	0.754	11	3	2	1
2821	Yersinia~phage~Yepe2	Caudovirales	Autographiviridae	Berlinvirus	277_0	Clustered	59	VC_277_0	59	0.754	1	0.754	11	3	2	1
2822	Yersinia~phage~YpP-Y	Caudovirales	Autographiviridae	Teseptimavirus	277_0	Clustered	59	VC_277_0	59	0.754	1	0.754	11	3	2	1
2823	Yersinia~phage~YpsP-G	Caudovirales	Autographiviridae	Teseptimavirus	277_0	Clustered	59	VC_277_0	59	0.754	1	0.754	11	3	2	1
2827	Yersinia~phage~phiA1122	Caudovirales	Autographiviridae	Teseptimavirus	277_0	Clustered	59	VC_277_0	59	0.754	1	0.754	11	3	2	1
2832	Yersinia~phage~phiYe-F10	Caudovirales	Autographiviridae	Teetrevirus	277_0	Clustered	59	VC_277_0	59	0.754	1	0.754	11	3	2	1
2833	Yersinia~phage~phiYeO3-12	Caudovirales	Autographiviridae	Teetrevirus	277_0	Clustered	59	VC_277_0	59	0.754	1	0.754	11	3	2	1
2835	Yersinia~phage~vB_YenP_AP10	Caudovirales	Autographiviridae	Apdecimavirus	277_0	Clustered	59	VC_277_0	59	0.754	1	0.754	11	3	2	1
2836	Yersinia~phage~vB_YenP_AP5	Caudovirales	Autographiviridae	Teetrevirus	277_0	Clustered	59	VC_277_0	59	0.754	1	0.754	11	3	2	1
**ETEP102 cluster on vCONTACT**																
209	Citrobacter~virus~Stevie	Caudovirales	Siphoviridae	Tlsvirus	193_0	Clustered	26	VC_193_0	26	0.9751	1	0.9751	6	2	2	0.9692
259	Cronobacter~virus~ESP29491	Unassigned	Unassigned	Unassigned	193_0	Clustered	26	VC_193_0	26	0.9751	1	0.9751	6	2	2	0.9692
360	Enterobacteria~phage~vB_EcoS_NBD2	Unassigned	Unassigned	Unassigned	193_0	Clustered	26	VC_193_0	26	0.9751	1	0.9751	6	2	2	0.9692
361	Enterobacteria~phage~vB_EcoS_Rogue1	Caudovirales	Siphoviridae	Rogue1virus	193_0	Clustered	26	VC_193_0	26	0.9751	1	0.9751	6	2	2	0.9692
415	Escherichia~phage~ADB-2	Caudovirales	Siphoviridae	T1virus	193_0	Clustered	26	VC_193_0	26	0.9751	1	0.9751	6	2	2	0.9692
422	Escherichia~phage~EB49	Caudovirales	Siphoviridae	Rogue1virus	193_0	Clustered	26	VC_193_0	26	0.9751	1	0.9751	6	2	2	0.9692
468	Escherichia~phage~e4/1c	Caudovirales	Siphoviridae	Rogue1virus	193_0	Clustered	26	VC_193_0	26	0.9751	1	0.9751	6	2	2	0.9692
501	Escherichia~phage~vB_EcoS_AHP42	Caudovirales	Siphoviridae	Rogue1virus	193_0	Clustered	26	VC_193_0	26	0.9751	1	0.9751	6	2	2	0.9692
502	Escherichia~phage~vB_EcoS_AHS24	Caudovirales	Siphoviridae	Rogue1virus	193_0	Clustered	26	VC_193_0	26	0.9751	1	0.9751	6	2	2	0.9692
503	Escherichia~phage~vB_EcoS_AKS96	Caudovirales	Siphoviridae	Rogue1virus	193_0	Clustered	26	VC_193_0	26	0.9751	1	0.9751	6	2	2	0.9692
505	Escherichia~phage~vB_Eco_ACG-M12	Caudovirales	Siphoviridae	Rtpvirus	193_0	Clustered	26	VC_193_0	26	0.9751	1	0.9751	6	2	2	0.9692
520	Escherichia~virus~KP26	Caudovirales	Siphoviridae	Rogue1virus	193_0	Clustered	26	VC_193_0	26	0.9751	1	0.9751	6	2	2	0.9692
526	Escherichia~virus~Rtp	Caudovirales	Siphoviridae	Rtpvirus	193_0	Clustered	26	VC_193_0	26	0.9751	1	0.9751	6	2	2	0.9692
528	Escherichia~virus~T1	Caudovirales	Siphoviridae	T1virus	193_0	Clustered	26	VC_193_0	26	0.9751	1	0.9751	6	2	2	0.9692
530	Escherichia~virus~TLS	Caudovirales	Siphoviridae	Tlsvirus	193_0	Clustered	26	VC_193_0	26	0.9751	1	0.9751	6	2	2	0.9692
640	Klebsiella~phage~1513	Caudovirales	Siphoviridae	Kp36virus	193_0	Clustered	26	VC_193_0	26	0.9751	1	0.9751	6	2	2	0.9692
646	Klebsiella~phage~KLPN1	Caudovirales	Siphoviridae	Kp36virus	193_0	Clustered	26	VC_193_0	26	0.9751	1	0.9751	6	2	2	0.9692
651	Klebsiella~phage~KP36	Caudovirales	Siphoviridae	Kp36virus	193_0	Clustered	26	VC_193_0	26	0.9751	1	0.9751	6	2	2	0.9692
659	Klebsiella~phage~PKP126	Caudovirales	Siphoviridae	Kp36virus	193_0	Clustered	26	VC_193_0	26	0.9751	1	0.9751	6	2	2	0.9692
660	Klebsiella~phage~Sushi	Caudovirales	Siphoviridae	Kp36virus	193_0	Clustered	26	VC_193_0	26	0.9751	1	0.9751	6	2	2	0.9692
1100	**P102_contig2**	Unassigned	Unassigned	Unassigned	193_0	Clustered	26	VC_193_0	26	0.9751	1	0.9751	6	2	2	0.9692
1380	Salmonella~phage~36	Unassigned	Unassigned	Unassigned	193_0	Clustered	26	VC_193_0	26	0.9751	1	0.9751	6	2	2	0.9692
1436	Salmonella~phage~phSE-2	Unassigned	Unassigned	Unassigned	193_0	Clustered	26	VC_193_0	26	0.9751	1	0.9751	6	2	2	0.9692
1482	Shigella~phage~pSf-1	Unassigned	Unassigned	Unassigned	193_0	Clustered	26	VC_193_0	26	0.9751	1	0.9751	6	2	2	0.9692
1483	Shigella~phage~pSf-2	Caudovirales	Siphoviridae	T1virus	193_0	Clustered	26	VC_193_0	26	0.9751	1	0.9751	6	2	2	0.9692
1486	Shigella~virus~Shfl1	Caudovirales	Siphoviridae	T1virus	193_0	Clustered	26	VC_193_0	26	0.9751	1	0.9751	6	2	2	0.9692
