# Supplementary material for: Diversity and phage sensitivity to phages of porcine enterotoxigenic Escherichia coli
Source: Appl Environ Microbiol. 2024 Jun 28;90(7):e00807-24. doi: 10.1128/aem.00807-24 (PMC11267873; doi:10.1128/aem.00807-24)
Supplement: Supplemental material part II — Files S6 to S10. [file aem.00807-24-s0002.pdf]

SUPPLEMENTAL FILES 6-10

SUPPLEMENTAL FILE S6. A) ETEP21B full annotation table; The early transcribed genes were involved in evading the host defense systems (ORF01, ORF06, ORF17, ORF23) and shutoff the host transcription and switch to the viral transcription (ORF03, ORF04, ORF10). The middle genes encoded other transcriptional factors (ORF07, ORF09, ORF13, ORF22), and concur to DNA replication (ORF11, ORF12, ORF14, ORF19, ORF21, ORF26). The late genes code for head (ORF28, ORF29, ORF30 and ORF31, ORF34 to ORF37) and the tail apparatus (ORF2, ORF32, ORF3333, ORF38). The lysis cassette was composed by a type II holin (ORF39) and the i-spanin with o-spanin embedded (ORF41 and 42), unconventionally separated by the small terminase subunit (ORF40). The small and large subunits of terminase (ORF40 and 44) were responsible for the DNA packaging and the duplication of the two terminal repeats that in ETEP21B were identified as 145 bp-long. Two ORFs (20 and 45) were identified as HNH endonucleases that concur to phage genome variability. B) Dcm and Dam sites in *Berlinvirus* phages; C) Identification of the ETEP21B receptor by infection on phage receptor mutants: pictures of plates with 3-log reduced infection of ETEP21B on ET54 mutant in *waaC* compared to the wild type.

A)

ORF	Region	Product	Functional category	Region	Similarities and homologies in databases (InterPro, GeneScan, Pfam, HMMER, BLASTP, HHPRED)	Translation
1	early	ORF01; putative S-adenosyl-L-methionine hydrolase	resistance	1056..1553	T3 (e-value 9.8e-89); uniprot: P07693.	MIHTKEQANV
2	early	ORF02; hypothetical conserved protein	Hypothetical	1555..1752	HMMER: uncharacterized protein in Kluyvera phage Kvp1 (B6Z9E3_9CAUD, e-value 4.2e-37); Phobius: signal peptide aa 1-17. TMHMM: transmemenrae protein - N in C out in nt 1558..1749	MVALFVITVYA
3	early	ORF03; putative protein kinase	DNA	1921..3024	HMMER: protein kinase in Kluyvera phage Kvp1 (e-value 9.0e-225) and E.coli phage T7 (e-value 1.2e-111).	MNYTDIQARL
4	early	ORF04; putative DNA-directed RNA polymerase	DNA	3098..5749	BLASTP and HMMER: RNA polymerase Enterobacteria phage T7 (e-value 0.0).	MTNVINAPKN
5	early	ORF05; putative hypothetical conserved protein	Hypothetical	5842..5991	BLASTP and HMMMER: uncharaterized protein in Kluyvera phage Kvp1 (B6Z9E8_9CAUD, e-value 9.4e-27).	MRTNFEPTTK
6	early	ORF06; putative host dGTPase inhibitor	Evasion host	6365..7384	BLASTP: deoxyguanosine triphospho-hydrolase inhibitor in Kluyvera phage Kvp1 (98% query coverage; 92.22% identity). HMMER: Deoxyguanosine triphospho-hydrolase inhibitor in Enterobacteria phage T3 (Q778M5_BPT3, e-value 2.2e-	MGRLYSGNLIA
7	early	ORF07; putative ATP-dependent DNA ligase	DNA	7553..7831	Pfam: ATP dependent DNA ligase (aa 9-239) and DNA ligase C-terminal domain (aa 240-270); HMMER: DNA ligase in Enterobacteria phage T7 (DNLI_BPT7, e-value 2.0e-149); BLASTP: DNA ligase in Enterobacteria phage 285P (99%	MEITFKTNPFK
8	middle	ORF08; hypothetical conserved protein	Hypothetical	7936..8142	Pfam: Bladder cancer-related protein BC10 domain; HMMER: uncharacterized protein phage (B6Z9F2_9CAUD, e-value 6.5e-49).	MEETLMLRLH
9	middle	ORF09; putative nucleotide kinase	DNA	8230..8421	BLASTP: hypothetical protein in Escherichia phage vB_EcoP_S523 (98% query coverage, 97% identity), nucleotide kinase in Yersinia phage PYPS50 (98% query coverage, 97% identity) and nucleotide kinase gp 1.7 in Enterobacteria phage	MLFDDIEAIEVI
10	middle	ORF10; putative bacterial RNA polymerase inhibitor	Evasion host	8489..9196	Pfam: RNA polymerase inhibitor (aa 14-60); BLASTP: host RNA polymerase inhibitor Salmonella phage vB_SalM-LPST153 (98% query coverage, 100% identity).	MSNLNKGSLV
11	middle	ORF11; putative ssDNA-binding protein	DNA	9196..9642	Pfam: disorder domain aa 128 and 194-236; BLASTP: single-stranded DNA-binding protein in Salmonella phage vB_SalM-LPST153 (99% coverage, 98% identity).	MAFNKRKVFTS
12	middle	ORF12; putative endonuclease I	DNA	9639..10094	BLASTP: endonuclease I in Yersinia phage PYPS50 (99% coverage, 100% identity); Pfam: PF05367.11, Phage endonuclease I aa 1-147.	MAKGYAGRGT
13	middle	ORF13; putative lysozyme	DNA	9639..10094	Pfam: N-acetylmuramoyl-L-alanine amidase domain aa 12-132; BLASTP: N-acetylmuramoyl-L-alanine amidase in Kluyvera phage Kvp1 (99% coverage, 98% identity) and endolysin in Yersinia phage PYPS50 (99% coverage, 95% identity).	MSKVQFKPRA
14	middle	ORF14; putative DNA primase	DNA	10262..11974	Pfam: DNA primase/helicase in Salmonella phage BP12A (aa 291-542) and topoisomerase-primase (aa 157-244) domains; BLASTP: DNA primase/helicase in Salmonella phage BP12A (99% coverage, 99% identity).	MDMEEQQESI
15	middle	ORF15; hypothetical conserved protein	Hypothetical	11928..12113	BLASTP: hypothetical protein BP12A_16 in Salmonella phage BP12A (coverage 99.8%, identity 98%); IUPred: disorder domains.	MEIAAGNQKK
16	middle	ORF16; hypothetical conserved protein	Hypothetical	12106..12318	Pfam: DUF5471, found in Enterobacteria phage T7 (aa 1-69) and coiled-coil domain (aa 17-44); BLASTP: hypothetical protein vBSalMLPST153_orf00028 in Salmonella phage vB_SalM-LPST153 (98% coverage, identity 98.57%).	MFKFINTLGKL
17	middle	ORF17; putative inhibitor of toxin/antitoxin system	Evasion host	12332..12637	Pfam: Inhibitor of toxin/antitoxin system (Gp4.5) (aa 8-75); Uniprot: B3VCP5_9CAUD - Gp4.5 in Enterobacteria phage BA14 (e-value 4.2e-56); BLASTP: hypothetical protein P694_30 in Escherichia phage P694 (98% coverage, 90.00%	MAIKFPGNTIR
18	middle	ORF18; hypothetical conserved protein	Hypothetical	12725..13141	BLASTP: hypothetical protein vBSalMLPST153_orf00026 in Salmonella phage vB_SalM-LPST153 (coverage 99%, identity 94.93%); Coiled-coil domain aa 4-24.	MRNRNVIESEI
19	middle	ORF19; putative DNA polymerase	DNA	13154..15268	Pfam: RNase_H superfamily (aa 5-185) and DNA polymerase (aa 330-701) domains; BLASTP: DNA polymerase in Escherichia phage vB_Eco_D226 (99% coverage, 99.15% identity).	MLLSDIETNGL
20	middle	ORF20; putative HNH endonuclease	HNH	15280..15615	Pfam: PF13392.6 HNH endonuclease (aa 47-89); BLASTP: HNH endonuclease in Escherichia phage LL2 (98% coverage, 81.82% identity).	MGGVTTLRFH
21	middle	ORF21; putative HNS binding protein	DNA	15602..15886	Pfam: DUF2675 (aa 1-93), Gene protein 5.5 in T7 (UniProt P03787); BLASTP: hypothetical protein Kvp1_gp22 in Kluyvera phage Kvp1 (coverage 98%, identity 100.00%) and HNS binding protein in Klebsiella phage Patroon (97%coverage,	MAITKRFKVSF
22	middle	ORF22; putative inhibitor of the stationary phase	DNA	15886..16095	BLASTP: hypothetical protein in gp5.7 in Enterobacteria phage 285P (98% coverage, 100.00% identity), HNS binding protein in Escherichia phage P483 (98% coverage, 98.55% identity); Uniprot: Fusion protein 5.5/5.7 in Escherichia	MGEYLRVLAAI
23	middle	ORF23; putative recBCD nuclease inhibitor	Evasion host	16274..17188	HMMER and BLASTP: Inihibitor of recBCD nuclease in Escherichia phage P694 (A0A0D3QHR4_9CAUD, e-value 4.3e-19).	MSLHTDNVTV
24	middle	ORF24; putative exonuclease	DNA	17350..17595	HMMER and BLASTP: exonuclease in Kluyvera phage (B6Z9G6_9CAUD, e-value 2.4e-206) and inEnterobacteria phage P694 (A0A0D3QHL2_9CAUD, e-value 7.2e-204). HMMER: HNH endonuclease 45...90.Pfam PF13392	MSAITLKEFYEL
25	late	ORF25; hypothetical conserved protein	Hypothetical	17611..17883	Pfam: DUF2717 (aa 1-78) and disorder (79-82); BLASTP and HMMER: hypothetical protein in Kluyvera phage Kvp1 (B6Z9G7_9CAUD, e-value 2.1e-48)	MLTPIKKYME
26	late	ORF26; hypothetical head protein	Capsid	17898..18263	Pfam: DUF5476 (aa 1-63) and disorder (aa 8-70, 74-84); BLASTP: gp6.7 in Enterobacteria phage 285P (98% coverage, 92.31% identity) and hypothetical protein in Shigella phage VB_Ship_A7 (98% coverage, 86.96% identity); HMMER:	MCFSPKIKTPK
27	late	ORF27; putative tail assembly protein	Tail	18276..19877	Pfam: Bacteriophage T7 virion assembly protein (aa 9-121); BLASTP: tail assembly protein in Erwinia phage FE44 (99% coverage, 86.07% identity) and in Salmonella phage vB_SalM-LPST153 (99% coverage, 66.12% identity); HMMER:	MGWGKKIRKS
28	late	ORF28; putative portal protein	Capsid	19954..20865	HHPRED: viral complex, DNA ejection (6R21_D, e-value 3.7e-60) and portal protein (6QX5_K, e-value 3.2e-56) in Enterobacteria phage T7; Pfam: (aa 21-484); HMMER: Portal protein in Escherichia phage P694 (e-value 0) and in	MASSQKREGF
29	late	ORF29; putative capsid scaffolding protein	Capsid	20999..22042	Pfam: Phage T7 capsid assembly (aa 151-273), coiled coil (aa 110-130) and disorder (aa 1-153); HMMER: Capsid assembly protein in Kluyvera phage Kvp1 (B6Z9H1_9CAUD, e-value 8.2e-189), Capsid and scaffold protein in Escherichia	MAGESNADVY
30	late	ORF30; putative major capsid protein	Capsid	22057..22176	BLASTP: capsid and scaffold protein in Escherichia phage P483 (99% coverage, 95.97% identity) and in Escherichia phage P694 (99% coverage, 94.24% identity); HHPRED: Major capsid protein 10A in Enterobacteria phage T7 (3J7W_E, e-	MANMNGGQ
31	late	ORF31; putative minor caspid protein	Capsid	22249..22839	HMMER: minor capsid protein in Kluyvera phage Kvp1 (B6Z9H2_9CAUD, e-value 2.7e-13), in Yersinia phage Yepe2 (B3VCK8_9CAUD, 2.7e-09), in Escherichia phage C5 (A0A386K6E4_9CAUD, e-value 8.3e-08).	VGDIESLTEVE
32	late	ORF32; putative tail tubular protein A	Tail	22862..25240	BLASTP: tail tubular protein A in Erwinia phage FE44 (99% coverage, 96.94%identity) and in Yersinia phage PYPS50 (99% coverage, 95.92% identity); HHMER: Gp11 in Yersinia phage Yepe2 (B3VCK9_9CAUD, e-value 8.7e-123), tail fiber	MRSYETTLETG
33	late	ORF33; putative tail tubular protein B	Tail	25318..25731	HMMER: Tail tubular protein gp12 in Enterobacteria phage T7 (TUBE2_BPT7, 0.0e+00); HHPRED: viral complex, DNA ejection in Enterobacteria phage T7 (6R21_b, e-value 1.2e-112); BLASTP: tail tubular protein B in Erwinia phage FE44	MALISQSVKNL
34	late	ORF34; putative internal virion protein A	Capsid	25734..26339	Pfam: DUF2833 (aa 39-124); HMMER: internal core protein in Escherichia phage P694 (A0A0D3QHL6_9CAUD, e-value 1.7e-88), internal virion protein A in Kluyvera phage Kvp1 (B6Z9H6_9CAUD, e-value 2.3e-86) and in Pectobacterium	MLIIRPTKESDF
35	late	ORF35; putative internal virion protein B	Capsid	26357..28636	HMMER: Internal virion protein gp14 in Kluyvera phage Kvp1 (B6Z9H7_9CAUD, e-value 8.6e-122) and Enterobacteria phage T7 (GP14_BPT7, e-value 6.2e-42).	MCWMAAIPIA
36	late	ORF36; putative internal virion protein C	Capsid	28660..32607	HMMER: Internal virion protein gp15 in Kluyvera phage Kvp1 (B6Z9H8_9CAUD, e-value 0.0e+00) and in Enterobacteria phage T3 (Q8W5U0_BPT3, e-value 2.2e-297).	MSKLAQALGN
37	late	ORF37; putative peptidoglycan transglycosylase	Capsid	3098..5749	Pfam: Transglycosylase SLT domain (aa 15-120); HMMER: Peptidoglycan transglycosylase gp16 in Kluyvera phage Kvp1 (B6Z9H9_9CAUD, e-value 0.0e+00) and in Enterobacteria phage T7 (EXLYS_BPT7, e-value 0.0e+00); BLASTP: DNA	VDKYNPNEPH
38	late	ORF38; putative tail fiber protein	Tail	32678..34645	Pfam: bacteriophage T7 tail complex (aa 1-154), disorder and three coiled-coil domains; HMMER: tail fibre protein in Enterobacteria phage T7 (FIBER_BPT7, e-value 1.2e-96) and in Enterobacteria phage T3 (Q8W5T8_BPT3, e-value	MANKISTVRTY
39	late	ORF39; putative holin, class II	Lysis	34690..34896	Pfam: Phage holin T7 family, holin superfamily II (aa 4-63); Phobius: transmembrane (aa 37-59); HMMER: Kluyvera phage Kvp1 (B6Z9I2_9CAUD, e-value 3.1e-38) and Enterobacteria phage T3 (Q8W5T7_BPT3, e-value 7.8e-31).	MLSLDFNNEV
40	late	ORF40; putative terminase, small subunit	DNA	34889..35158	Pfam: DNA packaging protein (aa 4-84); HMMER: DNA packaging protein A in Escherichia phage P694 (A0A0D3QHD6_9CAUD, e-value 1.6e-50) and Gp18 in Yersinia phage Yepe2 (B3VCL7_9CAUD, e-value 1.9e-49); BLASTP: terminase	MSNDKSLIQFL
41	late	ORF41; putative i-spanin	Lysis	35257..35697	Pfam: Bacteriophage Rz lysis protein (aa 31-136) and coiled-coil domain; Phobius: signal peptide (aa 1-25); BLASTP: endopeptidase Rz in Escherichia phage vB_Eco_D226 (99% coverage, 92.47%identity) and in Escherichia phage P694	MLKNLKSYAIIA
42	late	ORF42; putative o-spanin	Lysis	35372..35626	Predicted with cpt.fixModel; HMMER: Yersinia phage Yepe2 (score 2.2e-42); domains: signal peptide and outer-membrane spanin sub-unit T7.	MSTLRKLRRLR
43	late	ORF43; hypothetical conserved protein	Hypothetical	35768..36256	HMMER only hit: uncharacterized protein in Yersinia phage Yepe2 (B3VCM0_9CAUD; e-value 4.1e-31); Phobius: two transmembrane domains (aa 12 to 34, 55 to 78).	VGRIDIPASEM
44	late	ORF44; putative terminase, large subunit	DNA	36272..38032	Phobius: two transmemebrane domins (aa 12-34 and 55-78); HMMER: DNA packaging protein B in Kluyvera phage Kvp1 (B6Z9I6_9CAUD, e-value 0.0e+00) and Enterobacteria phage T3 (Q8W5T4_BPT3, e-value 0.0e+00); BLASTP: DNA	MSQSQEAKNA
45	late	ORF45; putative HNH endonuclease	HNH	38062..38505	Pfam: HNH endonuclease (aa 34-76) and disorder domain; HMMER: HNH homing endonuclease in Pectobacterium phage PP74 (A0A1J0MEL6_9CAUD, e-value 9.4e-99), Yersinia phage Yepe2 (B3VCM2_9CAUD, e-value 6.1e-66), Yersinia	VAIDPISGCHN
46	late	ORF46; hypothetical conserved protein	Hypothetical	38751..38906	Phobius: signal peptide (aa 1-33); HMMER: Uncharacterized protein 19.5 in Kluyvera phage Kvp1 (B6Z9I8_9CAUD; e-value 4.3e-24) and Enterobacteria phage T3 (Q778J8_BPT3; e-value 4.1e-08).	MRLLSVLKTLA

B)

NCBI accession number	Phage name	Dcm sites (CCAGG)	Dam sites (GATC)
	Escherichia phage ETEP21B	0	2
NC_047984	Escherichia phage vB_EcoP_S523	0	2
NC_011534	Kluyvera phage Kvp1	1	2
NC_048105	Salmonella phage BSP161	0	0
NC_022744	Erwinia phage FE44	1	1
NC_011040	Enterobacteria phage BA14	0	0
NC_015249	Enterobacteria phage 285P	0	0
MT542512	Escherichia phage PhiV-1	0	2
MT625440	Enterobacter phage PZJ0206	0	2
MZ501064	Escherichia phage ErnstBeyeler strain Bas67	0	5
MZ501055	Escherichia phage CarlSpitteler strain Bas68	0	5
NC_008694	Yersinia phage Berlin	13	9
MN252582	Salmonella phage LPST144	1	1
MK907285	Salmonella phage vB_SalM-LPST153	1	1

C)

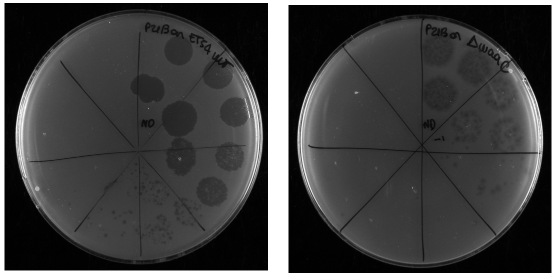

SUPPLEMENTAL FILE S7. A) Full model of T7 tail fiber. B) Schematic topology diagram depicting the second putative receptor-binding domain and the tail tip. C) Top: Predicted Aligned Error Matrices (PAE) of tail fibers from ETEP21B, ETEP102, and T7-gp17, generated from five models. Bottom-left: Sequence coverage plot displaying the coverage of the three fibers. Bottom-right: pLDDT per residue plot, depicting the confidence score for each residue in each fiber prediction.

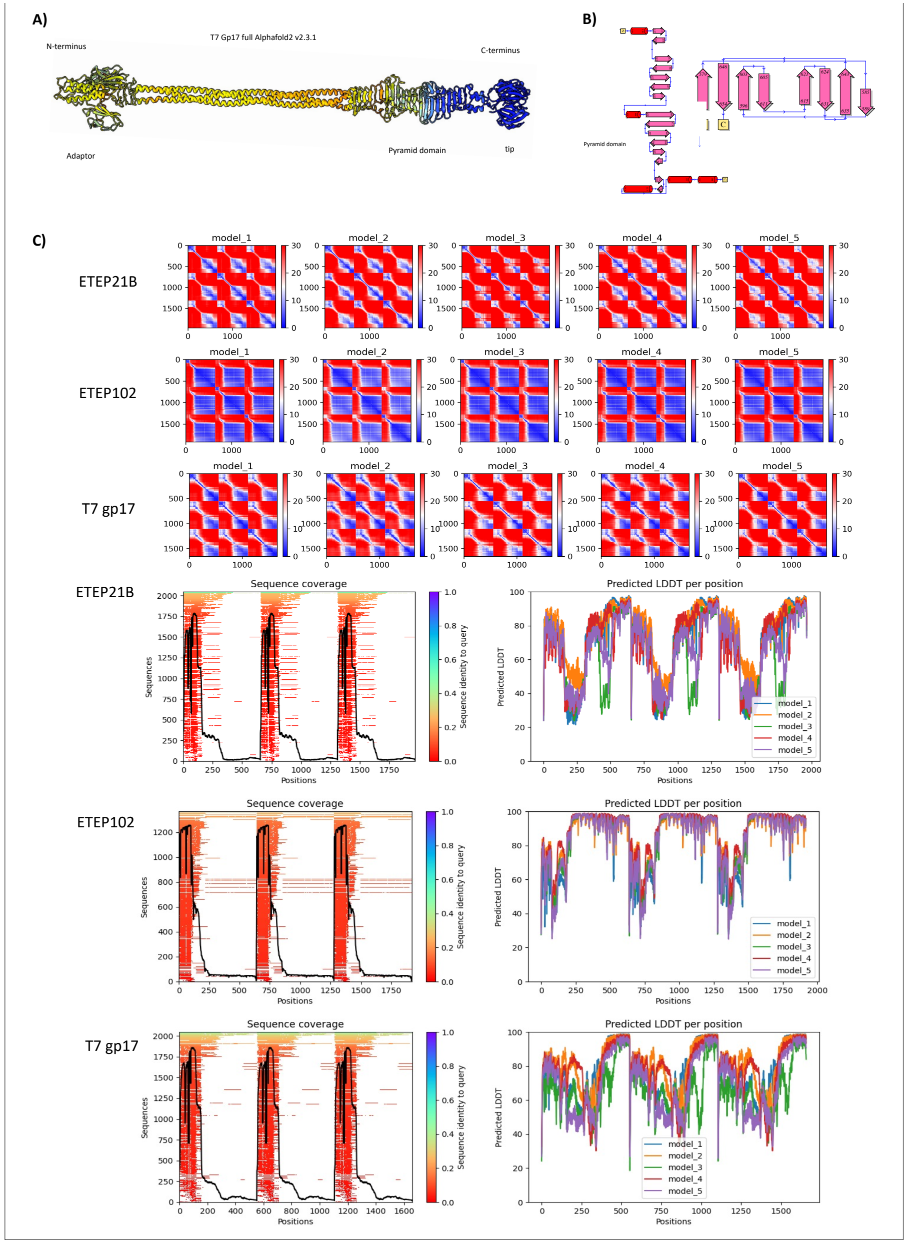

SUPPLEMENTAL FILE S8. ETEP102 full annotation table. The ETEP102 genome encodes for several proteins that play roles in DNA metabolism (ORFs 13, 14, 16, 53, 55, 57), recombination (ORFs 49, 50), but also modification (ORFs 60, DNA adenine methyltransferase – Dam, and 75, DNA cytosine methyltransferase - Dcm), that were expected to confer resistance to restriction by several E. coli enzymes. Since no RNA polymerase was identified and all predicted promoters were host promoters, we speculated that the transcription relied on the host, and that the high number of putative promoters and terminators ensured phage genome transcription, which was the case for phage T1. The lysis cassette (ORFs 68 to 70) was formed by a holin, an endolysin and a unimolecular spanin (u-spanin). The packaging genes (ORFs 21 and 23) were separated by one of the three HNH homing endonucleases identified (ORFs 22) as observed for phage Rtp. The other two HNH homing endonucleases (ORFs 56 and 71) were instead in different positions compared to phage T1 and Rtp, suggesting that these mobile genes generate diversity in the Drexlerviridae family. The structural genes encoded for capsid (ORFs 24 to 33) and tail morphogenesis (ORFs 35 to 46, and 52). Given the high similarity with Rtp and T1 large terminase subunits, ETEP102 DNA was packaged by headful mechanism starting at the pac site, producing terminal redundancy and circular permutation.

ORF	Product	Functional category	Region	Similarities and homologies in databases (InterPro, GeneScan, Pfam, HMMER, BLASTP, HHPRED)	T[illegible]
1	hypothetical protein	Hypothetical	708..827		N
2	conserved hypothetical protein	Hypothetical	891..1427	HMMER: uncharacterized protein in Escherichia phage vB_EcoS_ESCO41 (A0A1U9WQX3_9CAUD, e-value 1.2e-103), Enterobacteria phage vB_EcoS_Rogue1 (K7PL56_9CAUD, e-value 1.7e-45), Escherichia virus Jk06 (Q45PW9_9CAUD, e-value 1.0e-44), Escherichia phage CJ19 (A0A6H0X4S0_9CAUD, e-value 1.2e-29), Escherichia virus Rtp (Q333I3_9CAUD, e-value 2.0e-23)	N
3	conserved hypothetical protein	Hypothetical	1427..1540	HMMER: uncharacterized protein in Escherichia phage e4/1c (A0A023ZUF8_9CAUD, e-value 2.1e-15), Escherichia phage C119 (A0A0P0IR73_9CAUD, e-value 1.5e-13), Escherichia phage vB_EcoS_ESCO41 (A0A1U9WQX9_9CAUD, e-value 5.2e-13), Escherichia phage DTL (A0A2H4PGN1_9CAUD, e-value 9.4e-12), Enterobacteria phage vB_EcoS_Rogue1 (K7PMM5_9CAUD, e-value 5.8e-10), Escherichia virus Rtp (Q333B7_9CAUD, e-value 7.7e-10); RAST: ACLAME 1084, capsid protein; NR: helicase in Escherichia phage Henu8 (score = 1.2779e-06)	N
4	conserved hypothetical protein	Hypothetical	1568..1987	HMMER: uncharacterized protein in Escherichia virus Jk06 (Q45PX0_9CAUD, e-value 4.1e-71), Escherichia phage CJ19 (A0A6H0X4S2_9CAUD, e-value 9.7e-67), Escherichia phage C119 (A0A0P0IR73_9CAUD, e-value 1.1e-65), Escherichia phage DTL (A0A2H4PGN1_9CAUD, e-value 7.7e-18), Escherichia phage vB_EcoS_ESCO41 (A0A1U9WR54_9CAUD, e-value 4.1e-15), Escherichia virus Rtp (Q333B7_9CAUD, e-value 3.2e-14); RAST: ACLAME 1084, capsid protein	N
5	conserved hypothetical protein	Hypothetical	1987..2199	HMMER: uncharacterized protein in Escherichia phage vB_EcoS_ESCO41 (A0A1U9WQX8_9CAUD, e-value 2.6e-35)	N
6	conserved hypothetical protein	Hypothetical	2273..2479	HMMER: uncharacterized protein in Escherichia phage vB_EcoS_ESCO41 (A0A1U9WQX8_9CAUD, e-value 2.6e-35)	N
7	conserved hypothetical protein	Hypothetical	2476..2694	HMMER: uncharacterized protien in Escherichia phage CJ19 (A0A6H0X506_9CAUD, e-value 2.2e-35)	N
8	conserved hypothetical protein	Hypothetical	2687..2887	HMMER: uncharacterized protein in Escherichia phage CJ19 (A0A6H0X4S8_9CAUD, e-value 1.1e-33), Klebsiella phage vB_KpnS_Domnhall (A0A5B9NI00_9CAUD, e-value 1.3e-25), Klebsiella phage KOX1 (A0A1W6JT00_9CAUD, e-value 2.2e-24), Escherichia phage vB_EcoS_NBD2 (A0A192Y7L3_9CAUD, e-value 2.1e-21), Enterobacter phage Ec_L1 (A0A2P0W9X0_9CAUD, e-value 1.0e-20)	N
9	conserved hypothetical protein	Hypothetical	2884..3174	HMMER: uncharacterized protein in Escherichia phage CJ19 (A0A6H0X4R6_9CAUD, e-value 1.0e-55), Escherichia phage vB_EcoS_PHB17 (A0A514DKL6_9CAUD, e-value 3.8e-25)	N
10	conserved hypothetical protein	Hypothetical	3255..3413	HMMER: uncharacterized protein in Escherichia phage vB_EcoS_ESCO41 (A0A1U9WQX7_9CAUD, e-value 7.5e-26), Escherichia phage CJ19 (A0A6H0X4Q5_9CAUD, e-value 6.0e-21)	N
11	conserved hypothetical protein	Hypothetical	3495..4061	HMMER: uncharacterized protein in Escherichia phage vB_EcoS_ESCO41 (A0A1U9WQX7_9CAUD, e-value 1.1e-108), Escherichia phage CJ19 (A0A6H0X4Q5_9CAUD, e-value 1.4e-100)	N
12	hypothetical protein	Hypothetical	4135..4278		N
13	putative polynucleotide kinase/phosphatase	DNA replication/trancription	4278..4895	HMMER: uncharacterized protein in Escherichia phage vB_EcoS_ESCO41 (A0A1U9WQY1_9CAUD, e-value 1.3e-116) and Escherichia phage CJ19 (A0A6H0X4Z6_9CAUD, e-value 1.9e-105), Putative polynucleotide kinase/phosphatase in Escherichia virus Rtp (Q333I0_9CAUD, e-value 1.2e-84) and Escherichia phage T1 (Q6XQF1_BPT1, e-value 6.7e-58); RAST: 3'-phosphatase, 5'-polynucleotide kinase,phage-associated	N
14	putative deoxynucleotide kinase	DNA replication/trancription	4945..5562	InterproScan: P-loop containing nucleoside triphosphate hydrolases (score 4,13E-10); Canonical phages: deoxynucleotide kinase in Escherichai phage T1 (CPT-T1-YP_003883.1, score 2,41639E-44); HMMER: uncharacterized protein in Escherichia phage vB_EcoS_ESCO41 (A0A1U9WQY4_9CAUD, e-value 5.5e-130), Escherichia phage CJ19 (A0A6H0X4R9_9CAUD, e-value 4.6e-120), Escherichia phage DTL (A0A2H4PGS4_9CAUD, e-value 6.7e-65)	N
15	conserved hypothetical protein	Hypothetical	5636..5794	HMMER: uncharacterized protein in Escherichia phage DTL (A0A2H4PGM8_9CAUD, e-value 8.2e-19), Escherichia phage CJ19 (A0A6H0X4Q6_9CAUD, e-value 1.0e-18), Escherichia phage vB_EcoS_ESCO41 (A0A1U9WQY6_9CAUD, e-value 2.2e-17)	N
16	putative methyltransferase	DNA replication/trancription	6033..6254	NR: methyltransferase type 11 in Escherichia phage IMM-001 (gbATI17060.1, score 1,58294E-21); HMMER: uncharacterized protein in Escherichia phage vB_EcoS-IME253 (A0A1P8DUP4_9CAUD, e-value 4.4e-25), Escherichia phage vB_EcoS_ESCO41 (A0A1U9WQY9_9CAUD, e-value 2.8e-24), Escherichia phage CJ19 (A0A6H0X4R8_9CAUD, e-value 4.0e-24)	N
17	conserved hypothetical protein	Hypothetical	6251..6589	HMMER: uncharacterized protein in Escherichia phage CJ19 (A0A6H0X4X7_9CAUD, e-value 2.3e-62), Escherichia phage vB_EcoS_ESCO41 (A0A1U9WR03_9CAUD, e-value 1.3e-59), Escherichia virus TLS (A5PIX9_9CAUD, e-value 5.7e-23)	N
18	conserved hypothetical protein	Hypothetical	6577..6816	HMMER: uncharacterized protein in Escherichia phage CJ19 (A0A6H0X4Q0_9CAUD, e-value 5.1e-37), Escherichia phage vB_EcoS_ESCO41 (A0A1U9WQZ9_9CAUD, e-value 2.8e-33), Escherichia phage DTL (A0A2H4PGM2_9CAUD, e-value 7.3e-23), Escherichia phage vB_EcoS_MM01 (A0A482N4F9_9CAUD, e-value 1.4e-22), Escherichia virus Rtp (A0A482N4F9_9CAUD, e-value 6.4e-21)	N
19	conserved hypothetical protein	Hypothetical	7178..7357	Phobius: signal peptide (aa 1-16); HMMER: uncharacterized protein in Escherichia phage vB_EcoS_ESCO41 (A0A1U9WQZ0_9CAUD, e-value 1.5e-27), Escherichia phage CJ19 (A0A6H0X4Q7_9CAUD, e-value 9.0e-25)	N
20	conserved hypothetical protein	Hypothetical	7357..7578	HMMER: uncharacterized protein in Escherichia phage CJ19 (A0A6H0X4P5_9CAUD, e-value 2.4e-34), Escherichia phage vB_EcoS_ESCO41 (A0A1U9WQZ2_9CAUD, e-value 6.7e-31)	N
21	terminase, small subunit	DNA packaging	7660..8178	Pfam: DNA-packaging protein gp3 (aa 135-149); HMMER: putative terminase small subunit in Enterobacteria phage vB_EcoS_Rogue1 (K7PHG3_9CAUD, e-value 1.0e-65), Escherichia phage C119 (A0A0P0I880_9CAUD, e-value3.6e-65), Escherichia phage DTL (A0A2H4PGM4_9CAUD e-value 5.1e-55), Escherichia phage vB_EcoS_MM01 (A0A482N4Q2_9CAUD, e-value 1.4e-54)	N
22	putative HNH endonuclease	HNH endonucleases	8236..8748	Pfam: HNH endonuclease (aa 61-105); HMMER: HNH endonuclease in Escherichia phage vB_EcoS_AKS96 (A0A067YYU7_9CAUD, e-value 1.1e-58), Salmonella phage IME207 (e-value 4.0e-30); Canonical: very similar to protein 3.8 in Escherichia phage T7 (gnl|BL_ORD_ID|536715 sp|P03797|Y38_BPT7, e-value 5E-07)	N
23	putative terminase large subunit	DNA packaging	8741..10321	Pfam: terminase RNAseH-like domain (aa 352-506); HMMER: Terminase-like family protein in Escherichia phage vB_EcoS_ESCO41 (A0A1U9WR01_9CAUD, e-value 0.0e+00), Escherichia phage CJ19 (A0A6H0X4Q8_9CAUD, e-value 0.0e+00); putative terminase large subunit in Escherichia phage T1 (Q6XQD9_BPT1, e-value 2.5e-223)	N
24	putative protal protein	Capsid morphogenesis	10371..11645	HMMER: Uncharacterized protein in Escherichia phage vB_EcoS-26047II (A0A5P1M743_9CAUD, e-value 4.9e-213) and Escherichia phage vB_EcoS_MM01 (A0A482N6W0_9CAUD, e-value 3.8e-201); putative portal protein in Escherichia virus Rtp (Q333G1_9CAUD, e-value 1.4e-208), Enterobacteria phage vB_EcoS_Rogue1 (K7PHV7_9CAUD, e-value 4.3e-204), Escherichia phage DTL (A0A2H4PGN2_9CAUD, e-value 5.0e-201), Escherichia phage T1 (Q6XQD8_BPT1, e-value 1.1e-141)	N
25	putative capsid morphogenesis protein	Capsid morphogenesis	11602..12339	Phobius: transmembrane (aa 58-78); HMMER: Uncharacterized protein in Escherichia phage vB_EcoS-26047II (A0A5P1M5S5_9CAUD, e-value 2.2e-125), Escherichia phage vB_EcoS_ESCO41 (A0A1U9WQZ6_9CAUD, e-value 4.2e-55); putative phage head morphogenesis protein in Escherichia virus Rtp (Q333G0_9CAUD, e-value 5.3e-110), Enterobacteria phage vB_EcoS_Rogue1 (K7PL62_9CAUD, e-value 2.4e-91); phage_Mu_F domain-containing protein in Escherichia phage T1 (Q6XQD7_BPT1, e-value 2.5e-46)	N
26	putative prohead protease	Capsid morphogenesis	12342..13451	HMMER: DUF2213 (aa 14-146); Phobius: coiled-coil (aa 231-258); Pfam: putative prohead protease in Escherichia phage DTL (A0A2H4PGP3_9CAUD, e-value 3.8e-141) and in Escherichia virus Rtp (Q333F9_9CAUD, e-value 1.3e-140)	N
27	putative capsid decoration protein	Capsid morphogenesis	13463..13942	CPT canonical phages: T1-YP_003896.1 capsid decoration protein; HMMER: uncharacterized protein in Escherichia phage vB_EcoS_FP (A0A7D5FMK3_9CAUD, e-value 8.7e-76), in Escherichia virus Rtp (Q333F8_9CAUD, e-value 1.3e-69). IUPred: disorder (aa 133-134 and 150-157)	N
28	hypothetical protein	Hypothetical	complement(14010..14150)		N
29	putative major capsid protein	Capsid morphogenesis	14210..15160	Pfam: DUF2184 (aa 78-312); HMMER: putative major capsid protein in Escherichia phage vB_EcoD_SU57 (A0A7D5JVL4_9CAUD, e-value 1.6e-179), in Escherichia phage vB_EcoS_MM01 (A0A482N5H1_9CAUD, e-value 7.6e-173), in Escherichia phage e4/1c (A0A023ZU50_9CAUD, e-value 3.8e-172), in Escherichia phage vB_EcoS_CEB_EC3a (A0A1Q1PW97_9CAUD, e-value 4.4e-169), in Escherichia phage vB_EcoS_ACG-M12 (K4FEA0_9CAUD, e-value 4.5e-167), in Escherichia phage vB_EcoS-IME253 (A0A1P8DUQ9_9CAUD, e-value 1.8e-165), in Escherichia phage vB_EcoS_IME542 (A0A410T651_9CAUD, e-value 1.9e-165), in Escherichia virus Rtp (Q333F7_9CAUD, e-value 1.2e-162)	N
30	hypothetical protein	Hypothetical	15245..15490	HMMER: uncharacterized protein in Escherichia phage vB_EcoS_FP (A0A7D5FN27_9CAUD, e-value: 1.7e-28), in Escherichia phage DTL (A0A2H4PGM9_9CAUD, e-value: 3.3e-28) and in Escherichia phage vB_EcoS_CEB_EC3a (A0A1Q1PWC9_9CAUD, e-value: 2.3e-28)	N
31	hypothetical protein	Hypothetical	15523..15618		N
32	putative head-to-tail connector protein	Capsid morphogenesis	15642..16040	CPT canonical phages: T1-YP_003900.1 Head-to-tail connector complex protein/hypothetical protein (e-value 5,5E-40); Pfam: DUF4054 (aa 9-115); HMMER: uncharacterized protein in Escherichia phage vB_EcoS_ESCO41 (A0A1U9WR08_9CAUD, e-value: 1.8e-59), in Escherichia phage vB_EcoS_NBD2 (A0A192Y8A7_9CAUD, e-value: 1.2e-59), in Escherichia phage CJ19 (A0A6H0X4X6_9CAUD, e-value: 1.7e-55)	N
33	putative head-to-tail connector protein	Capsid morphogenesis	16040..16420	(A0A6H0X4P8_9CAUD, e-value: 2.4e-75), putative virion structural protein in Escherichia phage vB_EcoS_NBD2 (A0A192Y8D1_9CAUD, e-value: 2.0e-56)	N
34	conserved hypothetical protein	Hypothetical	16413..16856	HMMER: uncharacterized protein in Escherichia phage CJ19 (A0A6H0X4N7_9CAUD, e-value 2.8e-88), in Escherichia phage vB_EcoS_ESCO41 (A0A1U9WR06_9CAUD, e-value 2.7e-87), Escherichia phage vB_EcoS_NBD2 (A0A192Y7R0_9CAUD, e-value 1.3e-59)	N
35	putative tail protein	Tail morphogenesis	16846..17247	HMMER: uncharacterized protein in Escherichia phage CJ19 (A0A6H0X4M9_9CAUD, e-value 1.0e-74) and Escherichia phage vB_EcoS_ESCO41 (A0A1U9WR24_9CAUD, e-value 1.9e-73), putative structural proteinin Escherichia phage vB_EcoS_NBD2 (A0A192Y9K0_9CAUD, e-value 2.6e-48), putative tail protein Escherichia phage Henu7 (A0A5B8RNU4_9CAUD, e-value 5.9e-41), Phage NBSal001 (A0A6G8QY27_9CAUD, e-value 1.5e-40), Salmonella phage vB_SenS_PHB07 (A0A2R3U9D8_9CAUD, e-value 2.0e-39), Gp41 in Escherichia virus TLS (A5PIZ8_9CAUD, e-value 2.0e-39)	N
36	putative tail protein	Tail morphogenesis	17261..17935	Pfam: DUF4128 (aa 7-128); HMMER: uncharacterized protein in Escherichia phage CJ19 (A0A6H0X4M9_9CAUD, e-value 1.0e-74) and Escherichia phage vB_EcoS_ESCO41 (A0A1U9WR24_9CAUD, e-value 1.9e-73), putative structural protein in Escherichia phage vB_EcoS_NBD2 (A0A192Y9K0_9CAUD, e-value 2.6e-48), putative tail protein in Escherichia phage Henu7 (A0A5B8RNU4_9CAUD, e-value 5.9e-41) and Salmonella phage vB_SenS_PHB07 (A0A2R3U9D8_9CAUD, e-value 2.0e-39), Gp41 in Escherichia virus TLS (A5PIZ8_9CAUD, e-value 2.0e-39)	N
37	putative tape measure chaperone	Tail morphogenesis	18008..18322	Pfam: phage tail assembly chaperone (aa 49-154); HMMER: tape measure chaperone in Escherichia phage CJ19 (A0A6H0X4V5_9CAUD, e-value 1.6e-63), Escherichia phage SRT8 (A0A2D1GPB0_9CAUD, e-value 1.1e-36), Escherichia phage Henu7 (A0A5B8RLL2_9CAUD, e-	N
38	conserved hypothetical protein	Hypothetical	18340..18633	[illegible] (A0A2Z4Q2U7_9CAUD, e-value 5.8e-39), TfmS in Escherichia virus TLS (A5PJ00_9CAUD, e-value 6.0e-39)	N
39	putative tape measure protein	Tail morphogenesis	18671..21673	[illegible] Escherichia phage CJ19 (A0A6H0X4N4_9CAUD, e-value 0.0e+00), Escherichia phage vB_EcoS_ESCO41 (A0A1U9WR13_9CAUD, e-value 0.0e+00), Escherichia phage Henu7 (A0A5B8RP62_9CAUD, e-value 0.0e+00), Escherichia virus TLS (A5PJ03_9CAUD, e-value 0.0e+00)	N
40	putative minor tail protein	Tail morphogenesis	21677..22024	Pfam: minor tail protein (aa 4-112); HMMER: minor tail protein in Escherichia phage vB_EcoS_ESCO41 (A0A1U9WR05_9CAUD, e-value 1.0e-71), Escherichia phage CJ19 (A0A6H0X4N8_9CAUD, e-value 5.3e-71), Escherichia phage vB_EcoS_NBD2 (A0A192Y9K7_9CAUD, e-value 4.9e-56), Escherichia phage T1 (Q6XQC3_BPT1, e-value 1.6e-38)	N
41	putative minor tail protein	Tail morphogenesis	22067..22813	Pfam: minor tail protein L (aa 26-243); HMMER: minor tail protein in Escherichia phage CJ19 (A0A6H0X4M5_9CAUD, e-value 1.0e-163), Escherichia phage vB_EcoS_ESCO41 (A0A1U9WR11_9CAUD, e-value 2.2e-161), Escherichia phage vB_EcoS_NBD2 (A0A192YAL4_9CAUD, e-value 1.2e-138), ThmL in Escherichia virus TLS (A5PJ05_9CAUD, e-value 6.3e-129)	N
42	putative minor tail protein	Tail morphogenesis	22810..23535	[illegible] 165), Escherichia phage vB_EcoS_NBD2 (A0A192Y8C7_9CAUD, e-value 6.2e-141), Salmonella phage phSE-2 (A0A160CA77_9CAUD, e-value 1.8e-131), Salmonella phage GJL01 (A0A1V0DYY0_9CAUD, e-value 2.1e-130)	N
43	putative tail assembly protein	Tail morphogenesis	23529..24122	Pfam: Lamda tail assembly rpotin (aa 1-86); Phobius: transmembrane (aa 88-106 and 112-138); HMMER: tail assembly protein in Escherichia phage vB_EcoS_ESCO41 (A0A1U9WR18_9CAUD, e-value 1.3e-123), Escherichia phage CJ19(A0A6H0X4N1_9CAUD, e-value 4.4e-121), Escherichia phage vB_EcoS_NBD2(A0A192Y8F2_9CAUD, e-value 3.7e-109), Shigella phage vB_SsoS_008 (A0A411AVD4_9CAUD, e-value 2.1e-99), Escherichia phage T1 (Q6XQC0_BPT1, e-value 2.3e-99)	N
44	putative tail tip protein	Tail morphogenesis	24200..27580	Pfam: phage tail protein (aa 339-507), DUF1983 (aa 956-1038) and coiled coil (aa 899-919); HMMER: Fibronectin type-III domain-containing protein in Escherichia phage vB_EcoS_ESCO41 (A0A1U9WR17_9CAUD, e-value 0.0e+00) and Escherichia phage CJ19, similar to many comnserved Lambda J-like proteins (A0A6H0X4M7_9CAUD, e-value 0.0e+00)	N
45	putative tail tip protein	Tail morphogenesis	complement(27609..28565)	al. PLOS Biology 2021)	N
46	putative tail tip protein	Tail morphogenesis	complement(28565..28807)	Phobius: signal peptide (aa 1-23); HMMER: in Escherichia phage vB_EcoS_ESCO41 (A0A1U9WR16_9CAUD, e-value 3.2e-43), Escherichia phage vB_EcoS_CEB_EC3a (A0A1Q1PWC8_9CAUD, e-value 9.7e-43), Escherichia phage 2725-N35 (A0A6B9SP91_9CAUD, e-value 2.0e-39), Enterobacteria phage vB_EcoS_Rogue1 (K7PHH9_9CAUD, e-value 9.4e-39); predicted to encode the RBP binding to the secondary receptor (Maffei et al. PLOS Biology 2021)	N
47	hypothetical protein	Hypothetical	complement(29078..29167)		L
48	hypothetical protein	Hypothetical	complement(29207..29296)		V
49	putative exonuclease	Recombination	29323..30363	Pfam: PDDEXK-like domain of unknown function DUF3799, likely to be nuclease (aa 217-329); RAST: phage exonuclease; HMMER: DUF3799 containing protein in Escherichia phage CJ19 (A0A6H0X4U5_9CAUD, e-value 3.7e-230), Escherichia phage vB_EcoS_ESCO41 (A0A1U9WR23_9CAUD, e-value 1.7e-229), exonuclease in Escherichia phage Henu8 (A0A5B8RRA3_9CAUD, e-value 4.5e-158)	N
50	putative recombinase	Recombination	30440..31096	[illegible] value 2.4e-132) and Escherichia phage CJ19 (A0A6H0X4L3_9CAUD, e-value 2.7e-130), putative recombination protein in Escherichia phage vB_EcoS_NBD2 (A0A192Y8D8_9CAUD, e-value 6.8e-80), Klebsiella phage vB_KpnS_FZ10 (A0A4D6T4X0_9CAUD, e-value 6.8e-73)	N
51	conserved hypothetical protein	Hypothetical	31135..31581	HMMER: uncharacterized protein in Escherichia phage CJ19 (A0A6H0X4M6_9CAUD, e-value 3.2e-83) and Escherichia phage vB_EcoS_ESCO41 (A0A1U9WR22_9CAUD, e-value 6.4e-79), putative ssDNA-binding protein in Escherichia phage vB_EcoS_NBD2 (A0A192Y8G1_9CAUD, e-value 4.9e-53)	N
52	putative lateral tail fiber protein	Tail morphogenesis	complement(31617..33536)	HMMER: tail fiber protein in Escherichia phage CJ19 (A0A6H0X4M8_9CAUD, e-value 0.0e+00) and Escherichia phage DTL (A0A2H4PGQ5_9CAUD, e-value 1.8e-91), uncharacterized protein in Escherichia phage vB_EcoS_MM01 (A0A482N5Z1_9CAUD, e-value 1.4e-91), tail spike protein in Escherichia phage vB_EcoD_SU57 (A0A7D5JMR3_9CAUD, e-value 2.9e-85)	N
53	putative DNA primase	DNA replication/trancription	complement(33626..34549)	Pfam: zinc-binding domain primase/helicase (aa 34-73); HMMER: DNA primase/helicase in Escherichia phage CJ19 (A0A6H0X4L6_9CAUD, e-value 8.8e-202), Escherichia phage vB_EcoS-IME253 (A0A1P8DUU4_9CAUD, e-value 6.7e-197), Escherichia virus Rtp (Q333D3_9CAUD, e-value 3.6e-192)	N
54	conserved hypothetical protein	Hypothetical	complement(34607..35080)	HMMER: uncharacterized protein in Escherichia phage CJ19 (A0A6H0X4V4_9CAUD, e-value 1.5e-99), Escherichia phage vB_EcoS_ESCO41 (e.value 4.4e-98), transcriptional regulator in Escherichia phage vB_EcoS_CEB_EC3a (A0A1Q1PWA9_9CAUD, e-value 2.8e-94), Escherichia virus Rtp (Q333D2_9CAUD, e-value 1.8e-93)	N
55	putative DNA helicase	DNA replication/trancription	35173..37275	Pfam: helicase conserved C-terminal domain (aa 340-449); HMMER: Escherichia phage CJ19 [illegible], Escherichia phage vB_EcoS_NBD2 [illegible], Escherichia phage vB_EcoS_PHB17 (A0A514DKV7_9CAUD, e-value 9.1e-305), Escherichia phage T1 (Q6XQA8_BPT1, 5.2e-296)	N
56	putative HNH endonuclease	HNH endonucleases	37272..37769	Pfam: HNH endonuclease (aa 49-94); HMMER: HNH endonuclease in Salmonella virus VSt472 (A0A385EF75_9CAUD, e-value 1.3e-49), Escherichia phage Henu8 (A0A5B8RMB0_9CAUD, e-value 4.6e-45), Escherichia phage vB_EcoS_ESCO41 (A0A1U9WR09_9CAUD, e-value 1.4e-42)	N
57	putative endonuclease protein	DNA replication/trancription	37753..38184	Pfam: VRR-NUC domain (aa 21-128), in restriction endonuclease-like fold families; HMMER: VRR-NUC domain-containing protein in Escherichia phage CJ19 (A0A6H0X4L8_9CAUD, e-value 2.5e-87), Cronobacter phage PhiCS01 (A0A3B8E508_9CAUD, e-value 2.6e-60), Escherichia phage SRT8 (A0A2D1GPC4_9CAUD, e-value 1.4e-54)	N
58	conserved hypothetical protein	Hypothetical	38251..38523	Phobius: signal peptide (aa 1-17); HMMER: uncharacterized protein in Escherichia phage CJ19 (A0A6H0X4L1_9CAUD, e-value 2.0e-50) and Escherichia phage vB_EcoS_ESCO41 (A0A1U9WR30_9CAUD, e-value 2.0e-50)	N
59	conserved hypothetical protein	Hypothetical	38525..38656		N
60	DNA adenine methyltransferase	DNA replication/trancription	38659..39387	Pfam: DNA N-6-adenine-methyltransferase (Dam) (aa 54-231), possibly for protection from RM system; HMMER: DNA N-6-adenine-methyltransferase in Escherichia phage CJ19 (A0A6H0X4N0_9CAUD, e-value 1.1e-160), Escherichia phage vB_EcoS_ESCO41 (A0A1U9WR31_9CAUD, e-value 4.9e-152), Escherichia phage vB_EcoS_NBD2 (A0A192Y7U5_9CAUD, e-value 5.8e-147)	N
61	conserved hypothetical protein	Hypothetical	39389..39619	HMMER: uncharacterized protein in Escherichia phage vB_EcoS_ESCO41 (A0A1U9WR34_9CAUD, e-value 1.3e-44), Escherichia phage CJ19 (A0A6H0X4T5_9CAUD, e-value 1.1e-43)	N
62	conserved hypothetical protein	Hypothetical	39690..39923	HMMER: uncharacterized protein in Escherichia phage CJ19 (A0A6H0X4K3_9CAUD, e-value 2.0e-46) and Escherichia phage vB_EcoS_ESCO41 (A0A1U9WR39_9CAUD, e-value 2.2e-37)	N
63	conserved hypothetical protein	Hypothetical	39910..40155	HMMER: Escherichia phage CJ19 (A0A6H0X4L7_9CAUD, e-value 1.3e-46) and Escherichia phage vB_EcoS_ESCO41 (A0A1U9WR41_9CAUD, e-value 8.9e-46)	N
64	conserved hypothetical protein	Hypothetical	40152..40403	HMMER: uncharacterized protein in Escherichia phage vB_EcoS_ESCO41 (A0A1U9WR35_9CAUD, e-value 6.2e-48)	N
65	conserved hypothetical protein	Hypothetical	40485..41627	HMMER: uncharacterized protein in Escherichia phage CJ19 (A0A6H0X4T3_9CAUD, e-value 7.9e-248), Enterobacter phage Ec_L1 (A0A2P0W9U2_9CAUD, e-value 2.0e-223), putative phosphoesterase in Escherichia phage vB_EcoS_NBD2 (A0A192Y7V3, e-value 9.8e-220)	N
66	conserved hypothetical protein	Hypothetical	41698..41871	HMMER: uncharacterized protein in Escherichia phage vB_EcoS_ESCO41 (A0A1U9WR44_9CAUD, e-.value 1.2e-28), Escherichia phage CJ19 (A0A6H0X528_9CAUD, e-value 1.2e-28), Escherichia phage vB_EcoS_CEB_EC3a (A0A1Q1PWE8_9CAUD, e-value 1.5e-27), Escherichia virus Rtp (Q333C2_9CAUD, e-value 5.5e-27)	N
67	putative holin	Lysis	42003..42218	Phobius: transmembrane domain (aa 25-45); HMMER: putative holin in Escherichia phage vB_EcoS_CEB_EC3a (A0A1Q1PWD4_9CAUD, e-value 2.8e-38) and Escherichia virus Rtp (Q333C1_9CAUD, e-value 5.8e-37)	N
68	putative lysozyme	Lysis	42219..42704	Pfam: phage lysozyme domain (aa 39-150) and signal peptide (aa 1-27); RAST: Phage lysin (EC 3.2.1.17) # Phage lysozyme ormuramidase (EC 3.2.1.17); HMMER: Escherichia phage vB_EcoS_ESCO41 (A0A1U9WR38_9CAUD, e-value 3.3e-94), Escherichia phage DTL	N
69	putative u-spanin	Lysis	42680..43069	Phobius: signal peptide (aa 1-19) and transmembrane domain (aa 104-126); CPT canonical phages: u-spanin in Escherichia phage T1 (T1-YP_003934.1, e-value 3,64E-24; Summer et al. JMB 2007); HMMER: uncharacterized protein in Escherichia phage CJ19 (A0A6H0X4T1_9CAUD, e-value 2.5e-51), Escherichia phage vB_EcoS_ESCO41 (A0A1U9WR36_9CAUD, e-value 8.2e-51), Escherichia virus TLS (A5PJ27_9CAUD, e-value 3.6e-35), u-spanin in Escherichia phage T1 (SPANU_BPT1, e-value 2.9e-19)	N
70	hypothetical protein	Hypothetical	complement(43233..43607)	Pfam: SH3 (aa 12-78) and SH2 (aa 72-147) domains, in Eukaryota they serve as adaptors to link tyrosine kinases to specific target proteins and participate in the control of intracellular responses (Koch et al. 1991 Science).	N
71	putative HNH endonuclease	HNH endonucleases	complement(43610..43888)	Pfam: HNH endonuclease domain (aa 1-28); HMMER: HNHc domain-containing protein/HNH endonuclease in Escherichia phage vB_EcoD_SU57 (A0A7D5G2S7_9CAUD, e-value 3.1e-19),	N
72	conserved hypothetical protein	Hypothetical	complement(43910..44092)	HMMER: hypothetical protein in Salmonella phage LPST10 (A0A1W6DXY0_9CAUD, e-value 0.027), Escherichia phage SUSP1 (A0A0N9SKZ3_9CAUD, e-value 0.6)	N
73	conserved hypothetical protein	Hypothetical	complement(44067..45650)	Pfam: DUF3987 (aa 79-444); HMMER: uncharacterized protein in Escherichia phage vB_EcoS_ESCO41 (A0A1U9WR54_9CAUD, e-value 0.0e+00), Escherichia phage CJ19 (A0A6H0X4T0_9CAUD, e-value 0.0e+00), Escherichia phage vB_EcoS-IME253 (A0A1P8DUN0_9CAUD, e-value 0.0e+00), Escherichia phage DTL (A0A2H4PGL9_9CAUD, e-value 0.0e+00), Escherichia virus Rtp (Q333B7_9CAUD, e-value 0.0e+00)	N
74	conserved hypothetical protein	Hypothetical	complement(45721..46074)	HMMER: uncharacterized protein in Escherichia phage vB_EcoS_ACG-M12 (K4FBU8_9CAUD, e-value 6.0e-48), Escherichia phage CJ19 (A0A6H0X4T2_9CAUD, e-value 1.2e-47), Escherichia virus Rtp (Q333B5_9CAUD, e-value 7.2e-47), Escherichia phage vB_EcoS_ESCO41 (A0A1U9WR45_9CAUD, e-value 2.3e-45), Escherichia phage DTL (A0A2H4PGK2_9CAUD, e-value 1.0e-43)	N
75	putative cytosine DNA methylase	DNA replication/trancription	complement(46156..46830)	Canonical phages: putative C-5 cytosine DNA methylase in phage EcoS-95 (score 1.7 E-107); HMMER: Putative cytosine DNA methylase in Shigella phage Sd1 (A0A291AYP8_9CAUD, e-value 3.3e-150) and Salmonella phage 9NA (A0A060DBH2_9CAUD, e-value 3.1e-99), uncharacterized protein in Escherichia phage CJ19 (A0A6H0X4T8_9CAUD, e-value 7.9e-127) and Escherichia phage vB_EcoS_ESCO41 (A0A1U9WR58_9CAUD, e-value 8.9e-12)	N
76	conserved hypothetical protein	Hypothetical	complement(46827..46985)	HMMER: uncharacterized protein in Escherichia phage vB_EcoS_ESCO41 (A0A1U9WR57_9CAUD, e-value 6.2e-25), Escherichia phage CJ19 (A0A6H0X4S4_9CAUD, e-value 8.9e-25), Enterobacteria phage vB_EcoS_Rogue1 (K7PMM9_9CAUD, e-value 3.4e-11)	N
77	conserved hypothetical protein	Hypothetical	complement(46978..47223)	HMMER: uncharacterized protein in Escherichia virus Rtp (Q333B1_9CAUD, e-value 2.3e-47), Escherichia phage DTL (A0A2H4PGL1_9CAUD, e-value 3.1e-47)	N
78	conserved hypothetical protein	Hypothetical	complement(47233..47448)	HMMER: uncharacterized protein in Escherichia phage vB_EcoS_ESCO41 (A0A1U9WR48_9CAUD, e-value 1.0e-42), Escherichia phage CJ19 (A0A6H0X4U0_9CAUD, e-value 4.9e-42), Escherichia phage T1 (Q6XQ92_BPT1, e-value 3.4e-15)	N
79	conserved hypothetical protein	Hypothetical	complement(47451..47675)	HMMER: uncharacterized protein in Escherichia phage vB_EcoS_ESCO41 (A0A1U9WR43_9CAUD, e-value 3.2e-39), Escherichia phage CJ19 (A0A6H0X4S6_9CAUD, e-value 8.8e-39), Escherichia phage T1 (Q6XQ91_BPT1, e-value 4.9e-13)	N
80	conserved hypothetical protein	Hypothetical	complement(47757..48188)	HMMER: uncharacterized protein in Escherichia phage CJ19 (A0A6H0X4S1_9CAUD, e-value 2.5e-92) >A0A1U9WR60_9CAUDEscherichia phage vB_EcoS_ESCO415.3e-69	N
81	hypothetical protein	Hypothetical	complement(48200..48415)		N

SUPPLEMENTAL FILE S9. A) Mutations in ET03 resistant to ETEP102: isolated clone sequenced (A or C); mapping on reference (ET03 wild type contig), reference position (position on ET03 wild type); type of mutation (insertion, deletion, replacement, or single nucleotide variant, SNV), length, nucleotide sequence in reference genome, nucleotide sequence in mutant genome, count, coverage, annotation according to the refence genome, frequency of mutation. B) Mutations with more than 65% coverage and predicted amnoacid change: a silent mutation in the sugar ABC transporter ATP-binding protein YphE (ACG to ACA, both wild type and mutated codons encoding Thr); a missense mutation (TTG to TTT, from Leu to Phe, from branched to aromatic amino acid) in a gene expected to encode a D-alanyl-D-alanine carboxypeptidase, a membrane enzyme involved in the peptidoglycan biosynthesis pathway (BLASTP: WP_042027825.1, 100% Query Coverage, E value 0.0, 99.79% identity); three high frequency (100%) and missense mutations (GCG to GTG, from Alanine to Valin in position 274; CAT to CGC, from Histidine to Arginine in position 294; GCT to TCT, from Alanine to Serine in position 305) in a gene with high similarity to traI (HHpred: 5N8O_A, with 99.96% probability, 3.1e-27 E-value, Score 250.05), a secreted DNA helicase and relaxase, essential for conjugation. By comparison with the previously described full length structure of TraI (Ilangovan et al. 2017; HHpred 5N8O_A Cryo EM structure), the mutations fall into the active helicase domain, and the C-terminal domain that recruits the relaxosome components. C) Sensitivity to ETEP102 of knock out mutants in the three candidate genes identified by sequencing analysis, i.e. *yphE*, D-alanyl-D-alanine carboxypeptidase(DaDa) and *traI*, compared to the wild type (wt).

A)

[illegible]

B)

Isolate	Mapping on reference	Reference Position	Type	Length	Ref.	Mut.	Count	Cover.	Annotation	Frequency	Aminoacid change
C	merged_contig_31	4493	SNV	1	C	T	42	42	IncF plasmid conjugative transfer DNA-nicking and unwinding protein TraI	100	from GCG to GTG, from Ala to Val
A	merged_contig_31	4493	SNV	1	C	T	47	47	IncF plasmid conjugative transfer DNA-nicking and unwinding protein TraI	100	from GCG to GTG, from Ala to Val
C	merged_contig_31	4550	MNV	2	AT	GC	34	37	IncF plasmid conjugative transfer DNA-nicking and unwinding protein TraI	91.89189189	from CAT to CGC, from His to Arg
A	merged_contig_31	4550	MNV	2	AT	GC	45	47	IncF plasmid conjugative transfer DNA-nicking and unwinding protein TraI	95.74468085	from CAT to CGC, from His to Arg
C	merged_contig_31	4582	SNV	1	G	T	31	36	IncF plasmid conjugative transfer DNA-nicking and unwinding protein TraI	86.11111111	from GCT to TCT, from Ala to Ser
A	merged_contig_31	4582	SNV	1	G	T	46	49	IncF plasmid conjugative transfer DNA-nicking and unwinding protein TraI	93.87755102	from GCT to TCT, from Ala to Ser
C	merged_contig_31	4662	SNV	1	T	C	36	41	non coding	87.80487805	
A	merged_contig_31	4662	SNV	1	T	C	52	54	non coding	96.2962963	
A	merged_contig_47	420	SNV	1	G	T	132	132	D-alanyl-D-alanine carboxypeptidase (EC 3.4.16.4)	100	from TTG to TTT, from Leu to Phe
A	merged_contig_92	48766	SNV	1	G	A	73	73	Predicted sugar ABC transport system, ATP-binding protein YphE	100	from ACG to ACA, from Thr to Thr

C)

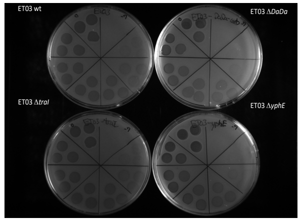

SUPPLEMENTAL FILE S10. ET54 mutants in known phage receptors: *ompA*, *ompC*, *ompF*, *btuB*, *fadL*, *lamB*, *tolC*, *fhuA*, *tsx*, *waaC*, *waaR*.

Primers for deletion fragment amplification	
ompA-Up	ATGAAAAAGACAGCTATCGCGATTGCAGTGGCACTGGCTGGTTTCGCTAC*AATTAACCCTCACTAAAGGGCG*
ompA-Dw	TTAAGCCTGCGGCTGAGTTACAACGTCTTTGATACCTTTAACTTCGATCT*TAATACGACTCACTATAGGGCTC*
ompC-Up	ATGAAAGTTAAAGTACTGTCCCTCCTGGTCCCAGCTCTGCTGGTAGCAGG*AATTAACCCTCACTAAAGGGCG*
ompC-Dw	TTAGAACTGGTAAACCAGACCCAGAGCTACGATGTTATCAGTGTTGATGC*TAATACGACTCACTATAGGGCTC*
ompF-Up	ATGATGAAGCGCAATATTCTGGCAGTGATCGTCCCTGCTCTGTTAGTAGC*AATTAACCCTCACTAAAGGGCG*
ompF-Dw	TTAGAACTGGTAAACGATACCCACAGCAACGGTGTCGTCTGAACCTACGC*TAATACGACTCACTATAGGGCTC*
btuB-Up	CCGCTTGGGCACAGGATACCAGCCCGGATACTCTCGTCGTTACTGCTAACC*AATTAACCCTCACTAAAGGGCG*
btuB-Dw	ATCGATCAAGTCACTGACATCGTTACGATATCCGGAAATACGCCAGTTCA*TAATACGACTCACTATAGGGCTC*
fadL-Up	ATGAGCCAGAAAACCCTGTTTACAAAGTCTGCTCTCGCAGTCGCAGTGGC*AATTAACCCTCACTAAAGGGCG*
fadL-Dw	TCAGAACGCGTAGTTAAAGTTAGTACCGAACAGCCAGGCTTTACCTTCAG*TAATACGACTCACTATAGGGCTC*
lamB-Up	ATGATGATTACTCTGCGCAAACTTCCTCTGGCGGTTGCCGTCGCAGCGGG*AATTAACCCTCACTAAAGGGCG*
lamB-Dw	TTACCACCAGATTTCCATCTGGGCACCGAAGGTCCACTCGTCGCTGTCGC*TAATACGACTCACTATAGGGCTC*
tolC-Up	ATGAAGAAATTGCTCCCCATTCTTATCGGCCTGAGCCTTTCTGGGTTCAG*AATTAACCCTCACTAAAGGGCG*
tolC-Dw	TCAGTTACGGAAAGGGTTATGACCGTTACTGGTGGTAGTGCGTGCGGATG*TAATACGACTCACTATAGGGCTC*
fhuA-Up	ATGGCGCGTTCCAAAACTGCTCAGCCAAAACACTCACTGCGTAAAATCGC*AATTAACCCTCACTAAAGGGCG*
fhuA-Dw	TTAGAAACGGAAGGTTGCGGTTGCAACGACCTGACGTTCTGCGCCCCAGA*TAATACGACTCACTATAGGGCTC*
tsx-Up	ATGAAAAAAACATTACTGGCAGCCGGTGCGGTACTGGCGCTCTCTTCGTC*AATTAACCCTCACTAAAGGGCG*
tsx-Dw	TCAGAAGTTGTAACCTACTACCAGGTAACCACCCCAGCCGGTAGAGCGAA*TAATACGACTCACTATAGGGCTC*
waaC-Up	ATGCGGGTTTTGATCGTTAAAACATCGTCGATGGGCGATGTTCTCCATAC*AATTAACCCTCACTAAAGGGCG*
waaC-Dw	TTAAATCAATGTGATTTTTTTATAAACAGCCGCTGCTTCTAAATTATTTA*TAATACGACTCACTATAGGGCTC*
waaR-Up	ATGAATGAATTTATAAAAGAACGGTTTTCGTATTTAGCAGATAATAAAAA*AATTAACCCTCACTAAAGGGCG*
waaR-Dw	TTATTTCTTAAGCTTGTACTTAATTAATGAAGTTATGCCTTTTATATACT*TAATACGACTCACTATAGGGCTC*
Primers for deletion control	
ompA_F	ATGAAAAAGACAGCTATCGCGATTG
ompA_R	TTAAGCCTGCGGCTGAGTTACAAC
ompC_F	ATGAAAGTTAAAGTACTGTCCCTCC
ompC_R	TTAGAACTGGTAAACCAGACCCAG
ompF_F	ATGATGAAGCGCAATATTCTGGC
ompF_R	TTAGAACTGGTAAACGATACCCACAG
btuB_F	ATGATTAAAAAAGCTTCGCTGCTGAC
btuB_R	TCAGAAGGTGTAGCTGCCAGACAAG
fadL_F	ATGAGCCAGAAAACCCTGTTTACAAAG
fadL_R	TCAGAACGCGTAGTTAAAGTTAGTAC
lamB_F	ATGATGATTACTCTGCGCAAACTTC
lamB_R	TTACCACCAGATTTCCATCTGGG
tolC_F	ATGAAGAAATTGCTCCCCATTCTTATC
tolC_R	TCAGTTACGGAAAGGGTTATGACC
fhuA_F	ATGGCGCGTTCCAAAACTGC
fhuA_R	TTAGAAACGGAAGGTTGCGGTTG
tsx_F	ATGAAAAAAACATTACTGGCAGCC
tsx_R	TCAGAAGTTGTAACCTACTACCAGG
waaC_F	ATGCGGGTTTTGATCGTTAAAACATC
waaC_R	TTAAATCAATGTGATTTTTTTATAAACAGCCG
waaR_F	ATGAATGAATTTATAAAAGAACGGTTTTCG
waaR_R	TTATTTCTTAAGCTTGTACTTAATTAATGAAGTTA
